# Supplementary material for: Lipid droplet-associated hydrolase mobilizes stores of liver X receptor sterol ligands and protects against atherosclerosis
Source: Nat Commun. 2024 Aug 2;15:6540. doi: 10.1038/s41467-024-50949-y (PMC11297204; doi:10.1038/s41467-024-50949-y)
Supplement: Supplementary file 1 — Supplementary Information [file 41467_2024_50949_MOESM1_ESM.pdf]

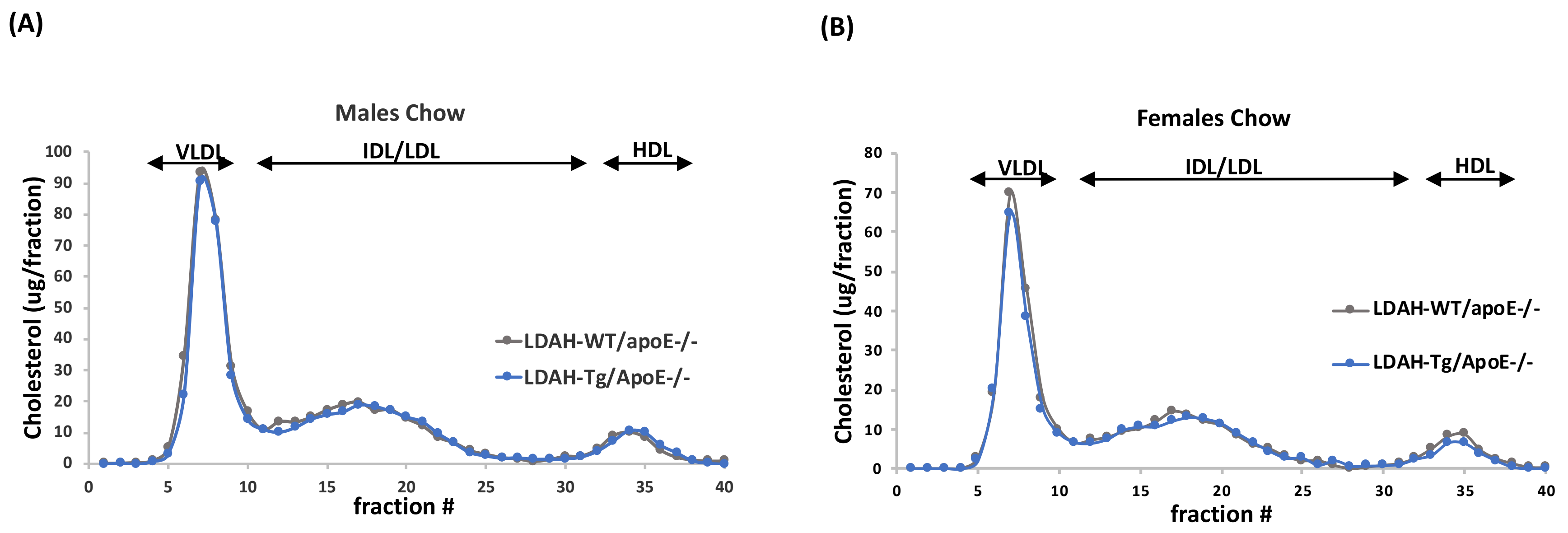


**Figure S1.** Cholesterol distribution in FPLC fractions of pooled plasma of wild-type (LDAH-WT/apoE-/-) and LDAH transgenic (LDAH-Tg/apoE-/-) mice used for atherosclerosis studies. **(A)** Males fed regular chow. **(B)** Females fed regular chow. VLDL: very low-density lipoprotein; IDL/LDL: intermediate-density lipoprotein/low-density lipoprotein; HDL: high-density lipoprotein.

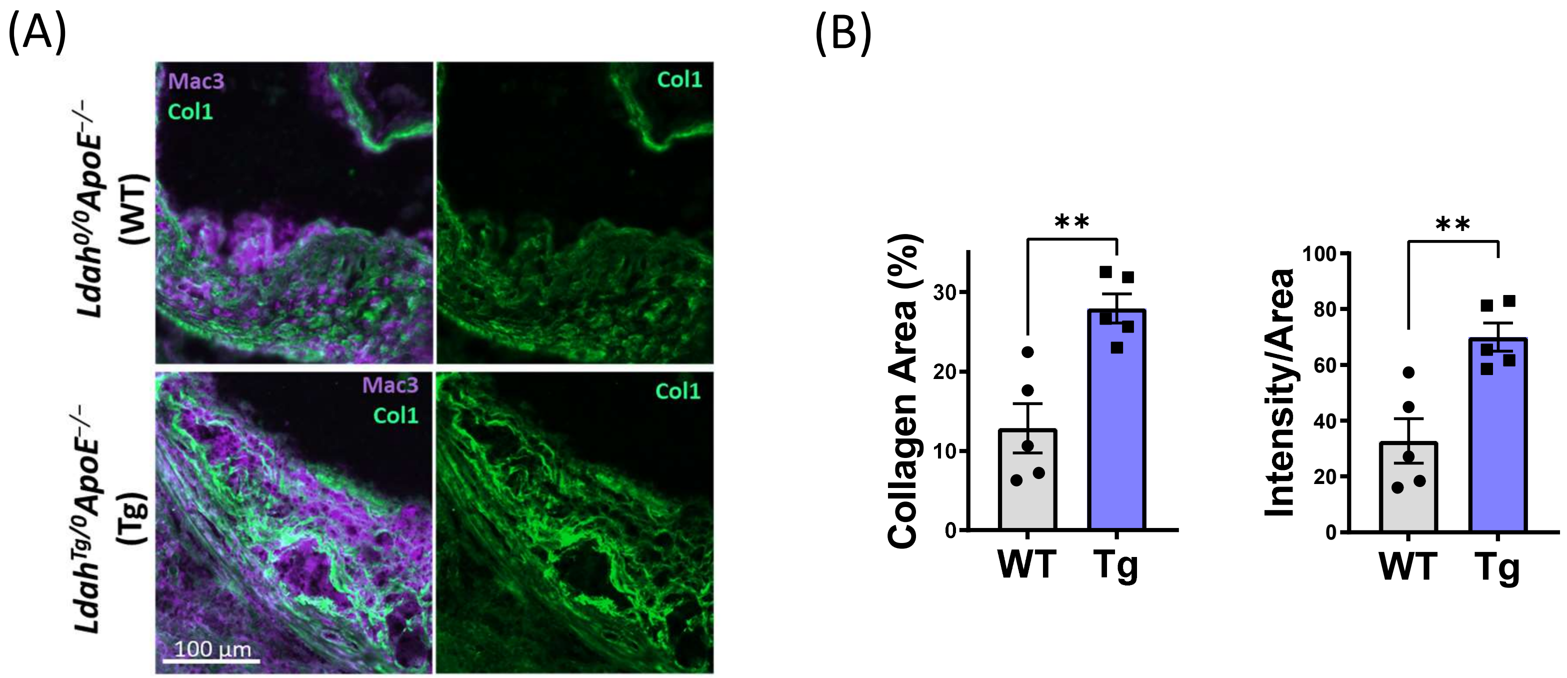


**Figure S2. (A )** Representative images showing increased fibrillar structures that stained positive with an anti-type I collagen antibody (green) in atherosclerotic lesions (outlined in white) of *Ldah*$^{Tg/0}$*ApoE*$^{-/-}$ (Tg) male mice that are predominantly composed of Mac-3 positive cells (purple). Scale bar = 100 µm. (**B**) Quantification of collagen I-positive areas (left panel) and of fluorescence intensity (right panel) in lesions of Tg (blue bars) and WT (grey bars) mice. Data are presented as mean ± SEM from n=5 independent samples per group. Comparisons were performed using two-tailed unpaired *t*-test. $^{**}P$= 0.0032 for Collagen Area (%) and $^{**}P$= 0.0043 for Intensity/Area.

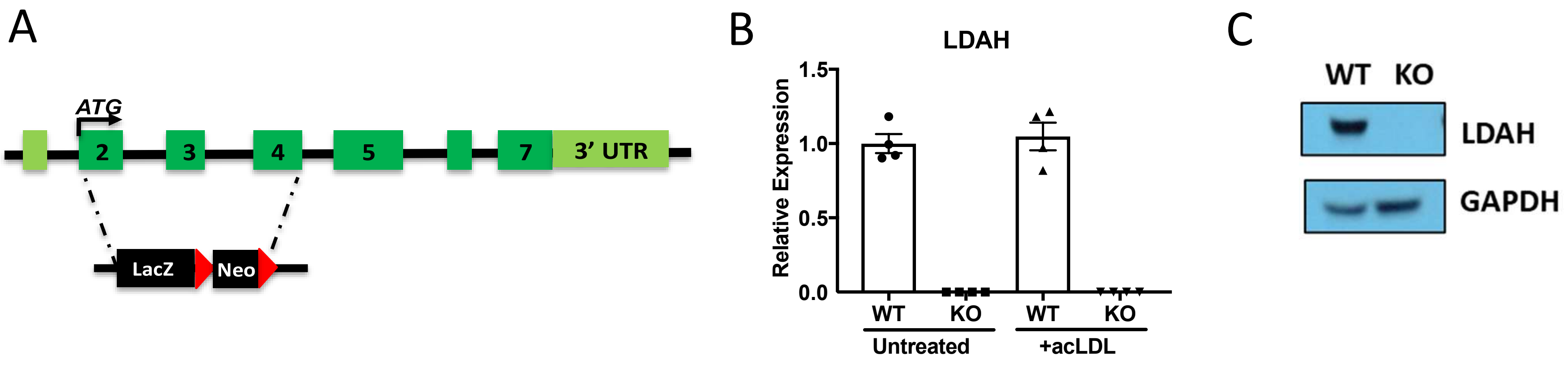


**Figure S3. (A)** LDAH gene targeting strategy by homologous recombination. **(B and C)** LDAH deficiency in peritoneal macrophages isolated from knockout (KO) mice was confirmed by **(B)** qPCR (n=3) and **(C)** Western blot. Data in panel B are presented as mean ± SEM. The blot in panel C is representative of at least 3 independent experiments.

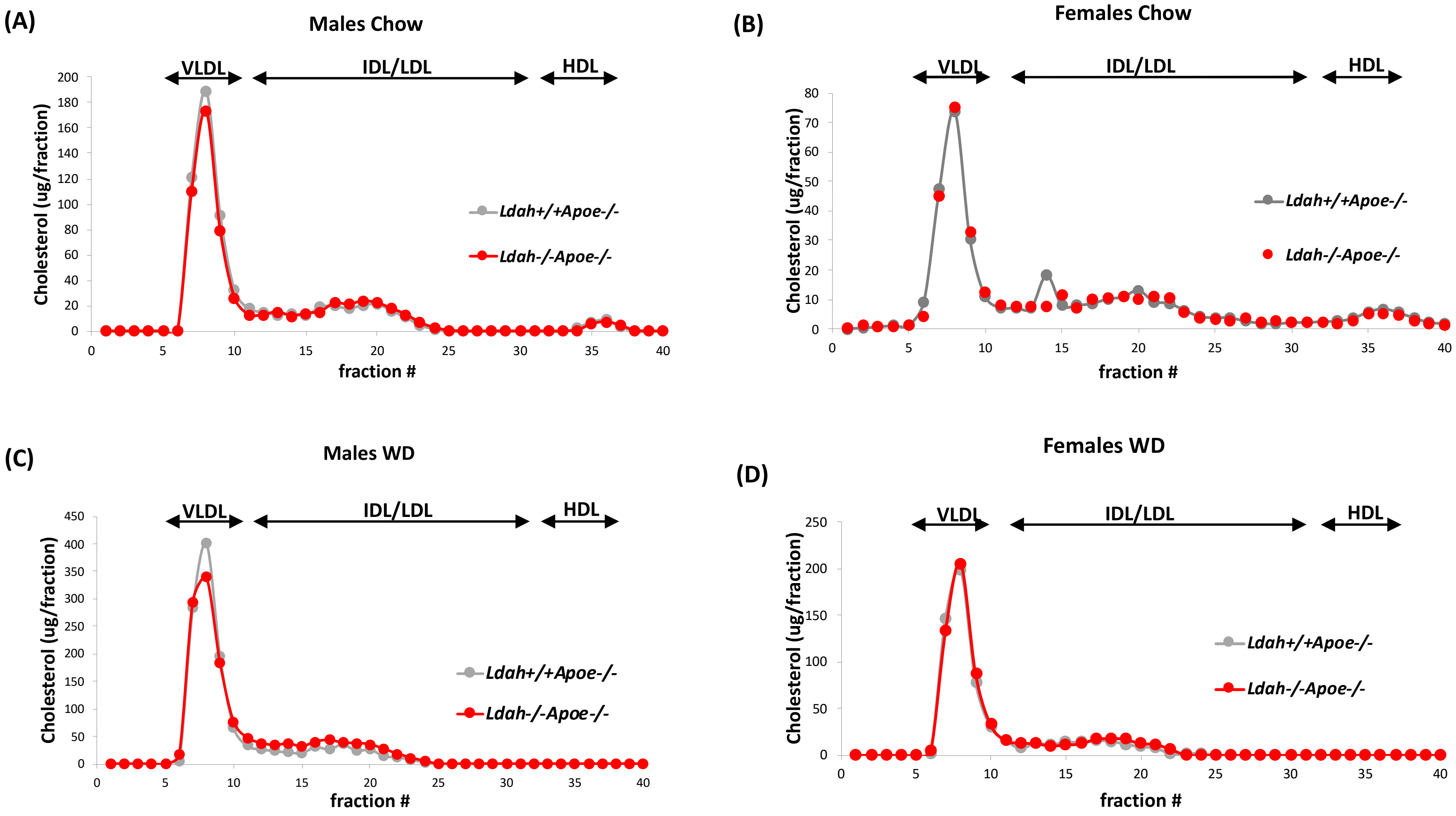


**Figure S4.** Cholesterol distribution in FPLC fractions of pooled plasma in the different experimental groups used to study the effects of LDAH deficiency on atherosclerosis development. **(A)** Males fed regular chow. **(B)** Females fed regular chow. **(C)** Males fed western diet (WD). **(D)** Females fed WD. VLDL: very low-density lipoprotein; IDL/LDL: intermediate-density lipoprotein/low-density lipoprotein; HDL: high-density lipoprotein.

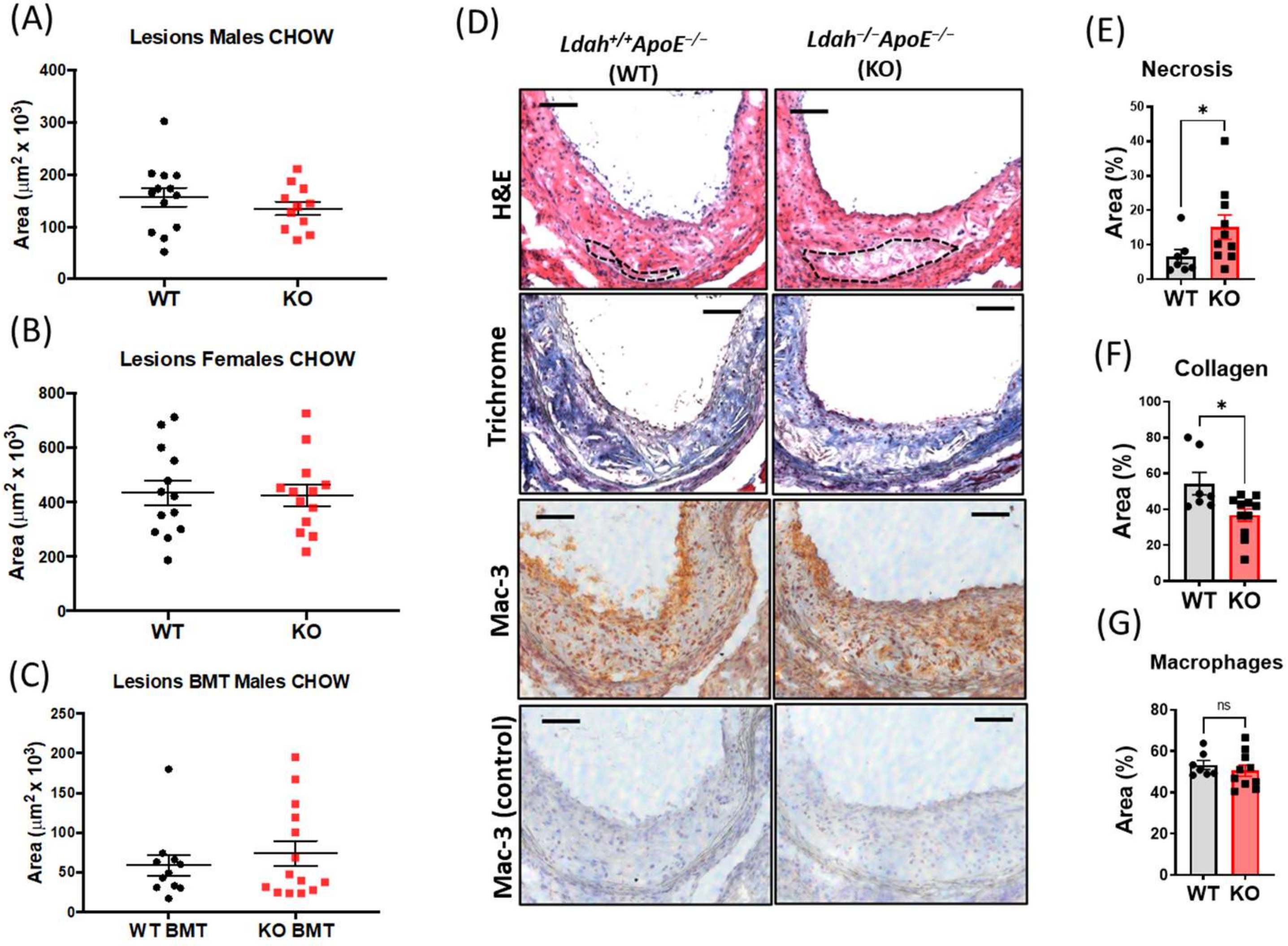


**Figure S5. Effects of LDAH deficiency on atherosclerosis development in mice fed standard mouse chow. (A-C)** In mice fed standard chow global or bone-marrow specific LDAH deficiency did not affect atherosclerosis development. **(A)** LDAH$^{+/+}$/apoE$^{-/-}$ (wild-type, WT, black symbols, n=13) *vs.* global LDAH$^{-/-}$/apoE$^{-/-}$ (KO, red symbols) males (n=11). **(B)** LDAH$^{+/+}$/apoE$^{-/-}$ (wild-type, WT, black symbols, n=13) *vs.* global LDAH$^{-/-}$/apoE$^{-/-}$ (KO, red symbols) females (n=13). **(C)** ApoE$^{-/-}$ male mice were transplanted with bone-marrow cells isolated from LDAH$^{+/+}$/apoE$^{-/-}$ mice (WT BMT, black symbols, n=11 recipients) or from LDAH$^{-/-}$/apoE$^{-/-}$ mice (KO BMT, red symbols, n=14 recipients). (**D)** Representative H&E, Mason's trichrome and Mac-3 images of lesions of female mice. Necrotic cores are outline in black in H&E images. For Mac-3 (control) sections were staining following the Mac-3 staining protocol, without incubation with Mac-3 antibody. Scale bar: 100 µm. **(E)** Quantification of necrotic cores (n= 7 WT and n= 10 KO, *p= 0.033), **(F)** Quantification of collagen (n=7 WT and n=11 KO, *p=0.0441), (**G**) Quantification of % of lesion positive for Mac-3 (n=7 WT and n=10 KO, n.s.= not significant). Grey bars in **E-G** correspond to WT mice and red bars to KO mice. Data are presented as mean ± SEM from independent samples. Panels **C, E** and **F** were analyzed by Mann-Whitney U. Panels **A, B** and **G** were analyzed using two-tailed unpaired *t*-test.

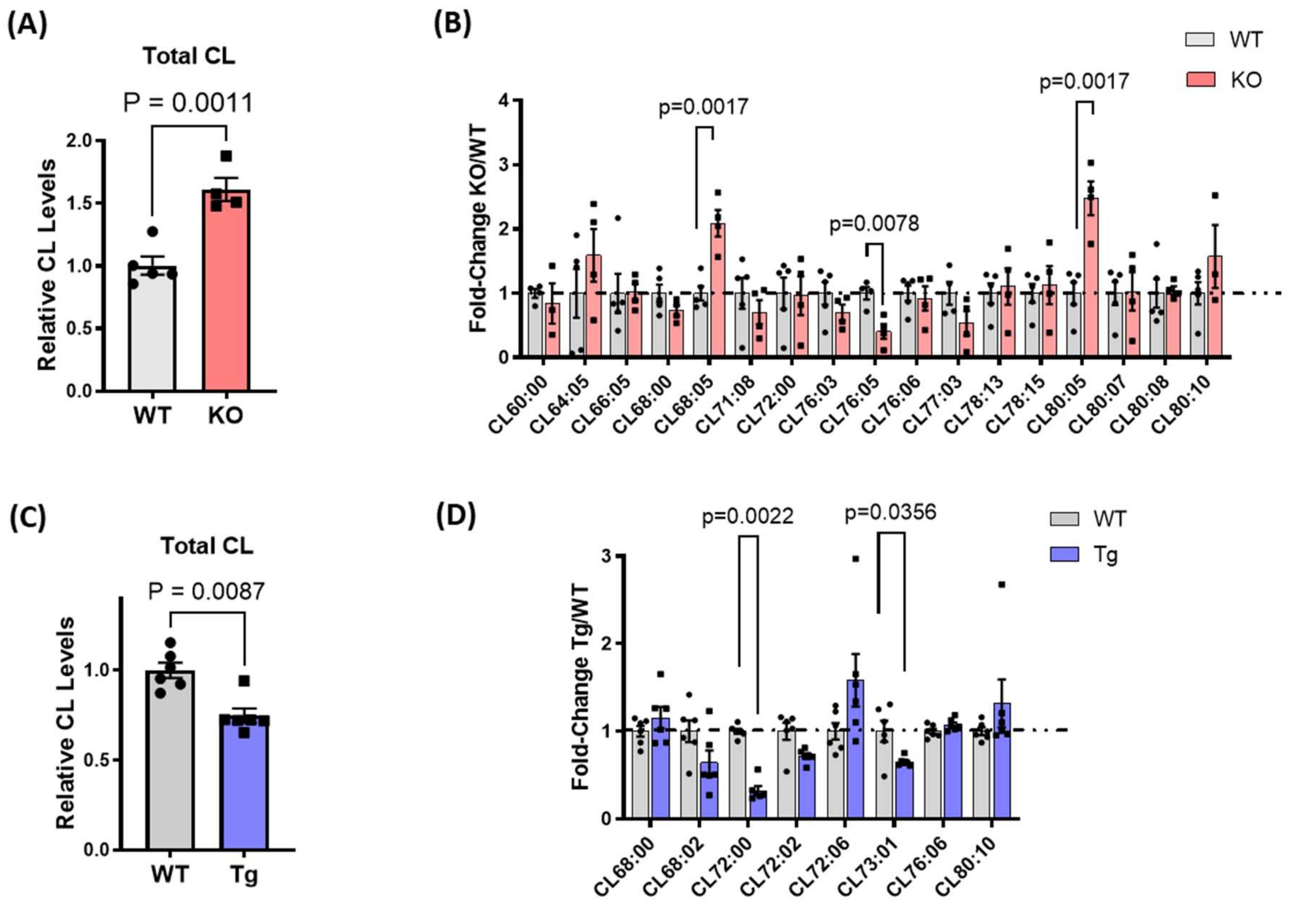


**Figure S6. Impact of LDAH deficiency and overexpression on total cardiolipin (CL) levels and on individual CL species**. **(A and B)** Relative levels of total cardiolipin and individual cardiolipin species in wild-type (WT, grey bars) and *Ldah*-KO (KO, red bars) peritoneal macrophages (PM) treated with oxLDL (50 µg/ml) for 48h. **(A)** Total cardiolipin; **(B)** Main CL species identified by shotgun lipidomics. Bars represent mean ± SEM of n=5 wild-type (WT) and n=4 *Ldah*-KO (KO) independent samples. **(C and D)** Relative levels of total cardiolipin and individual cardiolipin species in wild-type (WT, grey bars) and *Ldah*-Tg (Tg, blue bars) PM treated with oxLDL (50 µg/ml) for 48h. **(A)** Total cardiolipin; **(B)** Main CL species identified by shotgun lipidomics. Bars represent mean ± SEM of n=6 wild-type (WT) and n=6 *Ldah*-Tg (Tg) independent samples. Statistical analyses were performed by two-tailed unpaired *t*-test (CL 68:05, CL76:05, CL 80:05 in WT *vs.* KO), Welch's *t*-test (CL 73:01 in WT *vs.* Tg) or Mann-Whitney U (CL 72:00 in WT *vs.* Tg). The first numbers in the individual CL species shown in the X axis of panels **(B)** and **(D)** represent the sum of the carbon atoms in the four acyl chains of CL, and the numbers behind the colon indicate the total number of double bonds.

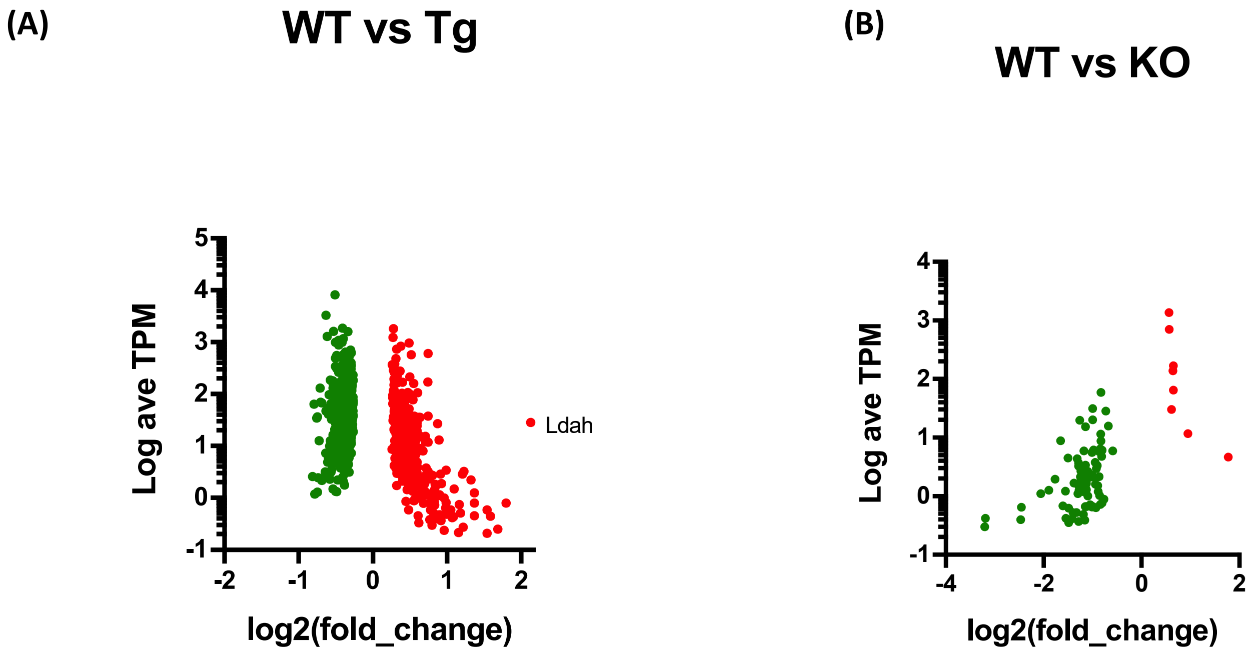


**Figure S7.** Volcano plots summarizing the distribution of of genes differentially expressed at an adjusted p value <0.05 in the RNA-seq analyses . TPM: transcripts per million.

A

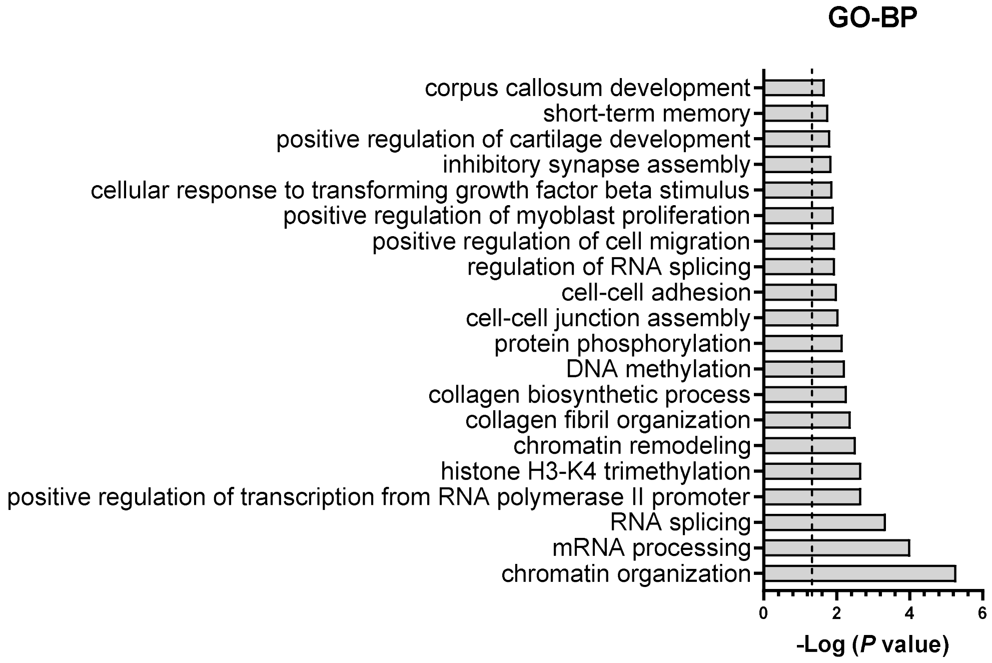


B

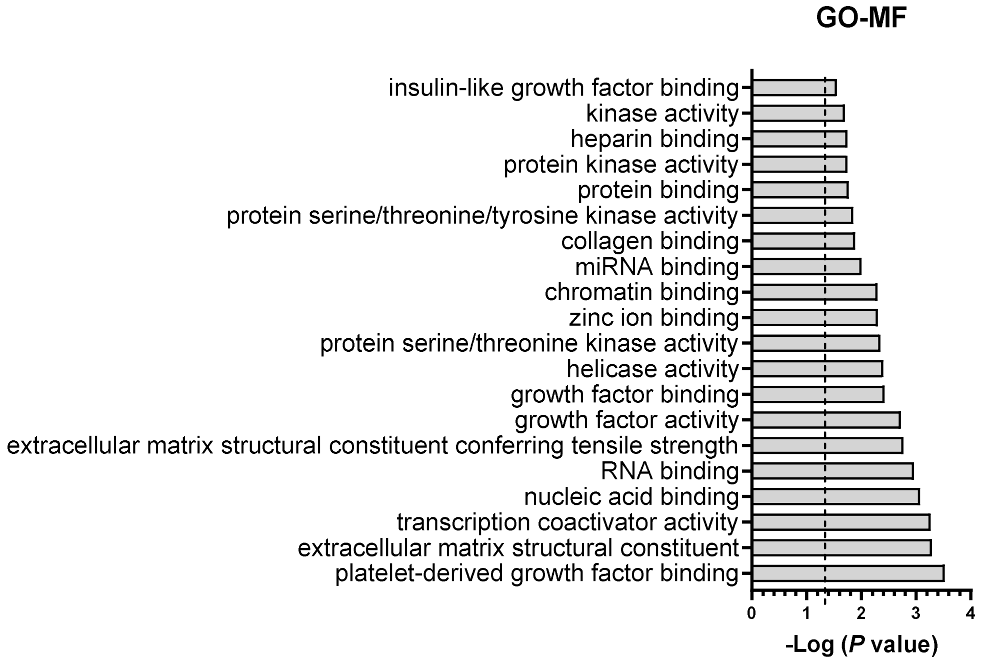


**Fig. S8.** Gene ontology (GO) analysis of genes with expression proportional to LDAH levels identified by RNA-seq was performed using the DAVID Bioinformatics platform. **(A)** GO Biological Process (BP) and **(B)** GO Molecular Function (MF) identified multiple terms related to collagenous extracellular matrix synthesis and organization.

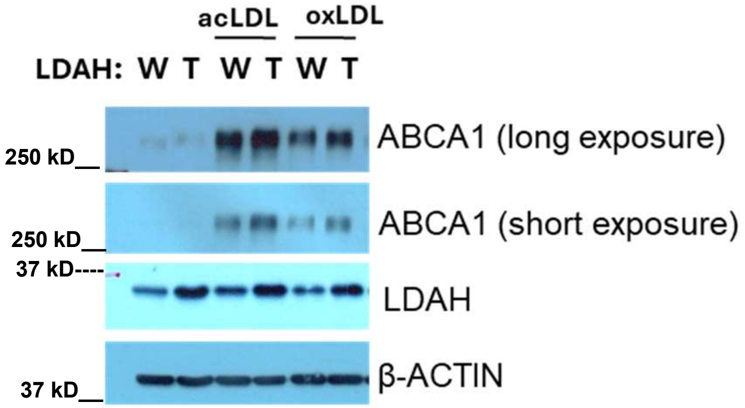


**Fig. S9.** ABCA1 and LDAH expression in wild-type (W) or LDAH-Tg (T) bone marrow macrophages (BMM) that remained untreated or were treated with acLDL (50 µg/mL) or oxLDL (50 µg/mL) for 48h. Blot is representative of 3 independent experiments.

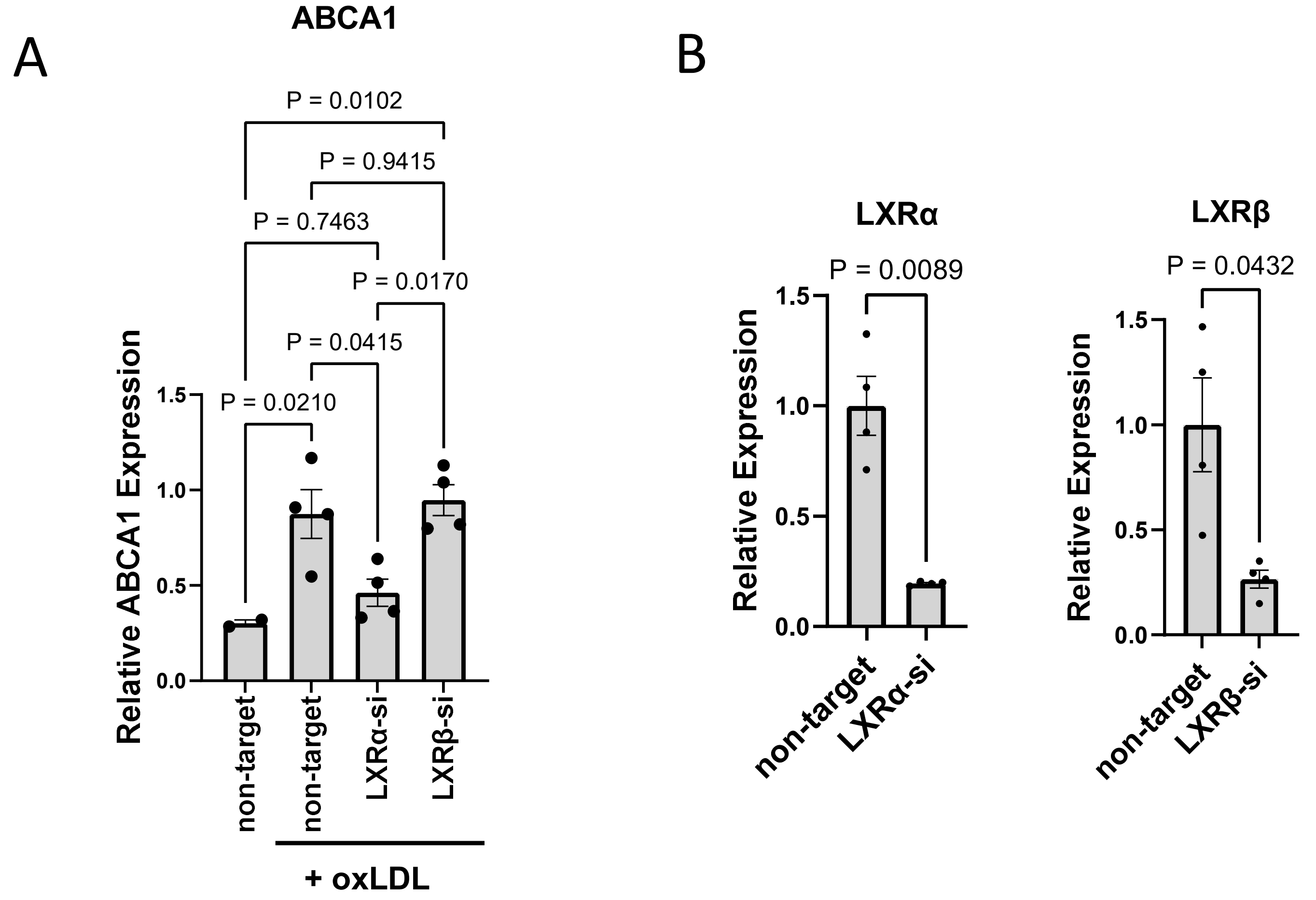


**Fig. S10. (A) )** ABCA1 expression in response to oxLDL (50 µg/mL for 48h) in bone marrow macrophages (BMM) treated with non-target siRNA or RNA against LXRα (LXRα-si) or LXRβ (LXRβ-si). **(B)** LXRα and LXRβ expression in oxLDL-treated BMM used for the experiments in **(A)**. Panel **(A)** was analyzed by one-way ANOVA followed by Tukey's test. Comparisons in panel **(B)** were performed using Welch's *t*-test. N=4/group.

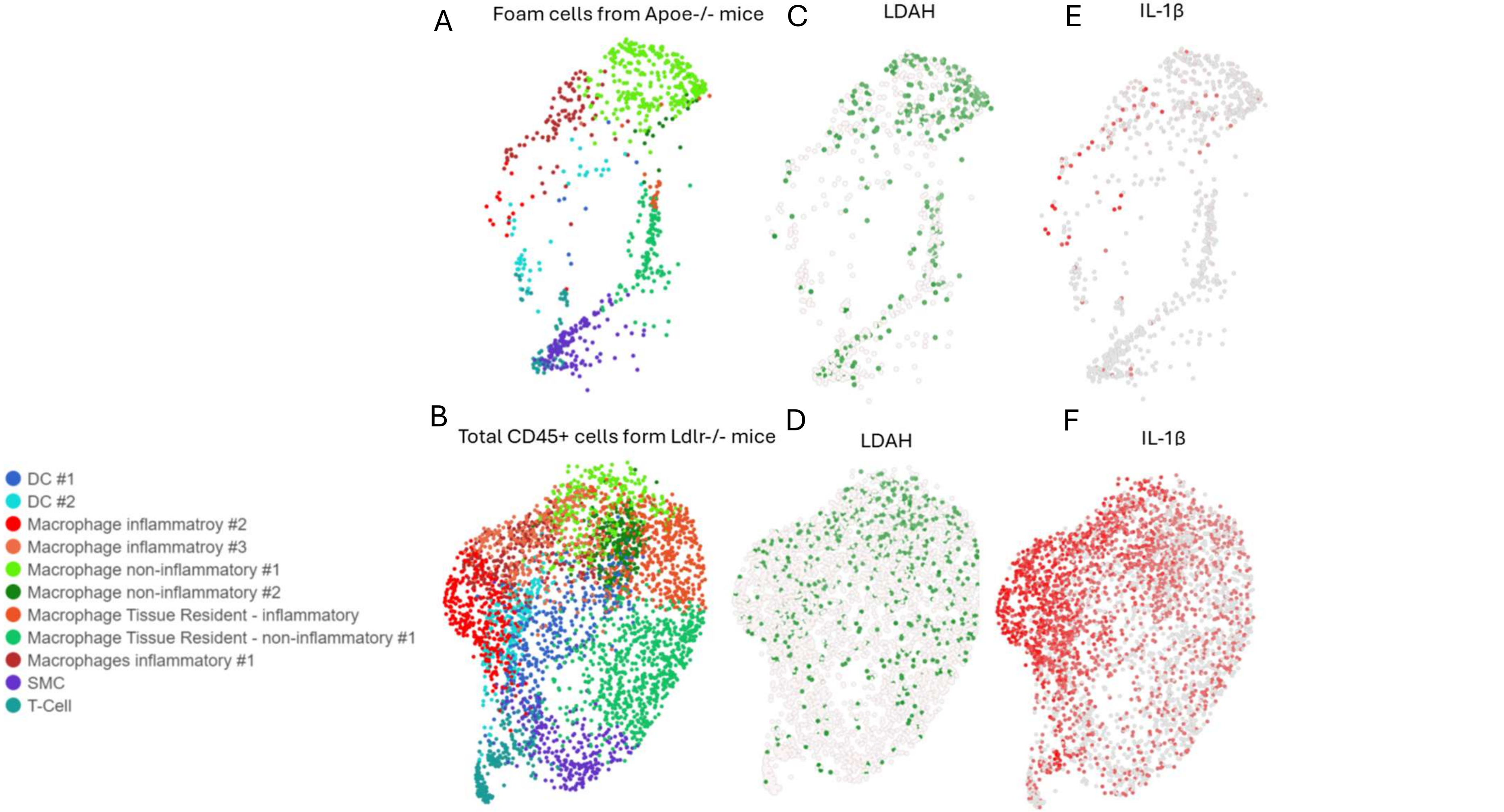


**Fig. S11.** Visualization of total t-SNE clustering and selected genes in the single cell RNA sequencing of aortic CD45+ cells and foam cells from mouse atherosclerotic aortas. **(A-B)** Cluster distribution in foam cells from Apoe-/- mice **(A)** and total CD45+ cells from Ldlr-/- mice **(B)**. **(C-F)** Visualization of LDAH **(C-D)** and IL-1β **(E-F)** in the 2 cell populations distribution in total CD45+ and foam cell populations. Analysis was performed using the single cell sequencing data available in the GEO PRJNA477941 dataset.

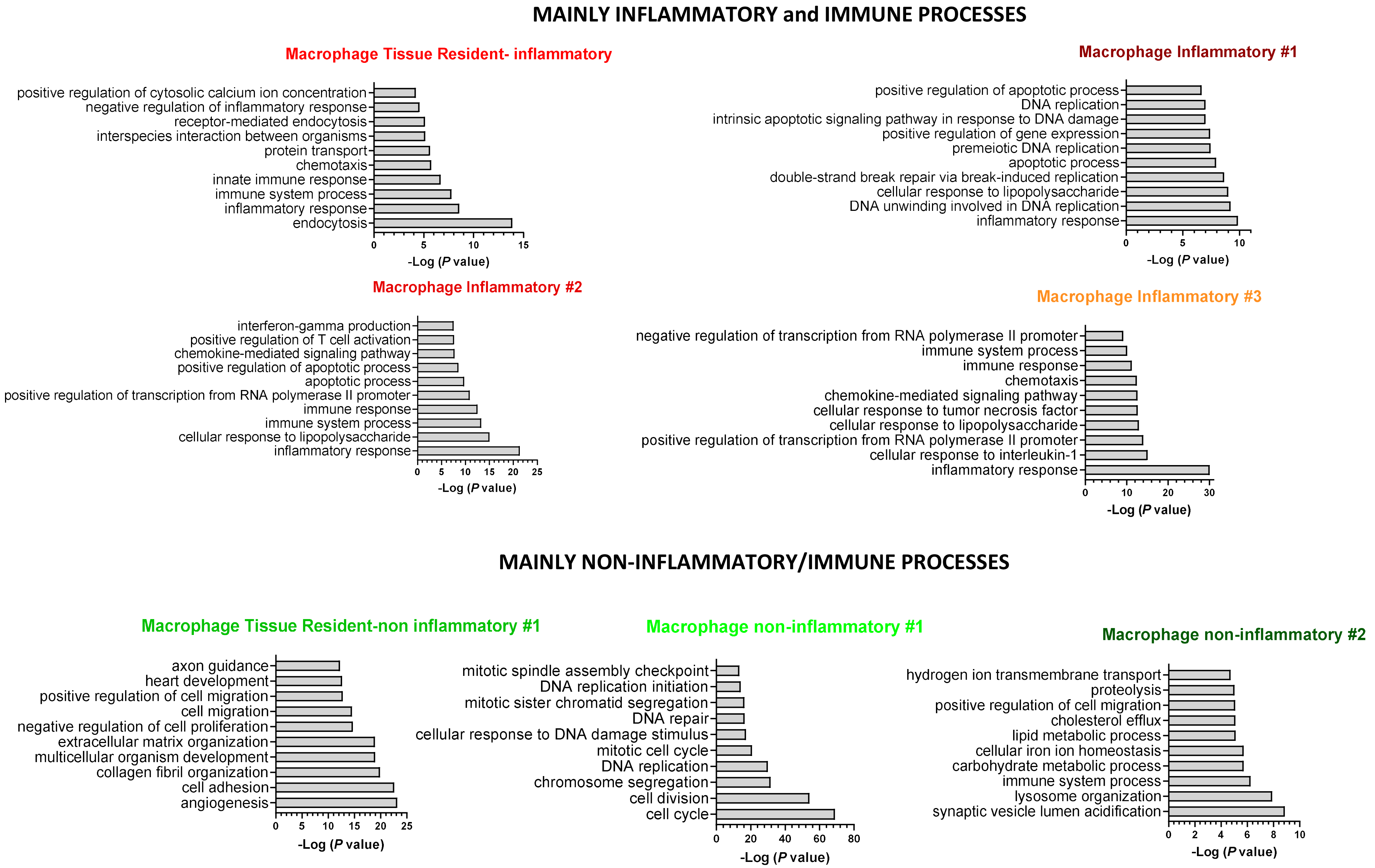


**Fig. S12.** Gene ontology (GO) analysis of genes in the macrophage clusters shown in **Figure S11**. Panels show the top 10 most significant GO Biological Process (BP) outputs. Analyses of macrophage clusters 3, 6, 7 and 8 showed high enrichment in inflammation and immune regulation regulated. Clusters 1, 2 and 11 were mainly enriched in biological processes not directly related to immune and inflammatory processes. Analyses were performed using the DAVID Bioinformatics platform.

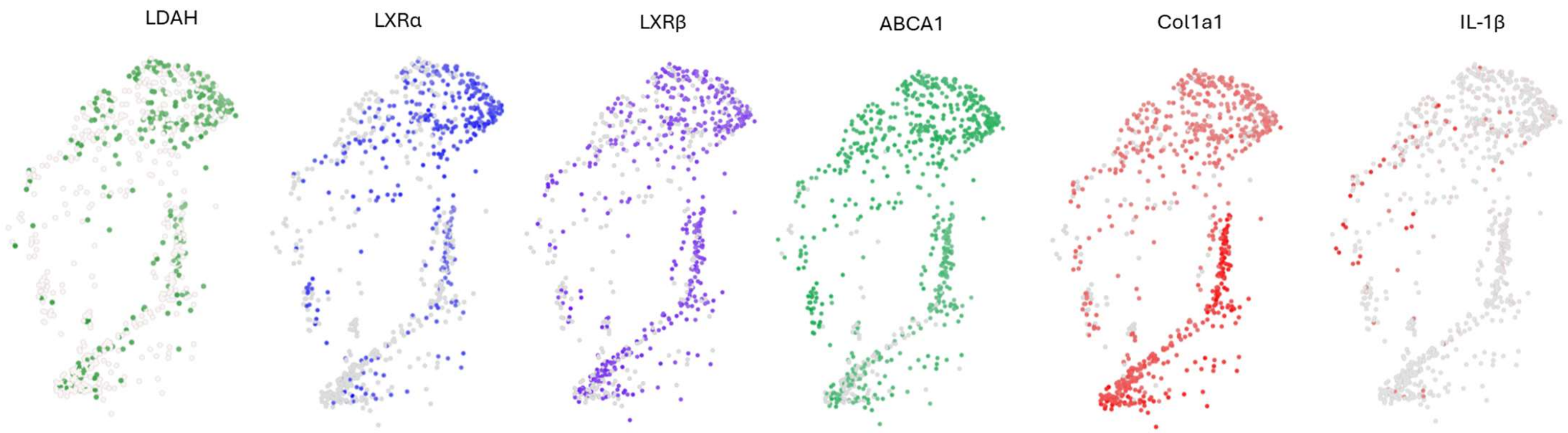


**Fig. S13.** Visualization of total t-SNEs for selected genes in the single cell RNA sequencing of aortic foam cells from Apoe-/- mice. Analysis was performed using the single cell sequencing data available in the GEO PRJNA477941 dataset.

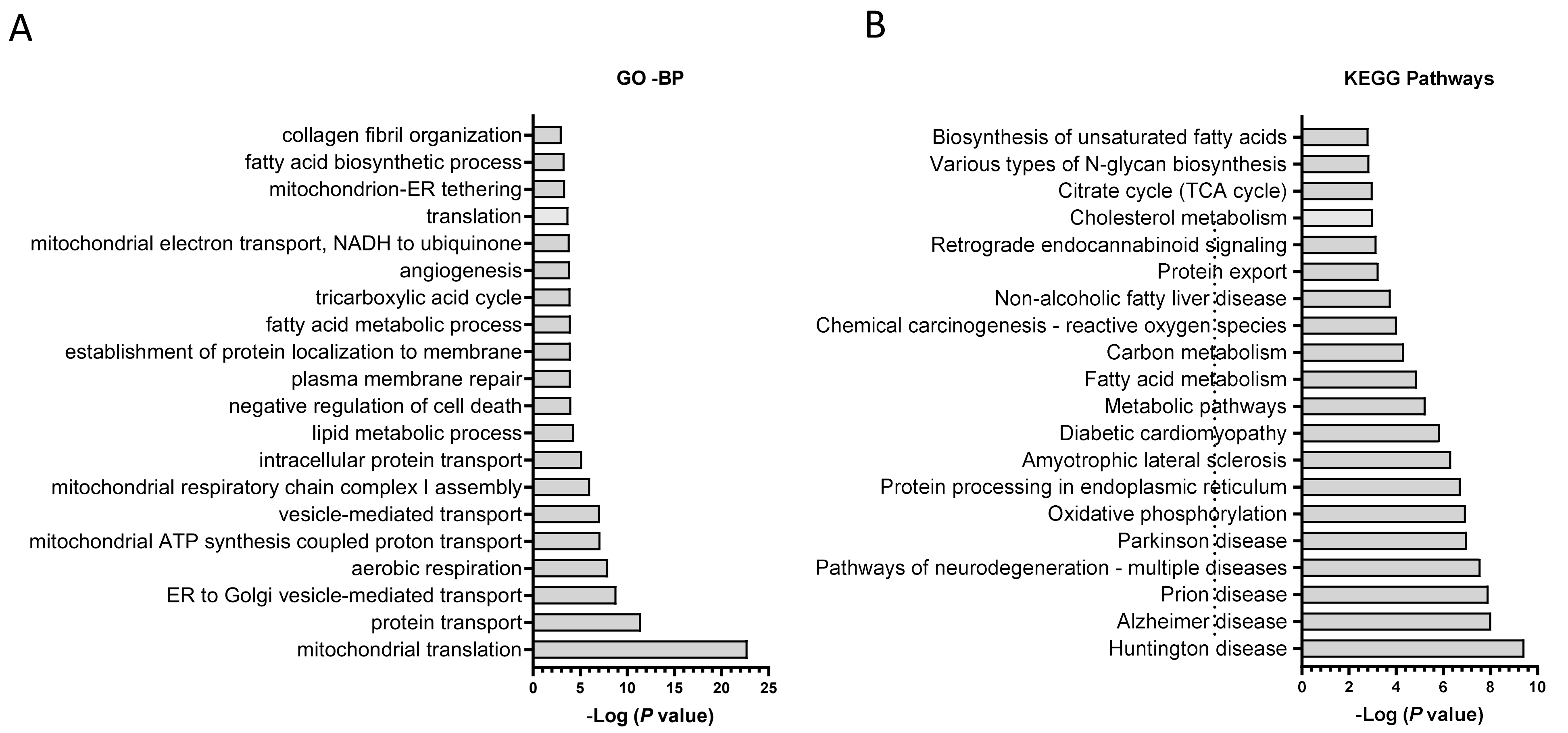


**Fig. S14.** Gene ontology (GO) and pathway analysis of genes that correlate with LDAH at a false discovery rate (FDR) <e-7 in in the single cell RNA sequencing of aortic CD45+ cells and foam cells from mouse atherosclerotic aortas in GSE116240. Panels show the top 20 most significant GO Biological Process (BP) **(A)**) and KEGG pathways **(B)** identified using the DAVID Bioinformatics platform. The analyses returned multiple metabolism and fibrosis-related terms, in the absence of inflammation-related terms.

**Table S1.** Body weights and plasma lipids in male and female *Ldah*$^{0/0}$/*Apoe*$^{-/-}$ (WT) and *Ldah*$^{Tg/0}$/*Apoe*$^{-/-}$ (*Ldah*-Tg) mice fed a standard chow diet.

| | **Chow Diet** | | | |
|---|---|---|---|---|
| | **Males** | | **Females** | |
| | **WT** | ***Ldah*-Tg** | **WT** | ***Ldah*-Tg** |
| **Body weight (g)** | 28.1 ± 0.6 | 27.1 ± 0.6 | 21.0 ± 0.6 | 21.8 ± 0.3 |
| **Triglyceride (mg/dL)** | 112 ± 12 | 126 ± 8 | 76 ±4 | 80 ± 5 |
| **Cholesterol (mg/dL)** | 610 ± 28 | 630 ± 86 | 447 ± 20 | 433 ± 17 |
| **HDL-C (mg/dL)** | 27 ± 3 | 29 ± 2 | 19 ± 4 | 17 ± 2 |

**Table S2.** Body weights and plasma lipids in male and female *Ldah*$^{+/+}$/*Apoe*$^{-/-}$ (WT) and *Ldah*$^{-/-}$/*Apoe*$^{-/-}$ (*Ldah*-KO) mice fed a standard chow diet or western diet (WD).

| | **Chow Diet** | | | |
|---|---|---|---|---|
| | **Males** | | **Females** | |
| | **WT** | ***Ldah*-KO** | **WT** | ***Ldah*-KO** |
| **Body weight (g)** | 28.5 ± 1.0 | 29.2 ± 1.1 | 21.0 ± 0.6 | 22.1 ± 0.4 |
| **Triglyceride (mg/dL)** | 130 ± 13 | 119 ± 14 | 65 ± 5 | 83 ± 8 |
| **Cholesterol (mg/dL)** | 603 ± 31 | 546 ± 32 | 370 ± 21 | 413 ± 18 |
| **HDL-C (mg/dL)** | 28 ± 3 | 28 ± 3 | 14 ± 1 | 17 ± 2 |

| | **Western Diet** | | | |
|---|---|---|---|---|
| | **Males** | | **Females** | |
| | **WT** | ***Ldah*-KO** | **WT** | ***Ldah*-KO** |
| **Body weight (g)** | 35.4 ± 1.3 | 35.2 ± 1.4 | 27.6 ± 1.3 | 26.3 ± 1.3 |
| **Triglyceride (mg/dL)** | 125 ± 8 | 115 ± 10 | 101 ± 5 | 95 ± 9 |
| **Cholesterol (mg/dL)** | 1674 ± 61 | 1697 ± 65 | 960 ± 44 | 1011 ± 47 |
| **HDL-C (mg/dL)** | 20 ± 3 | 20 ± 2 | 17 ± 1 | 17 ± 1 |

**Table S3. Main lipid classess and subclassess in *Ldah* -KO (K) vs. wild-type (WT) peritoneal macrophages treated wit oxLDL (50 µg/ml). Data are presented as normalized ion abundance per microgram of protein.**

| **Lipid Category** | **W1** | **W2** | **W3** | **W4** | **W5** | **AVG** | **SEM** | **K1** | **K2** | **K3** | **K4** | **AVG** | **SEM** |
|---|---|---|---|---|---|---|---|---|---|---|---|---|---|
| Total Chol Esters | 4.077 | 3.477 | 3.392 | 3.860 | 2.931 | 3.547 | 0.198 | 4.651 | 4.475 | 4.074 | 4.508 | 4.427 | 0.124 |
| Chol | 1.025 | 1.105 | 1.021 | 1.112 | 0.869 | 1.027 | 0.044 | 1.385 | 1.293 | 1.237 | 1.268 | 1.296 | 0.032 |
| **Total Sterols** | **5.102** | **4.582** | **4.414** | **4.972** | **3.800** | **4.574** | **0.231** | **6.036** | **5.768** | **5.311** | **5.777** | **5.723** | **0.151** |
| Total MG | N.D. | 0.037 | 0.033 | 0.038 | 0.035 | 0.036 | 0.001 | 0.044 | 0.034 | 0.029 | 0.050 | 0.039 | 0.005 |
| Total DG | 0.327 | 0.174 | 0.172 | 0.197 | 0.153 | 0.205 | 0.031 | 0.193 | 0.187 | 0.168 | 0.176 | 0.181 | 0.006 |
| Total TG | 0.519 | 0.512 | 0.496 | 0.574 | 0.448 | 0.510 | 0.130 | 0.494 | 0.451 | 0.388 | 0.507 | 0.460 | 0.027 |
| **Total Neutral Lipids** | **0.846** | **0.724** | **0.701** | **0.810** | **0.636** | **0.751** | **0.163** | **0.732** | **0.671** | **0.585** | **0.733** | **0.680** | **0.037** |
| **NEFA** | **0.422** | **0.523** | **0.532** | **0.523** | **0.523** | **0.505** | **0.021** | **0.812** | **0.544** | **0.563** | **0.566** | **0.621** | **0.064** |
| Total Lyso PC | N.D. | 0.300 | 0.295 | 0.336 | 0.260 | 0.298 | 0.014 | 0.518 | 0.528 | 0.485 | 0.435 | 0.491 | 0.021 |
| Total Diacyl PC | 17.243 | 15.893 | 15.837 | 18.079 | 13.650 | 16.140 | 0.753 | 25.596 | 23.849 | 21.823 | 22.267 | 23.384 | 0.856 |
| Total ePC | 1.390 | 1.592 | 1.602 | 1.828 | 1.389 | 1.560 | 0.081 | 2.672 | 2.528 | 2.292 | 2.339 | 2.458 | 0.088 |
| Total Ox-PC | 0.047 | 0.014 | 0.032 | 0.029 | 0.022 | 0.029 | 0.006 | 0.060 | 0.108 | 0.088 | 0.079 | 0.084 | 0.010 |
| **Total PC** | **19.102** | **18.322** | **18.298** | **20.794** | **15.845** | **18.472** | **0.798** | **29.657** | **27.557** | **25.252** | **25.686** | **27.038** | **1.006** |
| Total Lyso PE | 0.494 | 0.450 | 0.461 | 0.313 | 0.281 | 0.400 | 0.043 | 0.612 | 0.568 | 0.417 | 0.354 | 0.488 | 0.061 |
| Total Diacyl PE | 13.720 | 13.298 | 13.694 | 8.976 | 8.459 | 11.630 | 1.194 | 15.804 | 15.456 | 11.494 | 9.052 | 12.951 | 1.626 |
| Total pPE | 13.926 | 13.386 | 13.943 | 9.108 | 8.598 | 11.792 | 1.207 | 17.000 | 16.517 | 12.286 | 9.505 | 13.827 | 1.788 |
| **Total PE** | **28.140** | **27.135** | **28.099** | **18.398** | **17.337** | **23.822** | **2.443** | **33.415** | **32.540** | **24.197** | **18.911** | **27.266** | **3.474** |
| Total Lyso PS | 0.044 | 0.051 | 0.035 | 0.031 | 0.030 | 0.038 | 0.004 | 0.096 | 0.056 | 0.033 | 0.038 | 0.056 | 0.014 |
| Total Diacyl PS | 6.258 | 7.117 | 4.552 | 3.580 | 3.454 | 4.992 | 0.730 | 9.031 | 7.642 | 5.195 | 5.030 | 6.724 | 0.974 |
| Total pPS | 0.153 | 0.161 | 0.174 | 0.108 | 0.301 | 0.179 | 0.032 | 0.328 | 0.289 | 0.312 | 0.392 | 0.330 | 0.022 |
| Total ePS | 0.003 | 0.004 | 0.006 | 0.003 | 0.006 | 0.004 | 0.001 | 0.005 | 0.004 | 0.005 | 0.006 | 0.005 | 0.001 |
| **Total PS** | **6.459** | **7.332** | **4.767** | **3.722** | **3.791** | **5.214** | **0.724** | **9.459** | **7.991** | **5.544** | **5.466** | **7.115** | **0.977** |
| Total Lyso PI | 0.060 | 0.062 | 0.070 | 0.053 | 0.047 | 0.058 | 0.004 | 0.094 | 0.089 | 0.077 | 0.050 | 0.078 | 0.010 |
| Total Diacyl PI | 4.455 | 4.299 | 4.262 | 2.973 | 2.361 | 3.670 | 0.422 | 5.168 | 5.026 | 3.621 | 2.631 | 4.112 | 0.605 |
| Total pPI | 0.842 | 0.914 | 0.936 | 0.741 | 0.850 | 0.857 | 0.034 | 1.392 | 1.136 | 1.168 | 0.989 | 1.171 | 0.083 |
| **Total PI** | **5.357** | **5.275** | **5.268** | **3.767** | **3.258** | **4.585** | **0.446** | **6.655** | **6.251** | **4.866** | **3.670** | **5.360** | **0.681** |
| Total Lyso PG | 0.067 | 0.063 | 0.063 | 0.040 | 0.030 | 0.053 | 0.007 | 0.073 | 0.074 | 0.057 | 0.037 | 0.060 | 0.009 |
| Total Diacyl PG | 6.021 | 5.950 | 5.993 | 3.873 | 3.802 | 5.128 | 0.527 | 7.674 | 6.926 | 5.292 | 4.242 | 6.033 | 0.777 |
| Total pPG | 0.017 | 0.014 | 0.015 | 0.016 | 0.002 | 0.013 | 0.003 | 0.017 | 0.016 | 0.009 | 0.002 | 0.011 | 0.004 |
| **Total ALL PG** | **6.105** | **6.027** | **6.071** | **3.929** | **3.834** | **5.193** | **0.536** | **7.764** | **7.016** | **5.357** | **4.281** | **6.104** | **0.789** |
| **Total CL** | **0.594** | **0.755** | **0.557** | **0.554** | **0.508** | **0.594** | **0.043** | **1.112** | **0.876** | **0.933** | **0.893** | **0.953** | **0.054** |
| **Total PA** | **0.506** | **0.498** | **0.411** | **0.326** | **0.127** | **0.373** | **0.070** | **0.654** | **0.544** | **0.326** | **0.133** | **0.414** | **0.116** |
| **TOTAL ALL PHOSPHOLIPIDS** | **66.263** | **65.344** | **63.470** | **51.490** | **44.700** | **58.253** | **4.308** | **88.717** | **82.775** | **66.474** | **59.041** | **74.252** | **6.915** |
| SM | 5.081 | 3.923 | 3.813 | 4.290 | 3.227 | 4.067 | 0.306 | 6.579 | 6.546 | 6.207 | 5.817 | 6.287 | 0.178 |
| Cer | 1.103 | 1.119 | 1.141 | 0.927 | 1.004 | 1.059 | 0.041 | 1.349 | 1.432 | 1.358 | 1.192 | 1.333 | 0.051 |
| 2-hydroxy Cer | 0.006 | 0.002 | 0.012 | 0.007 | 0.005 | 0.007 | 0.002 | 0.001 | 0.004 | 0.004 | 0.003 | 0.003 | 0.001 |
| Hex-Cer | 0.063 | 0.057 | 0.057 | 0.057 | 0.048 | 0.056 | 0.002 | 0.067 | 0.067 | 0.059 | 0.071 | 0.066 | 0.003 |
| Lac-Cer | N.D. | 0.002 | 0.002 | 0.002 | 0.004 | 0.003 | 0.000 | 0.010 | 0.010 | 0.009 | 0.006 | 0.009 | 0.001 |
| **Total All Sphingolipids** | **6.253** | **5.104** | **5.025** | **5.283** | **4.288** | **5.191** | **0.315** | **8.006** | **8.058** | **7.637** | **7.089** | **7.698** | **0.224** |

**Table S4. Main lipid classess and subclassess in *Ldah* -Tg (T) *vs.* wild-type (WT) peritoneal macrophages treated wit oxLDL (50 µg/ml).**
**Data are presented as normalized ion abundance per microgram of protein.**

| Lipid Category | WT1 | WT2 | WT3 | WT4 | WT5 | WT6 | AVG | SEM | T1 | T2 | T3 | T4 | T5 | T6 | AVG | SEM |
|---|---|---|---|---|---|---|---|---|---|---|---|---|---|---|---|---|
| Total Chol Esters | 3.007 | 2.998 | 2.584 | 2.834 | 2.609 | 2.343 | 2.729 | 0.107 | 1.850 | 1.874 | 1.850 | 1.864 | 1.890 | 1.728 | 1.843 | 0.024 |
| Chol | 1.978 | 1.974 | 1.718 | 1.871 | 1.738 | 1.594 | 1.812 | 0.063 | 1.301 | 1.302 | 1.321 | 1.309 | 1.348 | 1.216 | 1.300 | 0.018 |
| **Total Sterols** | **4.985** | **4.972** | **4.303** | **4.705** | **4.347** | **3.937** | **4.541** | **0.170** | **3.150** | **3.176** | **3.172** | **3.174** | **3.238** | **2.945** | **3.142** | **0.041** |
| Total MG | 0.003 | 0.003 | 0.002 | 0.003 | 0.002 | 0.002 | 0.002 | 0.000 | 0.001 | 0.001 | 0.002 | 0.001 | 0.002 | 0.001 | 0.001 | 0.000 |
| Total DG | 0.182 | 0.181 | 0.159 | 0.167 | 0.155 | 0.153 | 0.166 | 0.005 | 0.115 | 0.119 | 0.115 | 0.115 | 0.113 | 0.103 | 0.113 | 0.002 |
| Total TG | 0.651 | 0.612 | 0.531 | 0.568 | 0.528 | 0.517 | 0.568 | 0.022 | 0.480 | 0.447 | 0.451 | 0.454 | 0.466 | 0.431 | 0.455 | 0.007 |
| **Total Neutral Lipids** | **0.835** | **0.795** | **0.692** | **0.738** | **0.684** | **0.673** | **0.736** | **0.027** | **0.596** | **0.568** | **0.567** | **0.571** | **0.581** | **0.534** | **0.569** | **0.008** |
| **Total NEFA** | **0.755** | **0.795** | **0.643** | **0.764** | **0.715** | **0.578** | **0.708** | **0.034** | **0.745** | **0.921** | **0.802** | **0.698** | **0.660** | **0.605** | **0.739** | **0.046** |
| Total Lyso PC | 0.326 | 0.348 | 0.316 | 0.342 | 0.328 | 0.321 | 0.330 | 0.005 | 0.392 | 0.383 | 0.376 | 0.377 | 0.385 | 0.350 | 0.377 | 0.006 |
| Total Diacyl PC | 23.486 | 24.728 | 21.831 | 23.751 | 21.650 | 21.643 | 22.848 | 0.538 | 24.440 | 23.786 | 23.382 | 23.373 | 23.673 | 21.712 | 23.394 | 0.372 |
| Total ePC | 1.969 | 2.041 | 1.824 | 1.957 | 1.819 | 1.849 | 1.910 | 0.038 | 2.220 | 2.128 | 2.089 | 2.087 | 2.117 | 1.943 | 2.098 | 0.037 |
| Total Ox-PC | 0.001 | 0.001 | 0.000 | N.D. | 0.001 | 0.001 | 0.001 | 0.000 | 0.001 | 0.001 | 0.001 | N.D. | 0.001 | 0.001 | 0.001 | 0.000 |
| **Total PC** | **25.783** | **27.118** | **23.972** | **26.050** | **23.798** | **23.813** | **25.089** | **0.579** | **27.054** | **26.298** | **25.849** | **25.837** | **26.175** | **24.005** | **25.870** | **0.415** |
| Total Lyso PE | 0.227 | 0.252 | 0.206 | 0.241 | 0.197 | 0.197 | 0.220 | 0.010 | 0.316 | 0.305 | 0.295 | 0.270 | 0.277 | 0.272 | 0.290 | 0.008 |
| Total Diacyl PE | 6.979 | 7.363 | 6.611 | 6.920 | 6.537 | 6.308 | 6.786 | 0.154 | 7.403 | 7.303 | 7.177 | 7.211 | 7.323 | 6.745 | 7.194 | 0.096 |
| Total pPE | 6.351 | 6.707 | 5.996 | 6.336 | 5.882 | 5.771 | 6.174 | 0.144 | 6.859 | 6.890 | 6.756 | 6.791 | 6.910 | 6.359 | 6.761 | 0.084 |
| **Total PE** | **13.557** | **14.322** | **12.813** | **13.497** | **12.616** | **12.277** | **13.180** | **0.306** | **14.578** | **14.499** | **14.228** | **14.272** | **14.510** | **13.377** | **14.244** | **0.183** |
| Total Lyso PS | 0.030 | 0.033 | 0.026 | 0.031 | 0.023 | 0.025 | 0.028 | 0.002 | 0.053 | 0.047 | 0.045 | 0.042 | 0.042 | 0.042 | 0.045 | 0.002 |
| Total Diacyl PS | 5.079 | 5.377 | 4.741 | 5.063 | 4.705 | 4.540 | 4.917 | 0.126 | 5.324 | 5.229 | 5.071 | 5.188 | 5.308 | 4.898 | 5.170 | 0.066 |
| Total pPS | 0.877 | 0.923 | 0.825 | 0.867 | 0.826 | 0.771 | 0.848 | 0.021 | 0.756 | 0.723 | 0.728 | 0.776 | 0.822 | 0.785 | 0.765 | 0.015 |
| Total ePS | 0.004 | 0.005 | 0.005 | 0.005 | 0.005 | 0.005 | 0.005 | 0.000 | 0.006 | 0.004 | 0.004 | 0.005 | 0.004 | 0.003 | 0.005 | 0.000 |
| **Total ALL PS** | **5.990** | **6.337** | **5.597** | **5.967** | **5.558** | **5.341** | **5.798** | **0.149** | **6.139** | **6.003** | **5.848** | **6.011** | **6.176** | **5.728** | **5.984** | **0.070** |
| Total Lyso PI | 0.024 | 0.026 | 0.022 | 0.026 | 0.019 | 0.022 | 0.023 | 0.001 | 0.049 | 0.048 | 0.045 | 0.042 | 0.045 | 0.043 | 0.045 | 0.001 |
| Total Diacyl PI | 1.578 | 1.689 | 1.415 | 1.606 | 1.407 | 1.392 | 1.514 | 0.051 | 1.897 | 1.894 | 1.781 | 1.821 | 1.833 | 1.708 | 1.822 | 0.029 |
| **Total ALL PI** | **1.602** | **1.715** | **1.437** | **1.632** | **1.426** | **1.413** | **1.538** | **0.053** | **1.947** | **1.942** | **1.826** | **1.863** | **1.877** | **1.751** | **1.867** | **0.030** |
| Total Lyso PG | 0.037 | 0.040 | 0.034 | 0.037 | 0.032 | 0.030 | 0.035 | 0.001 | 0.044 | 0.044 | 0.043 | 0.040 | 0.043 | 0.043 | 0.043 | 0.001 |
| Total Diacyl PG | 1.902 | 2.041 | 1.816 | 1.892 | 1.792 | 1.717 | 1.860 | 0.046 | 2.179 | 2.145 | 2.113 | 2.116 | 2.180 | 2.022 | 2.126 | 0.024 |
| **Total ALL PG** | **1.939** | **2.081** | **1.849** | **1.929** | **1.824** | **1.748** | **1.895** | **0.047** | **2.223** | **2.189** | **2.156** | **2.156** | **2.223** | **2.065** | **2.169** | **0.024** |
| **Total CL** | **0.070** | **0.093** | **0.077** | **0.082** | **0.075** | **0.087** | **0.081** | **0.003** | **0.076** | **0.058** | **0.058** | **0.059** | **0.058** | **0.053** | **0.060** | **0.003** |
| **Total PA** | **0.014** | **0.014** | **0.013** | **0.014** | **0.012** | **0.012** | **0.013** | **0.000** | **0.037** | **0.032** | **0.026** | **0.028** | **0.023** | **0.021** | **0.028** | **0.002** |
| **Total All Phospholipi** | **47.354** | **49.966** | **44.321** | **47.539** | **43.883** | **43.277** | **46.057** | **1.074** | **50.108** | **49.079** | **48.165** | **48.362** | **49.166** | **45.249** | **48.355** | **0.682** |
| SM | 4.654 | 5.172 | 4.497 | 5.014 | 4.485 | 4.335 | 4.693 | 0.135 | 4.926 | 4.974 | 4.910 | 4.823 | 4.898 | 4.431 | 4.827 | 0.082 |
| Cer | 1.103 | 1.119 | 1.123 | 0.927 | 1.004 | 1.004 | 1.047 | 0.033 | 1.349 | 1.432 | 1.209 | 1.358 | 1.192 | 1.192 | 1.289 | 0.043 |
| 2-hydroxy Cer | 0.006 | 0.002 | 0.012 | 0.007 | 0.005 | 0.005 | 0.006 | 0.001 | 0.001 | 0.004 | 0.004 | 0.004 | 0.003 | 0.003 | 0.003 | 0.000 |
| Hex-Cer | 0.018 | 0.023 | 0.020 | 0.025 | 0.019 | 0.023 | 0.021 | 0.001 | 0.027 | 0.030 | 0.028 | 0.027 | 0.026 | 0.023 | 0.027 | 0.001 |
| Lac-Cer | 0.005 | 0.006 | 0.005 | 0.005 | 0.005 | 0.004 | 0.005 | 0.000 | 0.005 | 0.005 | 0.006 | 0.005 | 0.007 | 0.005 | 0.005 | 0.000 |
| **Total All Sphingolipic** | **5.786** | **6.322** | **5.657** | **5.977** | **5.518** | **5.371** | **5.772** | **0.139** | **6.308** | **6.446** | **6.156** | **6.216** | **6.125** | **5.655** | **6.151** | **0.110** |

**Table S5. Genes significantly regulated at and adjusted p-value (q-value)<0.05 in *Ldah* -Tg PM treated with oxLDL.**

Data were analyzed using the Wald test (two-tailed) followed by the Benjamini–Hochberg test to estimate the false discovery rate adjusted p-value (q_value).

| gene_id | gene synonim | log2_fold change_Tg/WT | p_value | q_value |
|---|---|---|---|---|
| ENSMUSG00000037669.14 | Ldah | 2.12724 | 0.00005 | 0.00258735 |
| ENSMUSG00000087445.1 | Gm14286 | 1.79927 | 0.0024 | 0.0394646 |
| ENSMUSG00000036928.14 | Stag3 | 1.68916 | 0.0017 | 0.0314636 |
| ENSMUSG00000021268.17 | Meg3 | 1.59044 | 0.0007 | 0.0179285 |
| ENSMUSG00000026955.13 | Sapcd2 | 1.54334 | 0.0032 | 0.0475054 |
| ENSMUSG00000095040.2 | 1700001J03Rik | 1.53904 | 0.00315 | 0.0472053 |
| ENSMUSG00000028139.5 | Riiad1 | 1.37459 | 0.0003 | 0.0100619 |
| ENSMUSG00000070891.2 | Gm12689 | 1.37459 | 0.00005 | 0.00258735 |
| ENSMUSG00000019737.14 | Syne4 | 1.37174 | 0.00045 | 0.0132986 |
| ENSMUSG00000105008.1 | Gm43652 | 1.32592 | 0.00005 | 0.00258735 |
| ENSMUSG00000031098.18 | Syt8 | 1.23291 | 0.001 | 0.0226798 |
| ENSMUSG00000046295.13 | Ankle1 | 1.21904 | 0.00285 | 0.0440101 |
| ENSMUSG00000045903.8 | Npas4 | 1.20938 | 0.00005 | 0.00258735 |
| ENSMUSG00000009145.6 | Dqx1 | 1.18532 | 0.00005 | 0.00258735 |
| ENSMUSG00000073418.4 | C4b | 1.17038 | 0.003 | 0.0455497 |
| ENSMUSG00000027314.6 | Dll4 | 1.1588 | 0.0015 | 0.0293926 |
| ENSMUSG00000053964.17 | Lgals4 | 1.09883 | 0.00005 | 0.00258735 |
| ENSMUSG00000103115.1 | Gm37584 | 1.09089 | 0.0025 | 0.0405307 |
| ENSMUSG00000048856.8 | Slc25a47 | 1.07317 | 0.00155 | 0.0298193 |
| ENSMUSG00000097534.3 | Gm16675 | 1.05249 | 0.0008 | 0.0195422 |
| ENSMUSG00000021815.3 | Mss51 | 0.996082 | 0.0009 | 0.0212086 |
| ENSMUSG00000106547.1 | B230303O12Rik | 0.988964 | 0.00005 | 0.00258735 |
| ENSMUSG00000080316.10 | Spaca6 | 0.988892 | 0.0026 | 0.0414106 |
| ENSMUSG00000074415.10 | 2610203C20Rik | 0.982298 | 0.00005 | 0.00258735 |
| ENSMUSG00000031861.15 | Lpar2 | 0.965364 | 0.00005 | 0.00258735 |
| ENSMUSG00000028845.15 | Tekt2 | 0.965203 | 0.00095 | 0.0220588 |
| ENSMUSG00000023993.6 | Treml1 | 0.926654 | 0.00005 | 0.00258735 |
| ENSMUSG00000048329.7 | Mfsd6l | 0.922582 | 0.0031 | 0.0466214 |
| ENSMUSG00000099398.1 | Ms4a14 | 0.914776 | 0.00005 | 0.00258735 |
| ENSMUSG00000033763.14 | Mtss1l | 0.909037 | 0.0008 | 0.0195422 |
| ENSMUSG00000074899.4 | Sptbn5 | 0.900573 | 0.00005 | 0.00258735 |
| ENSMUSG00000000386.14 | Mx1 | 0.89838 | 0.0026 | 0.0414106 |
| ENSMUSG00000097451.9 | Rian | 0.898293 | 0.00185 | 0.0333632 |
| ENSMUSG00000096768.7 | Erdr1 | 0.896468 | 0.00005 | 0.00258735 |
| ENSMUSG00000022489.5 | Pde1b | 0.873649 | 0.00005 | 0.00258735 |
| ENSMUSG00000011154.17 | Cfap161 | 0.852316 | 0.0001 | 0.00449574 |
| ENSMUSG00000066975.12 | Cryba4 | 0.848567 | 0.00205 | 0.0356026 |
| ENSMUSG00000023473.11 | Celsr3 | 0.841332 | 0.00075 | 0.0188287 |
| ENSMUSG00000032690.16 | Oas2 | 0.838816 | 0.0001 | 0.00449574 |
| ENSMUSG00000015377.9 | Dennd6b | 0.837011 | 0.00005 | 0.00258735 |
| ENSMUSG00000049676.14 | Catsperg1 | 0.829967 | 0.0029 | 0.0445111 |
| ENSMUSG00000031380.10 | Figf | 0.829391 | 0.00255 | 0.0411309 |
| ENSMUSG00000023912.9 | Slc25a27 | 0.825252 | 0.00205 | 0.0356026 |
| ENSMUSG00000109819.1 | RP24-155I9.3 | 0.824107 | 0.0021 | 0.0362229 |
| ENSMUSG00000054135.2 | A430110L20Rik | 0.815496 | 0.00005 | 0.00258735 |
| ENSMUSG00000027318.17 | Adam33 | 0.806218 | 0.00005 | 0.00258735 |
| ENSMUSG00000002100.15 | Mybpc3 | 0.801451 | 0.00095 | 0.0220588 |
| ENSMUSG00000085028.2 | Slc2a4rg-ps | 0.795623 | 0.00005 | 0.00258735 |
| ENSMUSG00000104943.1 | Gm42868 | 0.781145 | 0.001 | 0.0226798 |
| ENSMUSG00000103839.1 | Gm37607 | 0.777302 | 0.00025 | 0.00905571 |
| ENSMUSG00000045838.8 | A430105I19Rik | 0.770408 | 0.00035 | 0.011149 |
| ENSMUSG00000023795.16 | Pisd-ps2 | 0.763527 | 0.0016 | 0.0303665 |
| ENSMUSG00000097180.5 | 2700038G22Rik | 0.761977 | 0.0001 | 0.00449574 |
| ENSMUSG00000092274.2 | Neat1 | 0.749748 | 0.00005 | 0.00258735 |
| ENSMUSG00000047909.11 | Ankrd16 | 0.748925 | 0.00005 | 0.00258735 |
| ENSMUSG00000051550.10 | Zfp579 | 0.746894 | 0.00005 | 0.00258735 |
| ENSMUSG00000001506.10 | Col1a1 | 0.745774 | 0.00005 | 0.00258735 |
| ENSMUSG00000043833.6 | 2900005J15Rik | 0.745078 | 0.00035 | 0.011149 |
| ENSMUSG00000092341.2 | Malat1 | 0.743202 | 0.00095 | 0.0220588 |
| ENSMUSG00000035299.16 | Mid1 | 0.726302 | 0.0013 | 0.0271076 |
| ENSMUSG00000000753.15 | Serpinf1 | 0.722779 | 0.00345 | 0.0498167 |
| ENSMUSG00000021495.8 | Fam193b | 0.712792 | 0.00005 | 0.00258735 |
| ENSMUSG00000029720.9 | Gm20605 | 0.711353 | 0.00095 | 0.0220588 |

| | | | | |
|---|---|---|---|---|
| ENSMUSG00000096960.1 | A230028O05Rik | 0.704329 | 0.00005 | 0.00258735 |
| ENSMUSG00000032607.12 | Amt | 0.702864 | 0.002 | 0.035364 |
| ENSMUSG00000103983.1 | Gm20045 | 0.683563 | 0.00005 | 0.00258735 |
| ENSMUSG00000045282.7 | Tmem86b | 0.680165 | 0.00185 | 0.0333632 |
| ENSMUSG00000027239.14 | Mdk | 0.680099 | 0.00005 | 0.00258735 |
| ENSMUSG00000106383.1 | E330034L11Rik | 0.679915 | 0.00005 | 0.00258735 |
| ENSMUSG00000004187.10 | Kifc2 | 0.667393 | 0.00005 | 0.00258735 |
| ENSMUSG00000074867.3 | Zfp808 | 0.646855 | 0.00005 | 0.00258735 |
| ENSMUSG00000091144.2 | Phf11c | 0.64659 | 0.0002 | 0.00775413 |
| ENSMUSG00000036856.4 | Wnt4 | 0.642024 | 0.00005 | 0.00258735 |
| ENSMUSG00000018651.14 | Tada2a | 0.64063 | 0.0003 | 0.0100619 |
| ENSMUSG00000097296.1 | Gm26532 | 0.639555 | 0.00005 | 0.00258735 |
| ENSMUSG00000020474.11 | Polm | 0.637051 | 0.00005 | 0.00258735 |
| ENSMUSG00000023007.15 | Prpf40b | 0.637045 | 0.0002 | 0.00775413 |
| ENSMUSG00000101609.1 | Kcnq1ot1 | 0.624514 | 0.00005 | 0.00258735 |
| ENSMUSG00000078485.2 | Plekhn1 | 0.624133 | 0.00005 | 0.00258735 |
| ENSMUSG00000103857.1 | Gm37249 | 0.620179 | 0.0024 | 0.0394646 |
| ENSMUSG00000014786.7 | Slc9a5 | 0.614053 | 0.0015 | 0.0293926 |
| ENSMUSG00000096780.7 | Tmem181b-ps | 0.612651 | 0.00005 | 0.00258735 |
| ENSMUSG00000067203.7 | H2-K2 | 0.611205 | 0.00005 | 0.00258735 |
| ENSMUSG00000022070.6 | Bora | 0.606144 | 0.00005 | 0.00258735 |
| ENSMUSG00000042625.16 | Safb2 | 0.604502 | 0.00025 | 0.00905571 |
| ENSMUSG00000038884.14 | A230050P20Rik | 0.60378 | 0.00005 | 0.00258735 |
| ENSMUSG00000096929.7 | A330023F24Rik | 0.603728 | 0.00005 | 0.00258735 |
| ENSMUSG00000060224.3 | Pyroxd2 | 0.60271 | 0.00005 | 0.00258735 |
| ENSMUSG00000035967.15 | Ddx26b | 0.601304 | 0.0008 | 0.0195422 |
| ENSMUSG00000027522.15 | Stx16 | 0.601015 | 0.00005 | 0.00258735 |
| ENSMUSG00000025507.13 | Pidd1 | 0.596642 | 0.00285 | 0.0440101 |
| ENSMUSG00000086859.3 | Snhg20 | 0.59441 | 0.00015 | 0.00627624 |
| ENSMUSG00000045065.7 | 9930022D16Rik | 0.594076 | 0.00105 | 0.0236027 |
| ENSMUSG00000028028.11 | Alpk1 | 0.592549 | 0.00005 | 0.00258735 |
| ENSMUSG00000033705.16 | Stard9 | 0.590706 | 0.0005 | 0.0142449 |
| ENSMUSG00000035545.13 | Leng8 | 0.587812 | 0.00005 | 0.00258735 |
| ENSMUSG00000071203.6 | Naip5 | 0.587525 | 0.00005 | 0.00258735 |
| ENSMUSG00000046808.17 | Atp10d | 0.583471 | 0.0001 | 0.00449574 |
| ENSMUSG00000044244.18 | Il20rb | 0.580198 | 0.00075 | 0.0188287 |
| ENSMUSG00000040663.8 | Clcf1 | 0.579513 | 0.00005 | 0.00258735 |
| ENSMUSG00000055897.13 | Ppp4r1l-ps | 0.579398 | 0.0008 | 0.0195422 |
| ENSMUSG00000097823.2 | Gm16701 | 0.57861 | 0.00325 | 0.047911 |
| ENSMUSG00000051790.15 | Nlgn2 | 0.57853 | 0.00095 | 0.0220588 |
| ENSMUSG00000073609.9 | D2hgdh | 0.57739 | 0.0006 | 0.0160143 |
| ENSMUSG00000054737.11 | Zfp182 | 0.577255 | 0.00005 | 0.00258735 |
| ENSMUSG00000097313.1 | Gm26569 | 0.57654 | 0.00005 | 0.00258735 |
| ENSMUSG00000042138.7 | Msantd2 | 0.576127 | 0.00005 | 0.00258735 |
| ENSMUSG00000097636.7 | Mirt1 | 0.575583 | 0.0014 | 0.0281287 |
| ENSMUSG00000012017.3 | Scarf2 | 0.571549 | 0.00135 | 0.0276053 |
| ENSMUSG00000086825.1 | Gm15675 | 0.571368 | 0.00055 | 0.0152247 |
| ENSMUSG00000082286.10 | Pisd-ps1 | 0.570852 | 0.00005 | 0.00258735 |
| ENSMUSG00000048537.15 | Phldb1 | 0.570324 | 0.00085 | 0.0203711 |
| ENSMUSG00000083161.1 | Gm11427 | 0.566701 | 0.00005 | 0.00258735 |
| ENSMUSG00000103520.1 | 1110025M09Rik | 0.565141 | 0.00145 | 0.0286788 |
| ENSMUSG00000035649.17 | Zcchc7 | 0.56381 | 0.0001 | 0.00449574 |
| ENSMUSG00000008200.14 | Fnbp4 | 0.559785 | 0.0003 | 0.0100619 |
| ENSMUSG00000098055.1 | Gm26947 | 0.557145 | 0.00295 | 0.0450604 |
| ENSMUSG00000049670.8 | Morn4 | 0.554542 | 0.00175 | 0.0321078 |
| ENSMUSG00000037965.14 | Zc3h7a | 0.55448 | 0.00025 | 0.00905571 |
| ENSMUSG00000032198.8 | Dock6 | 0.552541 | 0.0002 | 0.00775413 |
| ENSMUSG00000037443.13 | Cep85 | 0.547203 | 0.0006 | 0.0160143 |
| ENSMUSG00000085741.8 | 5430405H02Rik | 0.544048 | 0.0006 | 0.0160143 |
| ENSMUSG00000069727.5 | Gm5595 | 0.542098 | 0.0001 | 0.00449574 |
| ENSMUSG00000022974.12 | Paxbp1 | 0.541803 | 0.00225 | 0.038034 |
| ENSMUSG00000101462.2 | Gm3052 | 0.541342 | 0.0028 | 0.0435563 |
| ENSMUSG00000029490.3 | Mfsd7a | 0.540035 | 0.00005 | 0.00258735 |
| ENSMUSG00000024174.8 | Pot1b | 0.539685 | 0.00005 | 0.00258735 |
| ENSMUSG00000034800.15 | Zfp661 | 0.535567 | 0.00005 | 0.00258735 |
| ENSMUSG00000097415.2 | AU020206 | 0.532874 | 0.00005 | 0.00258735 |

| | | | | |
|---|---|---|---|---|
| ENSMUSG00000056592.14 | Zfp658 | 0.531401 | 0.00005 | 0.00258735 |
| ENSMUSG00000099481.6 | Xndc1 | 0.531148 | 0.00035 | 0.011149 |
| ENSMUSG00000037266.18 | Rsrp1 | 0.521973 | 0.0003 | 0.0100619 |
| ENSMUSG00000029524.16 | Sirt4 | 0.520928 | 0.0016 | 0.0303665 |
| ENSMUSG00000032621.8 | Srek1 | 0.51891 | 0.0008 | 0.0195422 |
| ENSMUSG00000030279.15 | C2cd5 | 0.516531 | 0.0002 | 0.00775413 |
| ENSMUSG00000026875.14 | Traf1 | 0.511764 | 0.00105 | 0.0236027 |
| ENSMUSG00000028464.16 | Tpm2 | 0.511366 | 0.0027 | 0.04226 |
| ENSMUSG00000042249.11 | Adrbk2 | 0.509783 | 0.00005 | 0.00258735 |
| ENSMUSG00000054051.6 | Ercc6 | 0.509197 | 0.00005 | 0.00258735 |
| ENSMUSG00000014158.11 | Trpv4 | 0.508717 | 0.00005 | 0.00258735 |
| ENSMUSG00000068394.4 | Cep152 | 0.507763 | 0.00005 | 0.00258735 |
| ENSMUSG00000037243.17 | Zfp692 | 0.507123 | 0.00005 | 0.00258735 |
| ENSMUSG00000032177.16 | Pde4a | 0.50623 | 0.0003 | 0.0100619 |
| ENSMUSG00000024830.16 | Rps6kb2 | 0.505946 | 0.00005 | 0.00258735 |
| ENSMUSG00000093765.7 | Gm20658 | 0.502857 | 0.00005 | 0.00258735 |
| ENSMUSG00000053483.13 | Usp21 | 0.500353 | 0.00055 | 0.0152247 |
| ENSMUSG00000007850.16 | Hnrnph1 | 0.499387 | 0.0003 | 0.0100619 |
| ENSMUSG00000041354.15 | Rgl2 | 0.498781 | 0.0002 | 0.00775413 |
| ENSMUSG00000030432.12 | Rpl28 | 0.492727 | 0.0008 | 0.0195422 |
| ENSMUSG00000005682.8 | Pan2 | 0.491977 | 0.00005 | 0.00258735 |
| ENSMUSG00000024299.16 | Adamts10 | 0.491331 | 0.00265 | 0.0418911 |
| ENSMUSG00000029027.8 | Dffb | 0.491116 | 0.0029 | 0.0445111 |
| ENSMUSG00000004267.16 | Eno2 | 0.490393 | 0.00005 | 0.00258735 |
| ENSMUSG00000083563.1 | Gm13340 | 0.489016 | 0.00005 | 0.00258735 |
| ENSMUSG00000050737.13 | Ptges | 0.488789 | 0.00285 | 0.0440101 |
| ENSMUSG00000066640.11 | Fbxl18 | 0.487948 | 0.0017 | 0.0314636 |
| ENSMUSG00000059851.15 | Suv420h2 | 0.487889 | 0.00025 | 0.00905571 |
| ENSMUSG00000066861.14 | Oas1g | 0.486918 | 0.0016 | 0.0303665 |
| ENSMUSG00000052415.5 | Tchh | 0.486191 | 0.003 | 0.0455497 |
| ENSMUSG00000029823.16 | Luc7l2 | 0.485365 | 0.00005 | 0.00258735 |
| ENSMUSG00000045466.18 | Zfp956 | 0.48265 | 0.00035 | 0.011149 |
| ENSMUSG00000035759.3 | Bbs10 | 0.482107 | 0.00005 | 0.00258735 |
| ENSMUSG00000035713.15 | Usp35 | 0.480921 | 0.00205 | 0.0356026 |
| ENSMUSG00000070390.12 | Nlrp1b | 0.479022 | 0.0002 | 0.00775413 |
| ENSMUSG00000021910.15 | Nisch | 0.476426 | 0.0004 | 0.0121904 |
| ENSMUSG00000055436.18 | Srsf11 | 0.473021 | 0.00005 | 0.00258735 |
| ENSMUSG00000031389.17 | Arhgap4 | 0.471822 | 0.00055 | 0.0152247 |
| ENSMUSG00000010608.15 | Rbm25 | 0.470201 | 0.00165 | 0.0308991 |
| ENSMUSG00000074158.9 | 9830147E19Rik | 0.469309 | 0.00025 | 0.00905571 |
| ENSMUSG00000102813.1 | Gm37795 | 0.469109 | 0.0001 | 0.00449574 |
| ENSMUSG00000061028.7 | Clasrp | 0.468529 | 0.00065 | 0.0170615 |
| ENSMUSG00000044813.15 | Shb | 0.467815 | 0.00225 | 0.038034 |
| ENSMUSG00000046402.10 | Rbp1 | 0.465523 | 0.00155 | 0.0298193 |
| ENSMUSG00000036893.17 | Ehmt1 | 0.465452 | 0.0027 | 0.04226 |
| ENSMUSG00000030815.11 | Phkg2 | 0.465267 | 0.00015 | 0.00627624 |
| ENSMUSG00000058402.14 | Zfp420 | 0.464812 | 0.0007 | 0.0179285 |
| ENSMUSG00000055866.9 | Per2 | 0.464561 | 0.00335 | 0.0488738 |
| ENSMUSG00000059708.12 | Akap17b | 0.462488 | 0.0008 | 0.0195422 |
| ENSMUSG00000037989.15 | Wnk2 | 0.46211 | 0.0006 | 0.0160143 |
| ENSMUSG00000029071.16 | Dvl1 | 0.460179 | 0.00005 | 0.00258735 |
| ENSMUSG00000095123.1 | Gm21781 | 0.458661 | 0.0001 | 0.00449574 |
| ENSMUSG00000011158.7 | Brf1 | 0.457699 | 0.00005 | 0.00258735 |
| ENSMUSG00000033434.15 | Gtpbp6 | 0.455008 | 0.00185 | 0.0333632 |
| ENSMUSG00000004561.14 | Mettl17 | 0.454492 | 0.00145 | 0.0286788 |
| ENSMUSG00000059939.13 | 9430015G10Rik | 0.453997 | 0.00205 | 0.0356026 |
| ENSMUSG00000072915.3 | Gm12258 | 0.453352 | 0.00075 | 0.0188287 |
| ENSMUSG00000024451.8 | Arap3 | 0.449736 | 0.00345 | 0.0498167 |
| ENSMUSG00000002910.10 | Arrdc2 | 0.445131 | 0.00005 | 0.00258735 |
| ENSMUSG00000095440.1 | Fignl2 | 0.444996 | 0.00005 | 0.00258735 |
| ENSMUSG00000089726.2 | Mir17hg | 0.444745 | 0.0001 | 0.00449574 |
| ENSMUSG00000020451.17 | Limk2 | 0.443228 | 0.0014 | 0.0281287 |
| ENSMUSG00000038831.16 | Ralgps1 | 0.442767 | 0.00155 | 0.0298193 |
| ENSMUSG00000034248.7 | Slc25a37 | 0.441384 | 0.00005 | 0.00258735 |
| ENSMUSG00000035597.18 | Prpf39 | 0.441359 | 0.00015 | 0.00627624 |
| ENSMUSG00000025145.13 | Lrrc45 | 0.441074 | 0.0004 | 0.0121904 |

| | | | | |
|---|---|---|---|---|
| ENSMUSG00000029581.14 | Fscn1 | 0.440778 | 0.00285 | 0.0440101 |
| ENSMUSG00000044250.7 | Pced1b | 0.440646 | 0.00035 | 0.011149 |
| ENSMUSG00000020156.16 | Mum1 | 0.438181 | 0.00005 | 0.00258735 |
| ENSMUSG00000002668.7 | Dennd1c | 0.437753 | 0.00005 | 0.00258735 |
| ENSMUSG00000061589.14 | Dot1l | 0.435685 | 0.00005 | 0.00258735 |
| ENSMUSG00000009741.14 | Ubp1 | 0.433344 | 0.00005 | 0.00258735 |
| ENSMUSG00000029060.17 | Mib2 | 0.430117 | 0.00105 | 0.0236027 |
| ENSMUSG00000006395.16 | Hyi | 0.429045 | 0.0005 | 0.0142449 |
| ENSMUSG00000062822.7 | 4833420G17Rik | 0.42756 | 0.00005 | 0.00258735 |
| ENSMUSG00000015291.10 | Gdi1 | 0.427125 | 0.00085 | 0.0203711 |
| ENSMUSG00000070808.4 | Gltscr1 | 0.427068 | 0.00005 | 0.00258735 |
| ENSMUSG00000019763.11 | Rmnd1 | 0.426601 | 0.00145 | 0.0286788 |
| ENSMUSG00000078484.3 | Klhl17 | 0.426501 | 0.0002 | 0.00775413 |
| ENSMUSG00000002409.18 | Dyrk1b | 0.426141 | 0.0003 | 0.0100619 |
| ENSMUSG00000037475.15 | Thoc2 | 0.423424 | 0.0002 | 0.00775413 |
| ENSMUSG00000028327.14 | Stra6l | 0.422783 | 0.00005 | 0.00258735 |
| ENSMUSG00000036931.14 | Nfkbid | 0.421479 | 0.00005 | 0.00258735 |
| ENSMUSG00000022617.14 | Chkb | 0.421004 | 0.00005 | 0.00258735 |
| ENSMUSG00000031166.13 | Wdr13 | 0.420997 | 0.00135 | 0.0276053 |
| ENSMUSG00000091811.2 | Inafm1 | 0.420991 | 0.0004 | 0.0121904 |
| ENSMUSG00000021327.18 | Zkscan3 | 0.419777 | 0.00005 | 0.00258735 |
| ENSMUSG00000039199.5 | Zdhhc1 | 0.418494 | 0.0001 | 0.00449574 |
| ENSMUSG00000025137.15 | Pcyt2 | 0.416582 | 0.00065 | 0.0170615 |
| ENSMUSG00000061898.10 | Rbak | 0.414295 | 0.00055 | 0.0152247 |
| ENSMUSG00000029022.18 | Miip | 0.41385 | 0.00015 | 0.00627624 |
| ENSMUSG00000023952.13 | Gtpbp2 | 0.41292 | 0.00265 | 0.0418911 |
| ENSMUSG00000032013.5 | Trim29 | 0.412118 | 0.00005 | 0.00258735 |
| ENSMUSG00000052512.17 | Nav2 | 0.409736 | 0.0005 | 0.0142449 |
| ENSMUSG00000049295.16 | Zfp219 | 0.407324 | 0.00005 | 0.00258735 |
| ENSMUSG00000013236.16 | Ptprs | 0.406992 | 0.00005 | 0.00258735 |
| ENSMUSG00000022228.13 | Zscan26 | 0.406506 | 0.00005 | 0.00258735 |
| ENSMUSG00000020385.16 | Clk4 | 0.406036 | 0.00055 | 0.0152247 |
| ENSMUSG00000041235.11 | Chd7 | 0.404886 | 0.0018 | 0.0328824 |
| ENSMUSG00000039759.10 | Thap3 | 0.404502 | 0.0015 | 0.0293926 |
| ENSMUSG00000063511.11 | Snrnp70 | 0.404319 | 0.0003 | 0.0100619 |
| ENSMUSG00000020902.12 | Ntn1 | 0.404148 | 0.00065 | 0.0170615 |
| ENSMUSG00000045409.16 | Trim39 | 0.40342 | 0.00005 | 0.00258735 |
| ENSMUSG00000058761.5 | Rnf169 | 0.401999 | 0.0001 | 0.00449574 |
| ENSMUSG00000026630.9 | Batf3 | 0.401924 | 0.0027 | 0.04226 |
| ENSMUSG00000052563.13 | D930048N14Rik | 0.401241 | 0.0003 | 0.0100619 |
| ENSMUSG00000036948.17 | BC037034 | 0.401095 | 0.0003 | 0.0100619 |
| ENSMUSG00000000204.15 | Slfn4 | 0.397493 | 0.00155 | 0.0298193 |
| ENSMUSG00000020770.13 | Unk | 0.397133 | 0.00165 | 0.0308991 |
| ENSMUSG00000078942.10 | Naip6 | 0.396315 | 0.00005 | 0.00258735 |
| ENSMUSG00000022185.18 | Acin1 | 0.395279 | 0.00255 | 0.0411309 |
| ENSMUSG00000047617.11 | BC029214 | 0.395134 | 0.00285 | 0.0440101 |
| ENSMUSG00000001248.14 | Gramd1a | 0.393648 | 0.00005 | 0.00258735 |
| ENSMUSG00000031295.13 | Phka2 | 0.393297 | 0.00005 | 0.00258735 |
| ENSMUSG00000025377.14 | Enthd2 | 0.392105 | 0.00195 | 0.0348199 |
| ENSMUSG00000040943.12 | Tet2 | 0.392027 | 0.0001 | 0.00449574 |
| ENSMUSG00000001173.15 | Ocrl | 0.391173 | 0.0002 | 0.00775413 |
| ENSMUSG00000045639.14 | Zfp629 | 0.390758 | 0.00075 | 0.0188287 |
| ENSMUSG00000033819.15 | Ppp1r16a | 0.390599 | 0.00045 | 0.0132986 |
| ENSMUSG00000075600.2 | Zc3h3 | 0.390223 | 0.00065 | 0.0170615 |
| ENSMUSG00000054648.7 | Zfp869 | 0.389906 | 0.0013 | 0.0271076 |
| ENSMUSG00000029068.16 | Ccnl2 | 0.389895 | 0.00005 | 0.00258735 |
| ENSMUSG00000054199.16 | Gon4l | 0.389149 | 0.0006 | 0.0160143 |
| ENSMUSG00000045411.16 | 2410002F23Rik | 0.388591 | 0.00075 | 0.0188287 |
| ENSMUSG00000044037.15 | Als2cl | 0.388532 | 0.00125 | 0.0265008 |
| ENSMUSG00000063952.15 | Brpf3 | 0.386176 | 0.0027 | 0.04226 |
| ENSMUSG00000085156.7 | Snhg15 | 0.385137 | 0.002 | 0.035364 |
| ENSMUSG00000024457.16 | Trim26 | 0.385111 | 0.00005 | 0.00258735 |
| ENSMUSG00000096687.7 | Mfsd4b4 | 0.382918 | 0.0003 | 0.0100619 |
| ENSMUSG00000030122.12 | Ptms | 0.382396 | 0.00085 | 0.0203711 |
| ENSMUSG00000024137.8 | E4f1 | 0.380453 | 0.00025 | 0.00905571 |
| ENSMUSG00000042677.7 | Zc3h12a | 0.379537 | 0.00075 | 0.0188287 |

| | | | | |
|---|---|---|---|---|
| ENSMUSG00000069135.11 | Fgfr1op | 0.379102 | 0.0003 | 0.0100619 |
| ENSMUSG00000042349.13 | Ikbke | 0.378942 | 0.0013 | 0.0271076 |
| ENSMUSG00000023393.15 | Slc17a9 | 0.374006 | 0.0012 | 0.0256986 |
| ENSMUSG00000041268.16 | Dmxl2 | 0.373039 | 0.00085 | 0.0203711 |
| ENSMUSG00000052031.7 | Tagap1 | 0.372173 | 0.00015 | 0.00627624 |
| ENSMUSG00000072812.4 | Ahnak2 | 0.371072 | 0.00035 | 0.011149 |
| ENSMUSG00000031921.17 | Terf2 | 0.370429 | 0.001 | 0.0226798 |
| ENSMUSG00000025086.12 | Trub1 | 0.370117 | 0.00055 | 0.0152247 |
| ENSMUSG00000023087.15 | Noct | 0.369088 | 0.0025 | 0.0405307 |
| ENSMUSG00000079003.2 | Samd1 | 0.368443 | 0.00005 | 0.00258735 |
| ENSMUSG00000044712.15 | Slc38a6 | 0.367927 | 0.00145 | 0.0286788 |
| ENSMUSG00000032212.9 | Sltm | 0.366975 | 0.00005 | 0.00258735 |
| ENSMUSG00000029389.17 | Ddx55 | 0.364357 | 0.0005 | 0.0142449 |
| ENSMUSG00000027615.14 | Hps3 | 0.360117 | 0.00055 | 0.0152247 |
| ENSMUSG00000024193.7 | Phf1 | 0.359026 | 0.00025 | 0.00905571 |
| ENSMUSG00000034160.13 | Ogt | 0.357576 | 0.0003 | 0.0100619 |
| ENSMUSG00000026349.14 | Ccnt2 | 0.356099 | 0.0008 | 0.0195422 |
| ENSMUSG00000040123.16 | Zmym5 | 0.354782 | 0.0004 | 0.0121904 |
| ENSMUSG00000032519.11 | Slc25a38 | 0.35452 | 0.0026 | 0.0414106 |
| ENSMUSG00000042500.11 | Ago4 | 0.354457 | 0.0002 | 0.00775413 |
| ENSMUSG00000042548.13 | Asxl1 | 0.353287 | 0.0004 | 0.0121904 |
| ENSMUSG00000000538.18 | Tom1l2 | 0.351773 | 0.0014 | 0.0281287 |
| ENSMUSG00000037355.14 | Uvssa | 0.351526 | 0.0005 | 0.0142449 |
| ENSMUSG00000028911.16 | Srsf4 | 0.350223 | 0.0001 | 0.00449574 |
| ENSMUSG00000060301.5 | 2610008E11Rik | 0.348856 | 0.00135 | 0.0276053 |
| ENSMUSG00000033909.17 | Usp36 | 0.347101 | 0.0014 | 0.0281287 |
| ENSMUSG00000042439.12 | Zfp532 | 0.346848 | 0.00155 | 0.0298193 |
| ENSMUSG00000097392.2 | D930016D06Rik | 0.346195 | 0.0009 | 0.0212086 |
| ENSMUSG00000038538.17 | Ubn2 | 0.345111 | 0.00225 | 0.038034 |
| ENSMUSG00000052760.15 | A630001G21Rik | 0.344354 | 0.0006 | 0.0160143 |
| ENSMUSG00000018476.7 | Kdm6b | 0.343623 | 0.00185 | 0.0333632 |
| ENSMUSG00000035051.14 | Dhx57 | 0.343309 | 0.0021 | 0.0362229 |
| ENSMUSG00000048174.2 | Tmem81 | 0.342336 | 0.0026 | 0.0414106 |
| ENSMUSG00000034820.3 | Cpsf7 | 0.342137 | 0.00025 | 0.00905571 |
| ENSMUSG00000022095.9 | Fam160b2 | 0.340725 | 0.0004 | 0.0121904 |
| ENSMUSG00000035697.14 | Hmha1 | 0.340597 | 0.0003 | 0.0100619 |
| ENSMUSG00000000759.14 | Tubgcp3 | 0.340253 | 0.0016 | 0.0303665 |
| ENSMUSG00000089762.3 | Ier5l | 0.340159 | 0.00065 | 0.0170615 |
| ENSMUSG00000031508.14 | Ankrd10 | 0.339988 | 0.0012 | 0.0256986 |
| ENSMUSG00000053754.13 | Chd8 | 0.339261 | 0.0024 | 0.0394646 |
| ENSMUSG00000027466.15 | Rbck1 | 0.338484 | 0.00035 | 0.011149 |
| ENSMUSG00000063849.5 | Ppcdc | 0.338252 | 0.00125 | 0.0265008 |
| ENSMUSG00000031805.17 | Jak3 | 0.335811 | 0.00195 | 0.0348199 |
| ENSMUSG00000046229.9 | Scand1 | 0.335542 | 0.00025 | 0.00905571 |
| ENSMUSG00000086290.8 | Snhg12 | 0.332544 | 0.0027 | 0.04226 |
| ENSMUSG00000016495.12 | Plgrkt | 0.332498 | 0.00065 | 0.0170615 |
| ENSMUSG00000034379.8 | Wdr5b | 0.331539 | 0.0034 | 0.0493194 |
| ENSMUSG00000030811.14 | Fbxl19 | 0.33126 | 0.0022 | 0.0374384 |
| ENSMUSG00000020994.4 | Pnn | 0.330798 | 0.00025 | 0.00905571 |
| ENSMUSG00000026972.15 | Arrdc1 | 0.330302 | 0.0019 | 0.0341193 |
| ENSMUSG00000032643.12 | Fhl3 | 0.32954 | 0.00055 | 0.0152247 |
| ENSMUSG00000060862.9 | Zbtb40 | 0.329323 | 0.00155 | 0.0298193 |
| ENSMUSG00000038351.14 | Sgsm2 | 0.329184 | 0.0032 | 0.0475054 |
| ENSMUSG00000003847.16 | Nfat5 | 0.328952 | 0.00155 | 0.0298193 |
| ENSMUSG00000057649.5 | Brd9 | 0.328915 | 0.00055 | 0.0152247 |
| ENSMUSG00000054716.4 | Zfp771 | 0.328878 | 0.00205 | 0.0356026 |
| ENSMUSG00000021704.7 | Mtx3 | 0.328275 | 0.0013 | 0.0271076 |
| ENSMUSG00000052707.8 | Tnrc6a | 0.327659 | 0.00175 | 0.0321078 |
| ENSMUSG00000031706.7 | Rfx1 | 0.327197 | 0.00115 | 0.0251808 |
| ENSMUSG00000032589.14 | Bsn | 0.325655 | 0.00135 | 0.0276053 |
| ENSMUSG00000022529.11 | Zfp263 | 0.325613 | 0.00035 | 0.011149 |
| ENSMUSG00000034022.7 | Cpsf1 | 0.325192 | 0.0021 | 0.0362229 |
| ENSMUSG00000021820.16 | Camk2g | 0.325047 | 0.00045 | 0.0132986 |
| ENSMUSG00000104960.1 | Snhg8 | 0.324462 | 0.00315 | 0.0472053 |
| ENSMUSG00000025158.7 | Rfng | 0.324318 | 0.0013 | 0.0271076 |
| ENSMUSG00000041642.18 | Kif21b | 0.323781 | 0.0004 | 0.0121904 |

| | | | | |
|---|---|---|---|---|
| ENSMUSG00000045128.8 | Rpl18a | 0.323408 | 0.0007 | 0.0179285 |
| ENSMUSG00000048279.17 | Sacs | 0.323124 | 0.00105 | 0.0236027 |
| ENSMUSG00000031833.9 | Mast3 | 0.320968 | 0.00065 | 0.0170615 |
| ENSMUSG00000068917.12 | Clk2 | 0.320287 | 0.0012 | 0.0256986 |
| ENSMUSG00000022559.7 | Fbxl6 | 0.319618 | 0.0011 | 0.0243808 |
| ENSMUSG00000024045.5 | Akap8 | 0.319536 | 0.00025 | 0.00905571 |
| ENSMUSG00000037221.13 | Mospd3 | 0.31899 | 0.0009 | 0.0212086 |
| ENSMUSG00000020150.13 | Gamt | 0.318412 | 0.0023 | 0.0386217 |
| ENSMUSG00000022568.16 | Scrib | 0.316498 | 0.00195 | 0.0348199 |
| ENSMUSG00000032329.3 | Hmg20a | 0.314712 | 0.0011 | 0.0243808 |
| ENSMUSG00000057551.9 | Zfp317 | 0.314612 | 0.00165 | 0.0308991 |
| ENSMUSG00000040712.16 | Camta2 | 0.314165 | 0.0003 | 0.0100619 |
| ENSMUSG00000054312.6 | Mrps21 | 0.313457 | 0.0006 | 0.0160143 |
| ENSMUSG00000041215.15 | Yeats2 | 0.312743 | 0.0032 | 0.0475054 |
| ENSMUSG00000019987.8 | Arg1 | 0.312581 | 0.00055 | 0.0152247 |
| ENSMUSG00000030604.9 | Zfp626 | 0.310139 | 0.00215 | 0.0368347 |
| ENSMUSG00000027569.15 | Mrgbp | 0.308752 | 0.00115 | 0.0251808 |
| ENSMUSG00000026436.15 | Elk4 | 0.30285 | 0.0026 | 0.0414106 |
| ENSMUSG00000064043.13 | Trerf1 | 0.302754 | 0.00245 | 0.0401306 |
| ENSMUSG00000024188.15 | Luc7l | 0.30202 | 0.00325 | 0.047911 |
| ENSMUSG00000028060.14 | 2810403A07Rik | 0.29978 | 0.0006 | 0.0160143 |
| ENSMUSG00000063286.7 | Gm8995 | 0.299401 | 0.002 | 0.035364 |
| ENSMUSG00000020806.15 | Rhbdf2 | 0.298886 | 0.0022 | 0.0374384 |
| ENSMUSG00000021453.2 | Gadd45g | 0.298356 | 0.0007 | 0.0179285 |
| ENSMUSG00000021819.10 | Zswim8 | 0.298247 | 0.00065 | 0.0170615 |
| ENSMUSG00000013858.14 | Tmem259 | 0.296888 | 0.0013 | 0.0271076 |
| ENSMUSG00000042404.16 | Dennd4b | 0.296484 | 0.0024 | 0.0394646 |
| ENSMUSG00000026544.6 | Dusp23 | 0.29605 | 0.0015 | 0.0293926 |
| ENSMUSG00000035711.4 | Dok3 | 0.295193 | 0.0006 | 0.0160143 |
| ENSMUSG00000035762.10 | Tmem161b | 0.294605 | 0.00305 | 0.0460881 |
| ENSMUSG00000041164.15 | Zmiz2 | 0.294363 | 0.00125 | 0.0265008 |
| ENSMUSG00000049422.6 | Chchd10 | 0.294325 | 0.0012 | 0.0256986 |
| ENSMUSG00000027580.17 | Helz2 | 0.293739 | 0.00335 | 0.0488738 |
| ENSMUSG00000037907.16 | Ankrd13b | 0.29315 | 0.0012 | 0.0256986 |
| ENSMUSG00000033020.6 | Polr2f | 0.292936 | 0.0011 | 0.0243808 |
| ENSMUSG00000001918.17 | Slc1a5 | 0.292262 | 0.002 | 0.035364 |
| ENSMUSG00000002028.12 | Kmt2a | 0.291713 | 0.0009 | 0.0212086 |
| ENSMUSG00000029817.11 | Tra2a | 0.291 | 0.00175 | 0.0321078 |
| ENSMUSG00000001034.17 | Mapk7 | 0.288695 | 0.0023 | 0.0386217 |
| ENSMUSG00000025144.17 | Stra13 | 0.288484 | 0.0017 | 0.0314636 |
| ENSMUSG00000038342.15 | Mlxip | 0.288018 | 0.0022 | 0.0374384 |
| ENSMUSG00000024856.10 | Cdk2ap2 | 0.287504 | 0.00155 | 0.0298193 |
| ENSMUSG00000042675.15 | Ypel3 | 0.283558 | 0.00325 | 0.047911 |
| ENSMUSG00000038406.17 | Scaf1 | 0.282424 | 0.00305 | 0.0460881 |
| ENSMUSG00000067288.12 | Rps28 | 0.282097 | 0.00185 | 0.0333632 |
| ENSMUSG00000090247.7 | Bloc1s1 | 0.279206 | 0.00205 | 0.0356026 |
| ENSMUSG00000033106.5 | Slc7a6os | 0.277417 | 0.0033 | 0.0485353 |
| ENSMUSG00000028936.15 | Rpl22 | 0.275213 | 0.00295 | 0.0450604 |
| ENSMUSG00000034157.12 | Cipc | 0.271187 | 0.0026 | 0.0414106 |
| ENSMUSG00000055065.6 | Ddx17 | 0.270191 | 0.00295 | 0.0450604 |
| ENSMUSG00000068758.7 | Il3ra | 0.269108 | 0.00265 | 0.0418911 |
| ENSMUSG00000031765.7 | Mt1 | 0.267296 | 0.0014 | 0.0281287 |
| ENSMUSG00000022263.9 | Trio | 0.26239 | 0.00285 | 0.0440101 |
| ENSMUSG00000055943.5 | Emc7 | -0.259959 | 0.0034 | 0.0493194 |
| ENSMUSG00000041781.8 | Cpsf2 | -0.260727 | 0.0034 | 0.0493194 |
| ENSMUSG00000021024.14 | Psma6 | -0.261319 | 0.00345 | 0.0498167 |
| ENSMUSG00000033047.4 | Eif3l | -0.261976 | 0.0034 | 0.0493194 |
| ENSMUSG00000021003.9 | Galc | -0.262079 | 0.0027 | 0.04226 |
| ENSMUSG00000041958.10 | Pigs | -0.262644 | 0.00265 | 0.0418911 |
| ENSMUSG00000024679.10 | Ms4a6d | -0.263737 | 0.00275 | 0.0429366 |
| ENSMUSG00000024740.9 | Ddb1 | -0.26391 | 0.0031 | 0.0466214 |
| ENSMUSG00000027775.9 | Mfsd1 | -0.26537 | 0.0029 | 0.0445111 |
| ENSMUSG00000006373.10 | Pgrmc1 | -0.266901 | 0.00235 | 0.0389965 |
| ENSMUSG00000019814.4 | Ltv1 | -0.267139 | 0.00335 | 0.0488738 |
| ENSMUSG00000024981.5 | Acsl5 | -0.267683 | 0.0024 | 0.0394646 |
| ENSMUSG00000046434.15 | Hnrnpa1 | -0.268078 | 0.00235 | 0.0389965 |

| | | | | |
|---|---|---|---|---|
| ENSMUSG00000070923.4 | Klhl9 | -0.268888 | 0.00205 | 0.0356026 |
| ENSMUSG00000050912.15 | Tmem123 | -0.269675 | 0.0025 | 0.0405307 |
| ENSMUSG00000022533.13 | Atp13a3 | -0.270466 | 0.00245 | 0.0401306 |
| ENSMUSG00000022912.8 | Pros1 | -0.270499 | 0.0023 | 0.0386217 |
| ENSMUSG00000024614.6 | Tmx3 | -0.270993 | 0.00185 | 0.0333632 |
| ENSMUSG00000026781.15 | Acbd5 | -0.272405 | 0.00235 | 0.0389965 |
| ENSMUSG00000044408.6 | Sptssa | -0.272574 | 0.00215 | 0.0368347 |
| ENSMUSG00000027822.16 | Slc33a1 | -0.272833 | 0.00265 | 0.0418911 |
| ENSMUSG00000020592.14 | Sdc1 | -0.273594 | 0.0029 | 0.0445111 |
| ENSMUSG00000046841.4 | Ckap4 | -0.273775 | 0.00165 | 0.0308991 |
| ENSMUSG00000031165.6 | Was | -0.274809 | 0.00205 | 0.0356026 |
| ENSMUSG00000045679.13 | Pqlc3 | -0.275022 | 0.00175 | 0.0321078 |
| ENSMUSG00000021108.17 | Prkch | -0.275429 | 0.00265 | 0.0418911 |
| ENSMUSG00000031337.16 | Mtm1 | -0.277104 | 0.0032 | 0.0475054 |
| ENSMUSG00000051695.6 | Pcbp1 | -0.278097 | 0.0026 | 0.0414106 |
| ENSMUSG00000018822.7 | Sfrp5 | -0.278842 | 0.00205 | 0.0356026 |
| ENSMUSG00000020075.7 | Ddx21 | -0.279205 | 0.0015 | 0.0293926 |
| ENSMUSG00000029634.15 | Rnf6 | -0.279334 | 0.00325 | 0.047911 |
| ENSMUSG00000020720.13 | Psmd12 | -0.279853 | 0.0014 | 0.0281287 |
| ENSMUSG00000022797.15 | Tfrc | -0.279968 | 0.00295 | 0.0450604 |
| ENSMUSG00000061119.7 | Prcp | -0.280127 | 0.0019 | 0.0341193 |
| ENSMUSG00000022867.9 | Usp25 | -0.280964 | 0.0017 | 0.0314636 |
| ENSMUSG00000020120.15 | Plek | -0.281061 | 0.0028 | 0.0435563 |
| ENSMUSG00000037851.13 | Iars | -0.282093 | 0.0032 | 0.0475054 |
| ENSMUSG00000046079.16 | Lrrc8d | -0.282343 | 0.00135 | 0.0276053 |
| ENSMUSG00000028419.5 | Chmp5 | -0.283542 | 0.002 | 0.035364 |
| ENSMUSG00000030275.6 | Etnk1 | -0.284648 | 0.00235 | 0.0389965 |
| ENSMUSG00000024991.7 | Eif3a | -0.284764 | 0.0022 | 0.0374384 |
| ENSMUSG00000021686.4 | Ap3b1 | -0.284845 | 0.00155 | 0.0298193 |
| ENSMUSG00000015750.15 | Aph1a | -0.284951 | 0.0025 | 0.0405307 |
| ENSMUSG00000019731.13 | Slc35e1 | -0.285369 | 0.00165 | 0.0308991 |
| ENSMUSG00000027782.10 | Kpna4 | -0.28587 | 0.0025 | 0.0405307 |
| ENSMUSG00000009630.10 | Ppp2cb | -0.286176 | 0.0016 | 0.0303665 |
| ENSMUSG00000064289.15 | Tank | -0.286412 | 0.00195 | 0.0348199 |
| ENSMUSG00000029038.9 | Ssu72 | -0.286479 | 0.0033 | 0.0485353 |
| ENSMUSG00000022305.12 | Lrp12 | -0.28687 | 0.0016 | 0.0303665 |
| ENSMUSG00000031167.16 | Rbm3 | -0.286997 | 0.0012 | 0.0256986 |
| ENSMUSG00000061232.15 | H2-K1 | -0.287593 | 0.00225 | 0.038034 |
| ENSMUSG00000038774.8 | Ascc3 | -0.28775 | 0.00275 | 0.0429366 |
| ENSMUSG00000024098.5 | Twsg1 | -0.28834 | 0.00245 | 0.0401306 |
| ENSMUSG00000027287.14 | Snap23 | -0.288727 | 0.00345 | 0.0498167 |
| ENSMUSG00000063410.7 | Stk24 | -0.288991 | 0.0013 | 0.0271076 |
| ENSMUSG00000027342.14 | Pcna | -0.289118 | 0.0012 | 0.0256986 |
| ENSMUSG00000073639.5 | Rab18 | -0.289192 | 0.00135 | 0.0276053 |
| ENSMUSG00000047379.6 | B4gat1 | -0.289386 | 0.00325 | 0.047911 |
| ENSMUSG00000011179.7 | Odc1 | -0.289525 | 0.0021 | 0.0362229 |
| ENSMUSG00000036580.15 | Spg20 | -0.289591 | 0.0016 | 0.0303665 |
| ENSMUSG00000020495.13 | Smg8 | -0.289639 | 0.0032 | 0.0475054 |
| ENSMUSG00000037458.14 | Azin1 | -0.290545 | 0.00205 | 0.0356026 |
| ENSMUSG00000032763.10 | Ilvbl | -0.290879 | 0.00255 | 0.0411309 |
| ENSMUSG00000030220.13 | Arhgdib | -0.290993 | 0.00145 | 0.0286788 |
| ENSMUSG00000021814.16 | Anxa7 | -0.291828 | 0.0012 | 0.0256986 |
| ENSMUSG00000032112.9 | Trappc4 | -0.2924 | 0.0029 | 0.0445111 |
| ENSMUSG00000040550.16 | Otud6b | -0.29247 | 0.00135 | 0.0276053 |
| ENSMUSG00000051579.10 | Tceal8 | -0.292645 | 0.0018 | 0.0328824 |
| ENSMUSG00000024218.12 | Taf11 | -0.293154 | 0.0031 | 0.0466214 |
| ENSMUSG00000006304.14 | Arpc2 | -0.294053 | 0.00185 | 0.0333632 |
| ENSMUSG00000001833.16 | Septin7 | -0.294218 | 0.00085 | 0.0203711 |
| ENSMUSG00000024487.4 | Yipf5 | -0.29491 | 0.0012 | 0.0256986 |
| ENSMUSG00000054693.14 | Adam10 | -0.295678 | 0.0007 | 0.0179285 |
| ENSMUSG00000031095.15 | Cul4b | -0.295885 | 0.00325 | 0.047911 |
| ENSMUSG00000025037.6 | Maoa | -0.295927 | 0.0014 | 0.0281287 |
| ENSMUSG00000022314.9 | Rad21 | -0.296677 | 0.0007 | 0.0179285 |
| ENSMUSG00000023186.14 | Vwa5a | -0.297178 | 0.00115 | 0.0251808 |
| ENSMUSG00000029840.8 | Mtpn | -0.298449 | 0.00085 | 0.0203711 |
| ENSMUSG00000027698.14 | Nceh1 | -0.298889 | 0.00175 | 0.0321078 |

| | | | | |
|---|---|---|---|---|
| ENSMUSG00000022339.8 | Ebag9 | -0.299269 | 0.0027 | 0.04226 |
| ENSMUSG00000032349.13 | Elovl5 | -0.29941 | 0.0013 | 0.0271076 |
| ENSMUSG00000023025.15 | Larp4 | -0.299429 | 0.0009 | 0.0212086 |
| ENSMUSG00000015839.6 | Nfe2l2 | -0.301353 | 0.001 | 0.0226798 |
| ENSMUSG00000026223.15 | Itm2c | -0.301775 | 0.001 | 0.0226798 |
| ENSMUSG00000034158.9 | Lrrc58 | -0.302005 | 0.0008 | 0.0195422 |
| ENSMUSG00000025647.16 | Shisa5 | -0.30248 | 0.001 | 0.0226798 |
| ENSMUSG00000032412.8 | Atp1b3 | -0.30288 | 0.0009 | 0.0212086 |
| ENSMUSG00000049339.16 | Fam134a | -0.302988 | 0.0021 | 0.0362229 |
| ENSMUSG00000031353.13 | Rbbp7 | -0.303088 | 0.0025 | 0.0405307 |
| ENSMUSG00000031197.11 | Vbp1 | -0.303889 | 0.00135 | 0.0276053 |
| ENSMUSG00000024759.13 | Atl3 | -0.304808 | 0.00075 | 0.0188287 |
| ENSMUSG00000068798.10 | Rap1a | -0.30521 | 0.00215 | 0.0368347 |
| ENSMUSG00000026377.12 | Nifk | -0.305524 | 0.0008 | 0.0195422 |
| ENSMUSG00000036155.13 | Mgat5 | -0.305666 | 0.0006 | 0.0160143 |
| ENSMUSG00000038646.13 | Fam103a1 | -0.306391 | 0.00105 | 0.0236027 |
| ENSMUSG00000043832.13 | Clec4a3 | -0.306711 | 0.0011 | 0.0243808 |
| ENSMUSG00000021592.15 | Arsk | -0.306753 | 0.0024 | 0.0394646 |
| ENSMUSG00000041891.15 | Lman1 | -0.307426 | 0.001 | 0.0226798 |
| ENSMUSG00000059586.9 | Nsmce2 | -0.307512 | 0.00055 | 0.0152247 |
| ENSMUSG00000022200.7 | Golph3 | -0.30844 | 0.00035 | 0.011149 |
| ENSMUSG00000054079.12 | Utp18 | -0.308489 | 0.001 | 0.0226798 |
| ENSMUSG00000021094.9 | Dhrs7 | -0.308986 | 0.0007 | 0.0179285 |
| ENSMUSG00000095115.2 | Itpripl2 | -0.309192 | 0.0007 | 0.0179285 |
| ENSMUSG00000032199.12 | Polr2m | -0.309574 | 0.0008 | 0.0195422 |
| ENSMUSG00000027068.6 | Dhrs9 | -0.309626 | 0.0005 | 0.0142449 |
| ENSMUSG00000049516.9 | Spty2d1 | -0.310043 | 0.0005 | 0.0142449 |
| ENSMUSG00000004843.6 | Chmp2b | -0.31064 | 0.00035 | 0.011149 |
| ENSMUSG00000029998.14 | Pcyox1 | -0.311046 | 0.0004 | 0.0121904 |
| ENSMUSG00000032050.16 | Rdx | -0.311564 | 0.0006 | 0.0160143 |
| ENSMUSG00000093904.1 | Tomm20 | -0.3117 | 0.0005 | 0.0142449 |
| ENSMUSG00000018217.12 | Pmp22 | -0.312013 | 0.00035 | 0.011149 |
| ENSMUSG00000052656.12 | Rnf103 | -0.312283 | 0.00155 | 0.0298193 |
| ENSMUSG00000061666.6 | Gdpd1 | -0.312687 | 0.00145 | 0.0286788 |
| ENSMUSG00000027108.15 | Ola1 | -0.313261 | 0.0012 | 0.0256986 |
| ENSMUSG00000020841.5 | Cpd | -0.313264 | 0.0004 | 0.0121904 |
| ENSMUSG00000032440.12 | Tgfbr2 | -0.313467 | 0.0001 | 0.00449574 |
| ENSMUSG00000030105.8 | Arl8b | -0.313927 | 0.00045 | 0.0132986 |
| ENSMUSG00000029076.14 | Sdf4 | -0.313977 | 0.0014 | 0.0281287 |
| ENSMUSG00000023075.9 | Akirin1 | -0.314077 | 0.00045 | 0.0132986 |
| ENSMUSG00000032359.14 | Ctsh | -0.314918 | 0.0017 | 0.0314636 |
| ENSMUSG00000042042.14 | Csgalnact2 | -0.315234 | 0.0006 | 0.0160143 |
| ENSMUSG00000043252.8 | Tmem64 | -0.315494 | 0.00235 | 0.0389965 |
| ENSMUSG00000058254.12 | Tspan7 | -0.315507 | 0.0012 | 0.0256986 |
| ENSMUSG00000021072.12 | Tmx1 | -0.316059 | 0.00205 | 0.0356026 |
| ENSMUSG00000024072.8 | Yipf4 | -0.316209 | 0.0008 | 0.0195422 |
| ENSMUSG00000059089.4 | Fcgr4 | -0.316908 | 0.0005 | 0.0142449 |
| ENSMUSG00000045594.9 | Glb1 | -0.316919 | 0.00335 | 0.0488738 |
| ENSMUSG00000039270.9 | Megf9 | -0.316998 | 0.0027 | 0.04226 |
| ENSMUSG00000008763.16 | Man1a2 | -0.317361 | 0.0014 | 0.0281287 |
| ENSMUSG00000053128.14 | Rnf26 | -0.317654 | 0.00335 | 0.0488738 |
| ENSMUSG00000025915.14 | Sgk3 | -0.319092 | 0.00125 | 0.0265008 |
| ENSMUSG00000038622.7 | Med30 | -0.319487 | 0.00235 | 0.0389965 |
| ENSMUSG00000030659.14 | Nucb2 | -0.320029 | 0.00025 | 0.00905571 |
| ENSMUSG00000022663.3 | Atg3 | -0.320925 | 0.0004 | 0.0121904 |
| ENSMUSG00000021831.8 | Ero1l | -0.320971 | 0.0006 | 0.0160143 |
| ENSMUSG00000074785.4 | Plxnc1 | -0.32215 | 0.00045 | 0.0132986 |
| ENSMUSG00000055782.8 | Abcd2 | -0.322779 | 0.00175 | 0.0321078 |
| ENSMUSG00000016541.9 | Atxn10 | -0.322841 | 0.00025 | 0.00905571 |
| ENSMUSG00000028173.10 | Wls | -0.323053 | 0.00085 | 0.0203711 |
| ENSMUSG00000022403.14 | St13 | -0.323236 | 0.00135 | 0.0276053 |
| ENSMUSG00000027809.14 | Etfdh | -0.323606 | 0.0015 | 0.0293926 |
| ENSMUSG00000040451.17 | Sgms1 | -0.323768 | 0.0005 | 0.0142449 |
| ENSMUSG00000022664.11 | Slc35a5 | -0.324119 | 0.00085 | 0.0203711 |
| ENSMUSG00000029338.13 | Antxr2 | -0.324286 | 0.0003 | 0.0100619 |
| ENSMUSG00000020130.8 | Tbc1d15 | -0.324594 | 0.0004 | 0.0121904 |

| | | | | |
|---|---|---|---|---|
| ENSMUSG00000037370.13 | Enpp1 | -0.324612 | 0.0026 | 0.0414106 |
| ENSMUSG00000047139.8 | Cd24a | -0.324866 | 0.00115 | 0.0251808 |
| ENSMUSG00000000420.15 | Galnt1 | -0.325063 | 0.0003 | 0.0100619 |
| ENSMUSG00000022234.14 | Cct5 | -0.325859 | 0.0001 | 0.00449574 |
| ENSMUSG00000021811.6 | Dnajc9 | -0.3259 | 0.00215 | 0.0368347 |
| ENSMUSG00000027534.16 | Snx16 | -0.326448 | 0.00115 | 0.0251808 |
| ENSMUSG00000016206.6 | H2-M3 | -0.327859 | 0.00165 | 0.0308991 |
| ENSMUSG00000031647.10 | Mfap3l | -0.328959 | 0.00145 | 0.0286788 |
| ENSMUSG00000049409.9 | Prokr1 | -0.329384 | 0.00125 | 0.0265008 |
| ENSMUSG00000029253.12 | Cenpc1 | -0.329464 | 0.0017 | 0.0314636 |
| ENSMUSG00000079037.9 | Prnp | -0.329665 | 0.00025 | 0.00905571 |
| ENSMUSG00000001942.7 | Siae | -0.329768 | 0.0011 | 0.0243808 |
| ENSMUSG00000029569.9 | Tmem168 | -0.330408 | 0.0002 | 0.00775413 |
| ENSMUSG00000037075.6 | Rnf139 | -0.330663 | 0.00215 | 0.0368347 |
| ENSMUSG00000004383.17 | Large | -0.330956 | 0.00035 | 0.011149 |
| ENSMUSG00000022329.14 | Stk3 | -0.331686 | 0.00025 | 0.00905571 |
| ENSMUSG00000053012.17 | Krcc1 | -0.332079 | 0.00015 | 0.00627624 |
| ENSMUSG00000062082.16 | Cd200r4 | -0.332308 | 0.0002 | 0.00775413 |
| ENSMUSG00000025203.5 | Scd2 | -0.332794 | 0.00015 | 0.00627624 |
| ENSMUSG00000051185.9 | Fam174a | -0.333036 | 0.0003 | 0.0100619 |
| ENSMUSG00000041084.8 | Ostc | -0.333835 | 0.0003 | 0.0100619 |
| ENSMUSG00000067336.6 | Bmpr2 | -0.334625 | 0.00035 | 0.011149 |
| ENSMUSG00000037949.7 | Ano10 | -0.334854 | 0.0011 | 0.0243808 |
| ENSMUSG00000003316.14 | Glg1 | -0.334874 | 0.00035 | 0.011149 |
| ENSMUSG00000040747.9 | Cd53 | -0.335027 | 0.00235 | 0.0389965 |
| ENSMUSG00000051675.13 | Trim32 | -0.33534 | 0.00165 | 0.0308991 |
| ENSMUSG00000021248.8 | Tmed10 | -0.336553 | 0.0003 | 0.0100619 |
| ENSMUSG00000041439.15 | Mfsd6 | -0.337164 | 0.00075 | 0.0188287 |
| ENSMUSG00000027195.10 | Hsd17b12 | -0.337639 | 0.0011 | 0.0243808 |
| ENSMUSG00000022742.5 | Cpox | -0.338531 | 0.00045 | 0.0132986 |
| ENSMUSG00000073139.9 | BC023829 | -0.338838 | 0.00115 | 0.0251808 |
| ENSMUSG00000033557.17 | Fam20b | -0.339396 | 0.0002 | 0.00775413 |
| ENSMUSG00000040520.7 | Manea | -0.339589 | 0.0002 | 0.00775413 |
| ENSMUSG00000020101.14 | Vsir | -0.340474 | 0.0007 | 0.0179285 |
| ENSMUSG00000021665.7 | Hexb | -0.340511 | 0.0005 | 0.0142449 |
| ENSMUSG00000038212.15 | Mfsd14b | -0.340723 | 0.00015 | 0.00627624 |
| ENSMUSG00000035847.15 | Ids | -0.341298 | 0.00005 | 0.00258735 |
| ENSMUSG00000026356.15 | Dars | -0.341874 | 0.00015 | 0.00627624 |
| ENSMUSG00000000340.10 | Dbt | -0.342451 | 0.0004 | 0.0121904 |
| ENSMUSG00000032328.12 | Tmem30a | -0.34249 | 0.0001 | 0.00449574 |
| ENSMUSG00000032459.9 | Mrps22 | -0.343013 | 0.0012 | 0.0256986 |
| ENSMUSG00000028557.10 | Rnf11 | -0.3432 | 0.0025 | 0.0405307 |
| ENSMUSG00000021929.8 | Kpna3 | -0.343937 | 0.0001 | 0.00449574 |
| ENSMUSG00000022378.13 | Fam49b | -0.34433 | 0.00035 | 0.011149 |
| ENSMUSG00000020432.12 | Tcn2 | -0.344374 | 0.0001 | 0.00449574 |
| ENSMUSG00000042369.8 | Rbm45 | -0.345689 | 0.00035 | 0.011149 |
| ENSMUSG00000062949.13 | Atp11c | -0.346138 | 0.00025 | 0.00905571 |
| ENSMUSG00000031532.6 | Saraf | -0.346333 | 0.0002 | 0.00775413 |
| ENSMUSG00000028007.13 | Snx7 | -0.346505 | 0.00145 | 0.0286788 |
| ENSMUSG00000031246.14 | Sh3bgrl | -0.346645 | 0.0001 | 0.00449574 |
| ENSMUSG00000030987.5 | Stim1 | -0.347428 | 0.0003 | 0.0100619 |
| ENSMUSG00000035150.15 | Eif2s3x | -0.348048 | 0.0032 | 0.0475054 |
| ENSMUSG00000001281.9 | Itgb7 | -0.348197 | 0.00005 | 0.00258735 |
| ENSMUSG00000050148.9 | Ubqln2 | -0.348617 | 0.00005 | 0.00258735 |
| ENSMUSG00000026727.10 | Rsu1 | -0.348654 | 0.0001 | 0.00449574 |
| ENSMUSG00000026116.11 | Tmem131 | -0.34964 | 0.0011 | 0.0243808 |
| ENSMUSG00000028517.8 | Plpp3 | -0.349729 | 0.00005 | 0.00258735 |
| ENSMUSG00000025823.9 | Pdia4 | -0.349755 | 0.0002 | 0.00775413 |
| ENSMUSG00000031333.7 | Abcb7 | -0.349804 | 0.00025 | 0.00905571 |
| ENSMUSG00000024785.6 | Rcl1 | -0.349836 | 0.00255 | 0.0411309 |
| ENSMUSG00000074280.5 | Gm6166 | -0.350058 | 0.0005 | 0.0142449 |
| ENSMUSG00000020386.5 | Sar1b | -0.350195 | 0.00015 | 0.00627624 |
| ENSMUSG00000063895.6 | Nupl1 | -0.350368 | 0.001 | 0.0226798 |
| ENSMUSG00000019818.15 | Cd164 | -0.350494 | 0.0001 | 0.00449574 |
| ENSMUSG00000033207.6 | Mamdc2 | -0.351174 | 0.0002 | 0.00775413 |
| ENSMUSG00000024507.6 | Hsd17b4 | -0.351816 | 0.0028 | 0.0435563 |

| | | | | |
|---|---|---|---|---|
| ENSMUSG00000029672.16 | Fam3c | -0.352147 | 0.0007 | 0.0179285 |
| ENSMUSG00000021871.17 | Pnp | -0.353294 | 0.00095 | 0.0220588 |
| ENSMUSG00000062585.11 | Cnr2 | -0.353304 | 0.0003 | 0.0100619 |
| ENSMUSG00000003721.14 | Insig2 | -0.35411 | 0.003 | 0.0455497 |
| ENSMUSG00000043895.5 | S1pr2 | -0.354333 | 0.00025 | 0.00905571 |
| ENSMUSG00000024601.8 | Isoc1 | -0.354934 | 0.00095 | 0.0220588 |
| ENSMUSG00000031007.2 | Atp6ap2 | -0.3555 | 0.00015 | 0.00627624 |
| ENSMUSG00000026980.15 | Ly75 | -0.355541 | 0.001 | 0.0226798 |
| ENSMUSG00000061273.3 | Mmgt1 | -0.355599 | 0.00015 | 0.00627624 |
| ENSMUSG00000039202.12 | Abhd2 | -0.355735 | 0.0005 | 0.0142449 |
| ENSMUSG00000032475.15 | Nck1 | -0.355867 | 0.0012 | 0.0256986 |
| ENSMUSG00000039497.7 | Dse | -0.356458 | 0.0001 | 0.00449574 |
| ENSMUSG00000038147.13 | Cd84 | -0.357495 | 0.0013 | 0.0271076 |
| ENSMUSG00000026775.9 | Yme1l1 | -0.357587 | 0.00005 | 0.00258735 |
| ENSMUSG00000013662.5 | Atad1 | -0.357685 | 0.00005 | 0.00258735 |
| ENSMUSG00000022514.14 | Il1rap | -0.358564 | 0.003 | 0.0455497 |
| ENSMUSG00000039395.8 | Mreg | -0.358747 | 0.00165 | 0.0308991 |
| ENSMUSG00000028098.13 | Rnf115 | -0.358839 | 0.0001 | 0.00449574 |
| ENSMUSG00000033793.12 | Atp6v1h | -0.358842 | 0.00005 | 0.00258735 |
| ENSMUSG00000026012.2 | Cd28 | -0.358918 | 0.0001 | 0.00449574 |
| ENSMUSG00000040158.12 | Tax1bp3 | -0.359557 | 0.0019 | 0.0341193 |
| ENSMUSG00000014177.10 | Tvp23b | -0.360031 | 0.00045 | 0.0132986 |
| ENSMUSG00000024896.8 | Minpp1 | -0.361317 | 0.00025 | 0.00905571 |
| ENSMUSG00000078427.2 | Sarnp | -0.361889 | 0.00025 | 0.00905571 |
| ENSMUSG00000019782.9 | Rwdd1 | -0.362458 | 0.00015 | 0.00627624 |
| ENSMUSG00000074825.4 | Itpripl1 | -0.36309 | 0.0025 | 0.0405307 |
| ENSMUSG00000038179.13 | Slamf7 | -0.363468 | 0.00045 | 0.0132986 |
| ENSMUSG00000027995.10 | Tlr2 | -0.363899 | 0.0026 | 0.0414106 |
| ENSMUSG00000008540.11 | Mgst1 | -0.36399 | 0.00015 | 0.00627624 |
| ENSMUSG00000022136.7 | Dnajc3 | -0.364115 | 0.00005 | 0.00258735 |
| ENSMUSG00000037434.7 | Slc30a1 | -0.364568 | 0.00005 | 0.00258735 |
| ENSMUSG00000029426.8 | Scarb2 | -0.364799 | 0.00005 | 0.00258735 |
| ENSMUSG00000060090.16 | Rp2 | -0.364928 | 0.00015 | 0.00627624 |
| ENSMUSG00000015932.8 | Dstn | -0.36703 | 0.00005 | 0.00258735 |
| ENSMUSG00000027366.12 | Sppl2a | -0.367535 | 0.002 | 0.035364 |
| ENSMUSG00000042712.10 | Wbp5 | -0.367738 | 0.00005 | 0.00258735 |
| ENSMUSG00000033863.1 | Klf9 | -0.368354 | 0.00055 | 0.0152247 |
| ENSMUSG00000029781.7 | Fkbp9 | -0.368467 | 0.00045 | 0.0132986 |
| ENSMUSG00000066441.14 | Rdh11 | -0.36924 | 0.00005 | 0.00258735 |
| ENSMUSG00000020048.13 | Hsp90b1 | -0.371044 | 0.0003 | 0.0100619 |
| ENSMUSG00000031608.13 | Galnt7 | -0.37263 | 0.0001 | 0.00449574 |
| ENSMUSG00000062070.12 | Pgk1 | -0.373352 | 0.00005 | 0.00258735 |
| ENSMUSG00000037071.2 | Scd1 | -0.37379 | 0.00045 | 0.0132986 |
| ENSMUSG00000030613.13 | Ccdc90b | -0.37406 | 0.0004 | 0.0121904 |
| ENSMUSG00000022257.3 | Laptm4b | -0.374818 | 0.0009 | 0.0212086 |
| ENSMUSG00000031604.10 | Msmo1 | -0.374852 | 0.00015 | 0.00627624 |
| ENSMUSG00000055639.16 | Dach1 | -0.375457 | 0.00125 | 0.0265008 |
| ENSMUSG00000019966.17 | Kitl | -0.375544 | 0.001 | 0.0226798 |
| ENSMUSG00000009207.15 | Lnp | -0.375553 | 0.0006 | 0.0160143 |
| ENSMUSG00000015854.7 | Cd5l | -0.375955 | 0.00005 | 0.00258735 |
| ENSMUSG00000062078.14 | Qk | -0.376398 | 0.00005 | 0.00258735 |
| ENSMUSG00000028383.17 | Hsdl2 | -0.376594 | 0.0001 | 0.00449574 |
| ENSMUSG00000059447.13 | Hadhb | -0.376745 | 0.00005 | 0.00258735 |
| ENSMUSG00000067591.12 | Klra3 | -0.376851 | 0.00315 | 0.0472053 |
| ENSMUSG00000029250.5 | Polr2b | -0.376927 | 0.0022 | 0.0374384 |
| ENSMUSG00000036764.11 | Dnajc12 | -0.377043 | 0.0016 | 0.0303665 |
| ENSMUSG00000021109.13 | Hif1a | -0.377719 | 0.0001 | 0.00449574 |
| ENSMUSG00000035164.7 | Zc3h12c | -0.38003 | 0.0032 | 0.0475054 |
| ENSMUSG00000029767.16 | Calu | -0.380567 | 0.00005 | 0.00258735 |
| ENSMUSG00000033953.10 | Ppp3r1 | -0.38224 | 0.00075 | 0.0188287 |
| ENSMUSG00000072235.5 | Tuba1a | -0.38428 | 0.00025 | 0.00905571 |
| ENSMUSG00000030786.18 | Itgam | -0.384339 | 0.00005 | 0.00258735 |
| ENSMUSG00000023921.8 | Mut | -0.385371 | 0.00005 | 0.00258735 |
| ENSMUSG00000031521.5 | Aga | -0.385534 | 0.00005 | 0.00258735 |
| ENSMUSG00000020077.14 | Srgn | -0.385788 | 0.0001 | 0.00449574 |
| ENSMUSG00000010517.7 | Faf1 | -0.38602 | 0.0016 | 0.0303665 |

| | | | | |
|---|---|---|---|---|
| ENSMUSG00000022110.13 | Sucla2 | -0.386277 | 0.0014 | 0.0281287 |
| ENSMUSG00000045237.5 | 1110012L19Rik | -0.38633 | 0.00045 | 0.0132986 |
| ENSMUSG00000030208.15 | Emp1 | -0.386647 | 0.00005 | 0.00258735 |
| ENSMUSG00000043019.12 | Edem3 | -0.388017 | 0.00005 | 0.00258735 |
| ENSMUSG00000035372.1 | 1810055G02Rik | -0.388668 | 0.0023 | 0.0386217 |
| ENSMUSG00000046324.12 | Ermp1 | -0.389308 | 0.0004 | 0.0121904 |
| ENSMUSG00000022892.10 | App | -0.389837 | 0.00005 | 0.00258735 |
| ENSMUSG00000066324.2 | Impad1 | -0.390033 | 0.00005 | 0.00258735 |
| ENSMUSG00000032965.10 | Ift57 | -0.390291 | 0.00335 | 0.0488738 |
| ENSMUSG00000017057.9 | Il13ra1 | -0.39076 | 0.00005 | 0.00258735 |
| ENSMUSG00000024074.7 | Crim1 | -0.391183 | 0.0013 | 0.0271076 |
| ENSMUSG00000020091.14 | Eif4ebp2 | -0.391313 | 0.00205 | 0.0356026 |
| ENSMUSG00000021213.11 | Akr1c13 | -0.391852 | 0.0003 | 0.0100619 |
| ENSMUSG00000063800.5 | Prpf38a | -0.393238 | 0.0005 | 0.0142449 |
| ENSMUSG00000011752.6 | Pgam1 | -0.394121 | 0.00005 | 0.00258735 |
| ENSMUSG00000030096.8 | Slc6a6 | -0.394469 | 0.0001 | 0.00449574 |
| ENSMUSG00000022876.17 | Samsn1 | -0.395051 | 0.00115 | 0.0251808 |
| ENSMUSG00000060147.15 | Serpinb6a | -0.395345 | 0.00045 | 0.0132986 |
| ENSMUSG00000020585.4 | Laptm4a | -0.395473 | 0.00005 | 0.00258735 |
| ENSMUSG00000029915.14 | Clec5a | -0.39587 | 0.00015 | 0.00627624 |
| ENSMUSG00000028111.4 | Ctsk | -0.397706 | 0.00015 | 0.00627624 |
| ENSMUSG00000071855.4 | Ccdc112 | -0.400304 | 0.0018 | 0.0328824 |
| ENSMUSG00000031696.9 | Vps35 | -0.401001 | 0.00005 | 0.00258735 |
| ENSMUSG00000078945.10 | Naip2 | -0.401369 | 0.00005 | 0.00258735 |
| ENSMUSG00000033964.12 | Zbtb41 | -0.40154 | 0.00035 | 0.011149 |
| ENSMUSG00000024085.13 | Man2a1 | -0.401778 | 0.00005 | 0.00258735 |
| ENSMUSG00000019948.3 | Actr6 | -0.403059 | 0.001 | 0.0226798 |
| ENSMUSG00000079293.11 | Clec7a | -0.403212 | 0.0001 | 0.00449574 |
| ENSMUSG00000021190.14 | Lgmn | -0.404262 | 0.0004 | 0.0121904 |
| ENSMUSG00000050786.8 | Ccdc126 | -0.406385 | 0.00185 | 0.0333632 |
| ENSMUSG00000016534.15 | Lamp2 | -0.406979 | 0.00015 | 0.00627624 |
| ENSMUSG00000020952.9 | Scfd1 | -0.40731 | 0.0001 | 0.00449574 |
| ENSMUSG00000000787.12 | Ddx3x | -0.408048 | 0.00005 | 0.00258735 |
| ENSMUSG00000049439.13 | Cyp20a1 | -0.408969 | 0.0002 | 0.00775413 |
| ENSMUSG00000011114.17 | Tbrg1 | -0.409124 | 0.00005 | 0.00258735 |
| ENSMUSG00000047881.14 | Rell1 | -0.409809 | 0.00005 | 0.00258735 |
| ENSMUSG00000035840.6 | Lysmd3 | -0.410315 | 0.00155 | 0.0298193 |
| ENSMUSG00000032667.17 | Pon2 | -0.410608 | 0.00005 | 0.00258735 |
| ENSMUSG00000018927.3 | Ccl6 | -0.411601 | 0.0001 | 0.00449574 |
| ENSMUSG00000022667.17 | Cd200r1 | -0.411947 | 0.00005 | 0.00258735 |
| ENSMUSG00000039005.13 | Tlr4 | -0.412563 | 0.00005 | 0.00258735 |
| ENSMUSG00000022757.18 | Tfg | -0.412758 | 0.00005 | 0.00258735 |
| ENSMUSG00000038280.12 | Ostm1 | -0.413917 | 0.00005 | 0.00258735 |
| ENSMUSG00000026094.14 | Stk17b | -0.415601 | 0.00005 | 0.00258735 |
| ENSMUSG00000022255.15 | Mtdh | -0.417988 | 0.0003 | 0.0100619 |
| ENSMUSG00000025544.13 | Tm9sf2 | -0.418672 | 0.00005 | 0.00258735 |
| ENSMUSG00000029366.10 | Dck | -0.420133 | 0.00085 | 0.0203711 |
| ENSMUSG00000029571.14 | Tmem106b | -0.420371 | 0.00005 | 0.00258735 |
| ENSMUSG00000046186.8 | Cd109 | -0.420411 | 0.00005 | 0.00258735 |
| ENSMUSG00000052914.4 | Cyp2j6 | -0.420732 | 0.00035 | 0.011149 |
| ENSMUSG00000046873.18 | Mbtps2 | -0.421687 | 0.00005 | 0.00258735 |
| ENSMUSG00000021116.9 | Eif2s1 | -0.421782 | 0.00005 | 0.00258735 |
| ENSMUSG00000027828.12 | Ssr3 | -0.422765 | 0.00005 | 0.00258735 |
| ENSMUSG00000025809.15 | Itgb1 | -0.424834 | 0.00005 | 0.00258735 |
| ENSMUSG00000022722.18 | Arl6 | -0.426771 | 0.0008 | 0.0195422 |
| ENSMUSG00000034723.11 | Tmx4 | -0.430825 | 0.00005 | 0.00258735 |
| ENSMUSG00000103847.1 | Gm20056 | -0.43083 | 0.0002 | 0.00775413 |
| ENSMUSG00000025804.4 | Ccr1 | -0.431147 | 0.00005 | 0.00258735 |
| ENSMUSG00000029836.15 | Cbx3 | -0.432562 | 0.00145 | 0.0286788 |
| ENSMUSG00000024378.9 | Stard4 | -0.432577 | 0.00075 | 0.0188287 |
| ENSMUSG00000021065.15 | Fut8 | -0.433182 | 0.00005 | 0.00258735 |
| ENSMUSG00000024425.3 | Ndfip1 | -0.435169 | 0.00005 | 0.00258735 |
| ENSMUSG00000045092.8 | S1pr1 | -0.436563 | 0.00005 | 0.00258735 |
| ENSMUSG00000038844.8 | Kif16b | -0.437226 | 0.0009 | 0.0212086 |
| ENSMUSG00000000001.4 | Gnai3 | -0.437419 | 0.00005 | 0.00258735 |
| ENSMUSG00000029265.4 | Dr1 | -0.438904 | 0.00005 | 0.00258735 |

| | | | | |
|---|---|---|---|---|
| ENSMUSG00000021866.15 | Anxa11 | -0.438956 | 0.00005 | 0.00258735 |
| ENSMUSG00000017707.9 | Serinc3 | -0.439101 | 0.00005 | 0.00258735 |
| ENSMUSG00000056429.4 | Tgoln1 | -0.439593 | 0.00005 | 0.00258735 |
| ENSMUSG00000043998.10 | Mgat2 | -0.441046 | 0.00005 | 0.00258735 |
| ENSMUSG00000020571.12 | Pdia6 | -0.441169 | 0.00005 | 0.00258735 |
| ENSMUSG00000035561.5 | Aldh1b1 | -0.441849 | 0.00005 | 0.00258735 |
| ENSMUSG00000025531.14 | Chm | -0.442254 | 0.00005 | 0.00258735 |
| ENSMUSG00000022003.6 | Slc25a30 | -0.44473 | 0.00235 | 0.0389965 |
| ENSMUSG00000026342.10 | Slc35f5 | -0.448135 | 0.00005 | 0.00258735 |
| ENSMUSG00000059588.13 | Calcrl | -0.449945 | 0.0002 | 0.00775413 |
| ENSMUSG00000052738.14 | Suclg1 | -0.450026 | 0.00005 | 0.00258735 |
| ENSMUSG00000026986.14 | Hnmt | -0.45104 | 0.00005 | 0.00258735 |
| ENSMUSG00000019796.12 | Lrp11 | -0.453677 | 0.0001 | 0.00449574 |
| ENSMUSG00000101188.1 | Eif4a-ps4 | -0.453783 | 0.00005 | 0.00258735 |
| ENSMUSG00000026365.15 | Cfh | -0.456038 | 0.00005 | 0.00258735 |
| ENSMUSG00000032252.14 | Glce | -0.457293 | 0.0002 | 0.00775413 |
| ENSMUSG00000026864.13 | Hspa5 | -0.45858 | 0.00005 | 0.00258735 |
| ENSMUSG00000024121.13 | Atp6v0c | -0.459986 | 0.00005 | 0.00258735 |
| ENSMUSG00000002718.14 | Cse1l | -0.460136 | 0.0034 | 0.0493195 |
| ENSMUSG00000028270.12 | Gbp2 | -0.460988 | 0.00005 | 0.00258735 |
| ENSMUSG00000053289.7 | Ddx10 | -0.461082 | 0.00005 | 0.00258735 |
| ENSMUSG00000032932.14 | Hspa13 | -0.461095 | 0.00005 | 0.00258735 |
| ENSMUSG00000038633.5 | Degs1 | -0.46173 | 0.00005 | 0.00258735 |
| ENSMUSG00000082433.2 | Gm9025 | -0.462721 | 0.0007 | 0.0179285 |
| ENSMUSG00000069515.5 | Lyz1 | -0.462821 | 0.00005 | 0.00258735 |
| ENSMUSG00000028274.17 | Rngtt | -0.463529 | 0.00005 | 0.00258735 |
| ENSMUSG00000031703.8 | Itfg1 | -0.464557 | 0.00005 | 0.00258735 |
| ENSMUSG00000019917.9 | Sept10 | -0.465852 | 0.0003 | 0.0100619 |
| ENSMUSG00000021728.7 | Emb | -0.467693 | 0.00005 | 0.00258735 |
| ENSMUSG00000030560.17 | Ctsc | -0.467694 | 0.00005 | 0.00258735 |
| ENSMUSG00000042271.13 | Nxt2 | -0.469372 | 0.00005 | 0.00258735 |
| ENSMUSG00000042684.8 | Npl | -0.469555 | 0.0003 | 0.0100619 |
| ENSMUSG00000052821.3 | Cysltr1 | -0.470871 | 0.0024 | 0.0394646 |
| ENSMUSG00000022351.14 | Sqle | -0.471421 | 0.00005 | 0.00258735 |
| ENSMUSG00000053253.16 | Ndfip2 | -0.471635 | 0.00005 | 0.00258735 |
| ENSMUSG00000020738.16 | Sumo2 | -0.471832 | 0.00005 | 0.00258735 |
| ENSMUSG00000073987.4 | Ggh | -0.472007 | 0.00005 | 0.00258735 |
| ENSMUSG00000039197.9 | Adk | -0.472059 | 0.00005 | 0.00258735 |
| ENSMUSG00000025986.6 | Slc39a10 | -0.472174 | 0.00005 | 0.00258735 |
| ENSMUSG00000062270.13 | Morf4l1 | -0.480108 | 0.00005 | 0.00258735 |
| ENSMUSG00000020368.15 | Canx | -0.481378 | 0.00005 | 0.00258735 |
| ENSMUSG00000084159.1 | Gm12696 | -0.48204 | 0.0006 | 0.0160143 |
| ENSMUSG00000016319.3 | Slc25a5 | -0.485087 | 0.00005 | 0.00258735 |
| ENSMUSG00000054404.12 | Slfn5 | -0.486071 | 0.00005 | 0.00258735 |
| ENSMUSG00000054843.8 | Atrnl1 | -0.486594 | 0.0004 | 0.0121904 |
| ENSMUSG00000031591.14 | Asah1 | -0.486927 | 0.00005 | 0.00258735 |
| ENSMUSG00000019849.10 | Prep | -0.487158 | 0.00005 | 0.00258735 |
| ENSMUSG00000025016.10 | Tm9sf3 | -0.489441 | 0.00005 | 0.00258735 |
| ENSMUSG00000057363.12 | Uxs1 | -0.490569 | 0.00005 | 0.00258735 |
| ENSMUSG00000025903.14 | Lypla1 | -0.492047 | 0.00005 | 0.00258735 |
| ENSMUSG00000025934.15 | Gsta3 | -0.493625 | 0.00035 | 0.011149 |
| ENSMUSG00000031555.15 | Adam9 | -0.4945 | 0.00005 | 0.00258735 |
| ENSMUSG00000022021.13 | Diaph3 | -0.497862 | 0.0017 | 0.0314636 |
| ENSMUSG00000020458.16 | Rtn4 | -0.498963 | 0.00005 | 0.00258735 |
| ENSMUSG00000037072.15 | Sept15 | -0.499071 | 0.00305 | 0.0460881 |
| ENSMUSG00000051255.5 | Gm6563 | -0.501068 | 0.00035 | 0.011149 |
| ENSMUSG00000024781.15 | Lipa | -0.501864 | 0.00005 | 0.00258735 |
| ENSMUSG00000025289.15 | Prdx4 | -0.502013 | 0.00005 | 0.00258735 |
| ENSMUSG00000032320.7 | Rcn2 | -0.504445 | 0.00005 | 0.00258735 |
| ENSMUSG00000021583.6 | Erap1 | -0.504523 | 0.00005 | 0.00258735 |
| ENSMUSG00000038642.10 | Ctss | -0.506924 | 0.0014 | 0.0281287 |
| ENSMUSG00000025017.9 | Pik3ap1 | -0.508756 | 0.00005 | 0.00258735 |
| ENSMUSG00000038503.15 | Mesdc2 | -0.512344 | 0.00135 | 0.0276053 |
| ENSMUSG00000039007.9 | Cpq | -0.514232 | 0.00005 | 0.00258735 |
| ENSMUSG00000042082.6 | Arsb | -0.514893 | 0.00005 | 0.00258735 |
| ENSMUSG00000075470.1 | Alg10b | -0.51616 | 0.00005 | 0.00258735 |

| | | | | |
|---|---|---|---|---|
| ENSMUSG00000039480.13 | Nt5dc1 | -0.517705 | 0.00005 | 0.00258735 |
| ENSMUSG00000020650.14 | Bcap29 | -0.519899 | 0.00005 | 0.00258735 |
| ENSMUSG00000105987.4 | AI506816 | -0.520442 | 0.00005 | 0.00258735 |
| ENSMUSG00000048970.9 | C1galt1c1 | -0.525606 | 0.00005 | 0.00258735 |
| ENSMUSG00000026617.16 | Bpnt1 | -0.526127 | 0.00005 | 0.00258735 |
| ENSMUSG00000047434.13 | Xxylt1 | -0.527624 | 0.00005 | 0.00258735 |
| ENSMUSG00000056220.14 | Pla2g4a | -0.528848 | 0.00005 | 0.00258735 |
| ENSMUSG00000031447.7 | Lamp1 | -0.529194 | 0.00005 | 0.00258735 |
| ENSMUSG00000022122.14 | Ednrb | -0.529383 | 0.00005 | 0.00258735 |
| ENSMUSG00000019877.10 | Serinc1 | -0.53006 | 0.00005 | 0.00258735 |
| ENSMUSG00000094724.7 | Rnaset2b | -0.531151 | 0.00015 | 0.00627624 |
| ENSMUSG00000003746.15 | Man1a | -0.531392 | 0.00005 | 0.00258735 |
| ENSMUSG00000054640.14 | Slc8a1 | -0.532602 | 0.0031 | 0.0466214 |
| ENSMUSG00000100801.1 | Gm15459 | -0.534091 | 0.0001 | 0.00449574 |
| ENSMUSG00000036503.13 | Rnf13 | -0.534171 | 0.00005 | 0.00258735 |
| ENSMUSG00000047126.17 | Cltc | -0.536389 | 0.003 | 0.0455497 |
| ENSMUSG00000028563.16 | Tm2d1 | -0.537236 | 0.00005 | 0.00258735 |
| ENSMUSG00000102869.1 | 2900097C17Rik | -0.538284 | 0.00005 | 0.00258735 |
| ENSMUSG00000023078.6 | Cxcl13 | -0.543386 | 0.0007 | 0.0179285 |
| ENSMUSG00000035133.8 | Arhgap5 | -0.547419 | 0.00005 | 0.00258735 |
| ENSMUSG00000050931.7 | Sgms2 | -0.552774 | 0.00015 | 0.00627624 |
| ENSMUSG00000026361.8 | Cdc73 | -0.553633 | 0.00005 | 0.00258735 |
| ENSMUSG00000082536.1 | Gm13456 | -0.554294 | 0.0005 | 0.0142449 |
| ENSMUSG00000004730.14 | Adgre1 | -0.555572 | 0.00005 | 0.00258735 |
| ENSMUSG00000104913.1 | Gm6560 | -0.557574 | 0.00305 | 0.0460881 |
| ENSMUSG00000024228.12 | Nudt12 | -0.558051 | 0.0006 | 0.0160143 |
| ENSMUSG00000032314.14 | Etfa | -0.559393 | 0.00005 | 0.00258735 |
| ENSMUSG00000045799.5 | Gm9800 | -0.568633 | 0.00005 | 0.00258735 |
| ENSMUSG00000019872.13 | Smpdl3a | -0.569659 | 0.00005 | 0.00258735 |
| ENSMUSG00000022853.6 | Ehhadh | -0.572486 | 0.00025 | 0.00905571 |
| ENSMUSG00000027487.11 | Cdk5rap1 | -0.574024 | 0.0002 | 0.00775413 |
| ENSMUSG00000035202.7 | Lars2 | -0.575402 | 0.00005 | 0.00258735 |
| ENSMUSG00000044103.4 | Il1f9 | -0.57913 | 0.00155 | 0.0298193 |
| ENSMUSG00000037852.8 | Cpe | -0.579595 | 0.00005 | 0.00258735 |
| ENSMUSG00000052428.10 | Tmco1 | -0.585152 | 0.00005 | 0.00258735 |
| ENSMUSG00000020570.14 | Sypl | -0.592039 | 0.00005 | 0.00258735 |
| ENSMUSG00000023845.6 | Lnpep | -0.593034 | 0.00005 | 0.00258735 |
| ENSMUSG00000017831.7 | Rab5a | -0.597619 | 0.00005 | 0.00258735 |
| ENSMUSG00000033184.14 | Tmed7 | -0.606223 | 0.00005 | 0.00258735 |
| ENSMUSG00000022092.10 | Ppp3cc | -0.607301 | 0.00005 | 0.00258735 |
| ENSMUSG00000098178.1 | Gm42418 | -0.61038 | 0.0002 | 0.00775413 |
| ENSMUSG00000056234.14 | Ncoa4 | -0.626312 | 0.0005 | 0.0142449 |
| ENSMUSG00000002944.15 | Cd36 | -0.629375 | 0.00005 | 0.00258735 |
| ENSMUSG00000074419.11 | Gm15448 | -0.629836 | 0.0001 | 0.00449574 |
| ENSMUSG00000027963.14 | Extl2 | -0.631997 | 0.00005 | 0.00258735 |
| ENSMUSG00000024887.9 | Asah2 | -0.638378 | 0.00005 | 0.00258735 |
| ENSMUSG00000028156.12 | Eif4e | -0.686189 | 0.00205 | 0.0356026 |
| ENSMUSG00000024298.14 | Zfp871 | -0.686204 | 0.0001 | 0.00449574 |
| ENSMUSG00000060703.12 | Cd302 | -0.701273 | 0.00005 | 0.00258735 |
| ENSMUSG00000048234.13 | Rnf149 | -0.708054 | 0.00005 | 0.00258735 |
| ENSMUSG00000059195.10 | Gm12715 | -0.724215 | 0.00005 | 0.00258735 |
| ENSMUSG00000024600.8 | Slc27a6 | -0.734748 | 0.00005 | 0.00258735 |
| ENSMUSG00000048732.5 | Klhl11 | -0.744624 | 0.0008 | 0.0195422 |
| ENSMUSG00000014905.3 | Dnajb9 | -0.744725 | 0.00005 | 0.00258735 |
| ENSMUSG00000039530.17 | Tusc3 | -0.755475 | 0.00005 | 0.00258735 |
| ENSMUSG00000027834.15 | Serpini1 | -0.779603 | 0.0017 | 0.0314636 |
| ENSMUSG00000024084.7 | Qpct | -0.792036 | 0.00005 | 0.00258735 |
| ENSMUSG00000033792.12 | Atp7a | -0.811561 | 0.0009 | 0.0212086 |
| ENSMUSG00000067714.13 | Lpar5 | -0.84304 | 0.00055 | 0.0152247 |
| ENSMUSG00000089942.9 | Pira2 | -0.849296 | 0.00005 | 0.00258735 |
| ENSMUSG00000035142.18 | Nubpl | -0.85251 | 0.00185 | 0.0333632 |
| ENSMUSG00000079625.2 | Tm4sf19 | -0.862598 | 0.00005 | 0.00258735 |
| ENSMUSG00000034488.14 | Edil3 | -0.878376 | 0.0001 | 0.00449574 |
| ENSMUSG00000079685.9 | Ulbp1 | -0.895772 | 0.00005 | 0.00258735 |
| ENSMUSG00000081723.1 | Gm15931 | -0.897487 | 0.00005 | 0.00258735 |
| ENSMUSG00000081121.1 | Gm12791 | -0.938691 | 0.00235 | 0.0389965 |

| | | | | |
|---|---|---|---|---|
| ENSMUSG00000090675.1 | Olfr111 | -0.943043 | 0.00025 | 0.00905571 |
| ENSMUSG00000031138.4 | F9 | -0.951283 | 0.00225 | 0.038034 |
| ENSMUSG00000053205.8 | Styx | -0.959008 | 0.0004 | 0.0121904 |
| ENSMUSG00000028262.13 | Clca3a2 | -0.969047 | 0.0006 | 0.0160143 |
| ENSMUSG00000067161.6 | Gm5560 | -1.01026 | 0.0026 | 0.0414106 |
| ENSMUSG00000074417.9 | Gm14548 | -1.02821 | 0.00005 | 0.00258735 |
| ENSMUSG00000038594.8 | Cep85l | -1.04266 | 0.00145 | 0.0286788 |
| ENSMUSG00000090124.7 | Ugt1a7c | -1.06383 | 0.0002 | 0.00775413 |
| ENSMUSG00000025257.5 | Ribc1 | -1.08476 | 0.0003 | 0.0100619 |
| ENSMUSG00000065037.1 | Rn7sk | -1.08669 | 0.00005 | 0.00258735 |
| ENSMUSG00000025019.15 | Lcor | -1.15671 | 0.00005 | 0.00258735 |
| ENSMUSG00000054178.1 | Gm9938 | -1.18102 | 0.00135 | 0.0276053 |
| ENSMUSG00000109836.1 | RP24-464B16.1 | -1.23736 | 0.00005 | 0.00258735 |
| ENSMUSG00000020728.17 | Cep112 | -1.28726 | 0.001 | 0.0226798 |
| ENSMUSG00000108659.1 | Gm34121 | -1.33781 | 0.0023 | 0.0386217 |
| ENSMUSG00000079884.6 | Gm10698 | -1.51509 | 0.00335 | 0.0488738 |
| ENSMUSG00000030156.5 | Cd69 | -1.65839 | 0.00085 | 0.0203711 |

**Table S6. Genes significantly regulated at and adjusted p-value (q-value)<0.05 in *Ldah* -KO PM treated with oxLDL.**

Data were analyzed using the Wald test (two-tailed) followed by the Benjamini–Hochberg test to estimate the false discovery rate adjusted p-value (q_value).

| gene_id | gene synonim | log2_fold change_KO/WT | p_value | q_value |
|---|---|---|---|---|
| ENSMUSG00000000753.15 | Serpinf1 | -1.49762 | 0.00005 | 0.0114713 |
| ENSMUSG00000001119.7 | Col6a1 | -1.14729 | 0.00005 | 0.0114713 |
| ENSMUSG00000001506.10 | Col1a1 | -1.37587 | 0.00005 | 0.0114713 |
| ENSMUSG00000002020.15 | Ltbp2 | -1.39651 | 0.0001 | 0.0196651 |
| ENSMUSG00000002900.15 | Lamb1 | -1.60141 | 0.0002 | 0.0321792 |
| ENSMUSG00000005397.7 | Nid1 | -1.1491 | 0.00005 | 0.0114713 |
| ENSMUSG00000012428.9 | Steap4 | -1.12344 | 0.00005 | 0.0114713 |
| ENSMUSG00000014329.14 | Bicc1 | -1.29402 | 0.00005 | 0.0114713 |
| ENSMUSG00000014599.10 | Csf1 | -1.09559 | 0.00035 | 0.0498408 |
| ENSMUSG00000015854.7 | Cd5l | 0.645319 | 0.00005 | 0.0114713 |
| ENSMUSG00000016252.7 | Atp5e | 0.571493 | 0.00015 | 0.0265479 |
| ENSMUSG00000016458.13 | Wt1 | -0.935166 | 0.00015 | 0.0265479 |
| ENSMUSG00000018593.12 | Sparc | -0.997256 | 0.00005 | 0.0114713 |
| ENSMUSG00000018822.7 | Sfrp5 | -0.723961 | 0.00005 | 0.0114713 |
| ENSMUSG00000019852.6 | Arfgef3 | -0.79976 | 0.0002 | 0.0321792 |
| ENSMUSG00000019929.15 | Dcn | -0.67407 | 0.00005 | 0.0114713 |
| ENSMUSG00000019997.11 | Ctgf | -1.14922 | 0.00005 | 0.0114713 |
| ENSMUSG00000020241.13 | Col6a2 | -0.938179 | 0.00005 | 0.0114713 |
| ENSMUSG00000020427.11 | Igfbp3 | -1.76014 | 0.00005 | 0.0114713 |
| ENSMUSG00000020473.13 | Aebp1 | -1.23365 | 0.00005 | 0.0114713 |
| ENSMUSG00000021390.5 | Ogn | -1.5494 | 0.00005 | 0.0114713 |
| ENSMUSG00000021745.13 | Ptprg | -3.20287 | 0.00005 | 0.0114713 |
| ENSMUSG00000022098.9 | Bmp1 | -0.931806 | 0.00025 | 0.0397083 |
| ENSMUSG00000022425.15 | Enpp2 | -1.28249 | 0.00005 | 0.0114713 |
| ENSMUSG00000022665.14 | Ccdc80 | -1.00855 | 0.00005 | 0.0114713 |
| ENSMUSG00000023078.6 | Cxcl13 | -1.64697 | 0.00005 | 0.0114713 |
| ENSMUSG00000024087.3 | Cyp1b1 | -1.2394 | 0.00005 | 0.0114713 |
| ENSMUSG00000024164.15 | C3 | -1.17284 | 0.00005 | 0.0114713 |
| ENSMUSG00000024304.14 | Cdh2 | -1.09516 | 0.00005 | 0.0114713 |
| ENSMUSG00000024529.13 | Lox | -1.0114 | 0.0001 | 0.0196651 |
| ENSMUSG00000026042.16 | Col5a2 | -1.12524 | 0.00005 | 0.0114713 |
| ENSMUSG00000026072.12 | Il1r1 | -1.18295 | 0.00005 | 0.0114713 |
| ENSMUSG00000026193.15 | Fn1 | -1.13939 | 0.00005 | 0.0114713 |
| ENSMUSG00000026208.9 | Des | -1.24421 | 0.00005 | 0.0114713 |
| ENSMUSG00000026399.12 | Cd55 | -0.810928 | 0.00005 | 0.0114713 |
| ENSMUSG00000027210.20 | Meis2 | -2.05303 | 0.00005 | 0.0114713 |
| ENSMUSG00000027360.5 | Hdc | -3.18431 | 0.00035 | 0.0498408 |
| ENSMUSG00000027797.15 | Dclk1 | -1.89016 | 0.0001 | 0.0196651 |
| ENSMUSG00000028195.4 | Cyr61 | -0.891543 | 0.0003 | 0.0447795 |
| ENSMUSG00000029231.15 | Pdgfra | -1.27891 | 0.00005 | 0.0114713 |
| ENSMUSG00000029371.7 | Cxcl5 | -1.28466 | 0.00005 | 0.0114713 |
| ENSMUSG00000029380.11 | Cxcl1 | -0.820937 | 0.00005 | 0.0114713 |
| ENSMUSG00000029581.14 | Fscn1 | -0.974756 | 0.00005 | 0.0114713 |
| ENSMUSG00000029661.16 | Col1a2 | -1.30902 | 0.00005 | 0.0114713 |
| ENSMUSG00000030022.14 | Adamts9 | -1.41696 | 0.00005 | 0.0114713 |
| ENSMUSG00000030117.5 | Gdf3 | 1.78044 | 0.00005 | 0.0114713 |
| ENSMUSG00000031375.17 | Bgn | -0.85879 | 0.00015 | 0.0265479 |
| ENSMUSG00000031380.10 | Figf | -1.48628 | 0.0002 | 0.0321792 |
| ENSMUSG00000031517.8 | Gpm6a | -0.853333 | 0.00035 | 0.0498408 |
| ENSMUSG00000031673.6 | Cdh11 | -1.06729 | 0.00005 | 0.0114713 |
| ENSMUSG00000031877.8 | Ces2g | -1.18611 | 0.0002 | 0.0321792 |
| ENSMUSG00000032216.14 | Nedd4 | -0.875488 | 0.00005 | 0.0114713 |
| ENSMUSG00000032372.14 | Plscr2 | -1.19169 | 0.0001 | 0.0196651 |
| ENSMUSG00000034205.16 | Loxl2 | -1.32813 | 0.00005 | 0.0114713 |
| ENSMUSG00000034573.14 | Ptpn13 | -1.1545 | 0.00005 | 0.0114713 |
| ENSMUSG00000035202.7 | Lars2 | -0.826839 | 0.00015 | 0.0265479 |
| ENSMUSG00000035373.2 | Ccl7 | -0.837967 | 0.00015 | 0.0265479 |
| ENSMUSG00000035595.6 | 1600002K03Rik | 0.613669 | 0.0003 | 0.0447795 |
| ENSMUSG00000035783.8 | Acta2 | -0.804877 | 0.00005 | 0.0114713 |
| ENSMUSG00000036902.10 | Neto2 | -0.838181 | 0.0001 | 0.0196651 |
| ENSMUSG00000037669.14 | Ldah | -0.581416 | 0.0001 | 0.0196651 |

| | | | | |
|---|---|---|---|---|
| ENSMUSG00000038418.7 | Egr1 | -0.880716 | 0.0002 | 0.0321792 |
| ENSMUSG00000039001.12 | Rps21 | 0.565696 | 0.0003 | 0.0447795 |
| ENSMUSG00000039221.10 | Rpl22l1 | 0.656477 | 0.00005 | 0.0114713 |
| ENSMUSG00000039252.11 | Lgi2 | -1.28286 | 0.0001 | 0.0196651 |
| ENSMUSG00000039831.16 | Arhgap29 | -1.00632 | 0.00005 | 0.0114713 |
| ENSMUSG00000040152.8 | Thbs1 | -0.587178 | 0.0002 | 0.0321792 |
| ENSMUSG00000042985.7 | Upk3b | -1.27702 | 0.00005 | 0.0114713 |
| ENSMUSG00000043110.2 | Lrrn4 | -0.992193 | 0.0002 | 0.0321792 |
| ENSMUSG00000045954.7 | Sdpr | -1.13972 | 0.00005 | 0.0114713 |
| ENSMUSG00000047907.11 | Tshz2 | -1.49576 | 0.00005 | 0.0114713 |
| ENSMUSG00000048376.5 | F2r | -1.11748 | 0.00005 | 0.0114713 |
| ENSMUSG00000049521.1 | Cdc42ep1 | -1.54031 | 0.0003 | 0.0447795 |
| ENSMUSG00000050953.9 | Gja1 | -1.04909 | 0.00015 | 0.0265479 |
| ENSMUSG00000052911.9 | Lamb2 | -2.46259 | 0.0003 | 0.0447795 |
| ENSMUSG00000052957.7 | Gas1 | -0.896758 | 0.00005 | 0.0114713 |
| ENSMUSG00000054793.8 | Cadm4 | -0.863708 | 0.00015 | 0.0265479 |
| ENSMUSG00000061048.7 | Cdh3 | -1.48003 | 0.00005 | 0.0114713 |
| ENSMUSG00000061353.11 | Cxcl12 | -0.945077 | 0.00005 | 0.0114713 |
| ENSMUSG00000065037.1 | Rn7sk | -1.26051 | 0.0001 | 0.0196651 |
| ENSMUSG00000068240.5 | Gm11808 | 0.654087 | 0.00005 | 0.0114713 |
| ENSMUSG00000070436.12 | Serpinh1 | -0.83167 | 0.00005 | 0.0114713 |
| ENSMUSG00000071984.10 | Fndc1 | -2.45468 | 0.0001 | 0.0196651 |
| ENSMUSG00000075254.11 | Heg1 | -0.92478 | 0.00005 | 0.0114713 |
| ENSMUSG00000097971.3 | Gm26917 | -0.998563 | 0.00005 | 0.0114713 |
| ENSMUSG00000100658.1 | F730311O21Rik | -0.761817 | 0.00035 | 0.0498408 |
| ENSMUSG00000110386.1 | RP23-359B23.11 | 0.953329 | 0.00005 | 0.0114713 |

**Table S7. Genes with expression proportional to LDAH levels.**

| Approved symbol | Approved name |
|---|---|
| Serpinf1 | serpin family F member 1 |
| Col1A1 | collagen type I alpha 1 chain |
| Fscn1 | fascin actin-bundling protein 1 |
| Vegfd | vascular endothelial growth factor D |
| Ldah | lipid droplet associated hydrolase |
| Col6A1 | collagen type VI alpha 1 chain |
| Ltbp2 | latent transforming growth factor beta binding protein 2 |
| Ltbp3 | latent transforming growth factor beta binding protein 3 |
| Lamb1 | laminin subunit beta 1 |
| Bicc1 | BicC family RNA binding protein 1 |
| Csf1 | colony stimulating factor 1 |
| Sparc | secreted protein acidic and cysteine rich |
| Ccn2 | cellular communication network factor 2 |
| Col6A2 | collagen type VI alpha 2 chain |
| Igfbp3 | insulin like growth factor binding protein 3 |
| Aebp1 | AE binding protein 1 |
| Ogn | osteoglycin |
| Bmp1 | bone morphogenetic protein 1 |
| Cdh2 | cadherin 2 |
| Col5A2 | collagen type V alpha 2 chain |
| Il1R1 | interleukin 1 receptor type 1 |
| Des | desmin |
| Cd55 | CD55 molecule (Cromer blood group) |
| Meis2 | Meis homeobox 2 |
| Hdc | histidine decarboxylase |
| Heca | hdc homolog, cell cycle regulator |
| Dclk1 | doublecortin like kinase 1 |
| Ccn1 | cellular communication network factor 1 |
| Pdgfra | platelet derived growth factor receptor alpha |
| Cxcl1 | C-X-C motif chemokine ligand 1 |
| Col1A2 | collagen type I alpha 2 chain |
| Bgn | biglycan |
| Plscr2 | phospholipid scramblase 2 |
| Loxl2 | lysyl oxidase like 2 |
| Ptpn13 | protein tyrosine phosphatase non-receptor type 13 |
| Ccl7 | C-C motif chemokine ligand 7 |
| Acta2 | actin alpha 2, smooth muscle |
| Lgi2 | leucine rich repeat LGI family member 2 |
| Arhgap29 | Rho GTPase activating protein 29 |
| Upk3B | uroplakin 3B |

| | |
|---|---|
| F2R | coagulation factor II thrombin receptor |
| Cdc42Ep1 | CDC42 effector protein 1 |
| Gas1 | growth arrest specific 1 |
| Cadm4 | cell adhesion molecule 4 |
| Cdh3 | cadherin 3 |
| Cdh15 | cadherin 15 |
| Cxcl12 | C-X-C motif chemokine ligand 12 |
| Serpinh1 | serpin family H member 1 |
| Fndc1 | fibronectin type III domain containing 1 |
| Mx1 | MX dynamin like GTPase 1 |
| Tom1L2 | target of myb1 like 2 membrane trafficking protein |
| Mapk7 | mitogen-activated protein kinase 7 |
| Ocrl | OCRL inositol polyphosphate-5-phosphatase |
| Gramd1A | GRAM domain containing 1A |
| Slc1A5 | solute carrier family 1 member 5 |
| Kmt2A | lysine methyltransferase 2A |
| Dyrk1B | dual specificity tyrosine phosphorylation regulated kinase 1B |
| Dennd1C | DENN domain containing 1C |
| Nfat5 | nuclear factor of activated T cells 5 |
| Kifc2 | kinesin family member C2 |
| Pan2 | poly(A) specific ribonuclease subunit PAN2 |
| Nlrp4 | NLR family pyrin domain containing 4 |
| Rdh14 | retinol dehydrogenase 14 |
| Hnrnph1 | heterogeneous nuclear ribonucleoprotein H1 |
| Fnbp4 | formin binding protein 4 |
| Dqx1 | DEAQ-box RNA dependent ATPase 1 |
| Ubp1 | upstream binding protein 1 |
| Rbm25 | RNA binding motif protein 25 |
| Cfap161 | cilia and flagella associated protein 161 |
| Ptprs | protein tyrosine phosphatase receptor type S |
| Slc9A5 | solute carrier family 9 member A5 |
| Gdi1 | GDP dissociation inhibitor 1 |
| Dennd6B | DENN domain containing 6B |
| Kdm6B | lysine demethylase 6B |
| Tada2A | transcriptional adaptor 2A |
| Syne4 | spectrin repeat containing nuclear envelope family member 4 |
| Rmnd1 | required for meiotic nuclear division 1 homolog |
| Arg1 | arginase 1 |
| Tinagl1 | tubulointerstitial nephritis antigen like 1 |
| Clk4 | CDC like kinase 4 |
| Limk2 | LIM domain kinase 2 |
| Polm | DNA polymerase mu |

| | |
|---|---|
| Unk | unk zinc finger |
| Rhbdf2 | rhomboid 5 homolog 2 |
| Ntn1 | netrin 1 |
| Pnn | pinin, desmosome associated protein |
| Meg3 | maternally expressed 3 |
| Fam193B | family with sequence similarity 193 member B |
| Mtx3 | metaxin 3 |
| Mss51 | MSS51 mitochondrial translational activator |
| Zswim8 | zinc finger SWIM-type containing 8 |
| Camk2G | calcium/calmodulin dependent protein kinase II gamma |
| Nisch | nischarin |
| Fam160B2 | family with sequence similarity 160 member B2 |
| Acin1 | apoptotic chromatin condensation inducer 1 |
| Zscan26 | zinc finger and SCAN domain containing 26 |
| Trio | trio Rho guanine nucleotide exchange factor |
| Pde1B | phosphodiesterase 1B |
| Paxbp1 | PAX3 and PAX7 binding protein 1 |
| Prpf40B | pre-mRNA processing factor 40 homolog B |
| Celsr3 | cadherin EGF LAG seven-pass G-type receptor 3 |
| Slc25A27 | solute carrier family 25 member 27 |
| Treml1 | triggering receptor expressed on myeloid cells like 1 |
| Akap8 | A-kinase anchoring protein 8 |
| Luc7L | LUC7 like |
| Adamts10 | ADAM metallopeptidase with thrombospondin type 1 motif 10 |
| Trim26 | tripartite motif containing 26 |
| Pcyt2 | phosphate cytidylyltransferase 2, ethanolamine |
| Tepsin | TEPSIN adaptor related protein complex 4 accessory protein |
| Pidd1 | p53-induced death domain protein 1 |
| Ccnt2 | cyclin T2 |
| Elk4 | ETS transcription factor ELK4 |
| Sapcd2 | suppressor APC domain containing 2 |
| Arrdc1 | arrestin domain containing 1 |
| Mdk | midkine |
| Dll4 | delta like canonical Notch ligand 4 |
| Adam33 | ADAM metallopeptidase domain 33 |
| Rbck1 | RANBP2-type and C3HC4-type zinc finger containing 1 |
| Stx16 | syntaxin 16 |
| Helz2 | helicase with zinc finger 2 |
| Alpk1 | alpha kinase 1 |
| Riiad1 | regulatory subunit of type II PKA R-subunit (RIIa) domain containing 1 |
| Tpm2 | tropomyosin 2 |
| Tekt2 | tektin 2 |

| | |
|---|---|
| Miip | migration and invasion inhibitory protein |
| Luc7L2 | LUC7 like 2, pre-mRNA splicing factor |
| Fbxl19 | F-box and leucine rich repeat protein 19 |
| Wdr13 | WD repeat domain 13 |
| Phka2 | phosphorylase kinase regulatory subunit alpha 2 |
| Rfx1 | regulatory factor X1 |
| Mast3 | microtubule associated serine/threonine kinase 3 |
| Lpar2 | lysophosphatidic acid receptor 2 |
| Terf2 | telomeric repeat binding factor 2 |
| Trim29 | tripartite motif containing 29 |
| Pde4A | phosphodiesterase 4A |
| Dock6 | dedicator of cytokinesis 6 |
| Sltm | SAFB like transcription modulator |
| Hmg20A | high mobility group 20A |
| Bsn | bassoon presynaptic cytomatrix protein |
| Amt | aminomethyltransferase |
| Srek1 | splicing regulatory glutamic acid and lysine rich protein 1 |
| Oas2 | 2'-5'-oligoadenylate synthetase 2 |
| Stard9 | StAR related lipid transfer domain containing 9 |
| Mtss2 | MTSS I-BAR domain containing 2 |
| Ppp1R16A | protein phosphatase 1 regulatory subunit 16A |
| Cpsf1 | cleavage and polyadenylation specific factor 1 |
| Cipc | CLOCK interacting pacemaker |
| Ogt | O-linked N-acetylglucosamine (GlcNAc) transferase |
| Slc25A37 | solute carrier family 25 member 37 |
| Znf2 | zinc finger protein 2 |
| Cpsf7 | cleavage and polyadenylation specific factor 7 |
| Dhx57 | DExH-box helicase 57 |
| Mid1 | midline 1 |
| Leng8 | leukocyte receptor cluster member 8 |
| Prpf39 | pre-mRNA processing factor 39 |
| Zcchc7 | zinc finger CCHC-type containing 7 |
| Arhgap45 | Rho GTPase activating protein 45 |
| Usp35 | ubiquitin specific peptidase 35 |
| Bbs10 | Bardet-Biedl syndrome 10 |
| Wnt4 | Wnt family member 4 |
| Ehmt1 | euchromatic histone lysine methyltransferase 1 |
| Stag3 | stromal antigen 3 |
| Znf692 | zinc finger protein 692 |
| Uvssa | UV stimulated scaffold protein A |
| Cep85 | centrosomal protein 85 |
| Thoc2 | THO complex 2 |

| | |
|---|---|
| Ankrd13B | ankyrin repeat domain 13B |
| Zc3H7A | zinc finger CCCH-type containing 7A |
| Wnk2 | WNK lysine deficient protein kinase 2 |
| Mlxip | MLX interacting protein |
| Sgsm2 | small G protein signaling modulator 2 |
| Scaf1 | SR-related CTD associated factor 1 |
| Ubn2 | ubinuclein 2 |
| Ralgps1 | Ral GEF with PH domain and SH3 binding motif 1 |
| Zmym5 | zinc finger MYM-type containing 5 |
| Camta2 | calmodulin binding transcription activator 2 |
| Tet2 | tet methylcytosine dioxygenase 2 |
| Zmiz2 | zinc finger MIZ-type containing 2 |
| Yeats2 | YEATS domain containing 2 |
| Chd7 | chromodomain helicase DNA binding protein 7 |
| Dmxl2 | Dmx like 2 |
| Kif21B | kinesin family member 21B |
| Grk3 | G protein-coupled receptor kinase 3 |
| Ikbke | inhibitor of nuclear factor kappa B kinase subunit epsilon |
| Dennd4B | DENN domain containing 4B |
| Ago4 | argonaute RISC component 4 |
| Asxl1 | ASXL transcriptional regulator 1 |
| Safb2 | scaffold attachment factor B2 |
| Als2Cl | ALS2 C-terminal like |
| Slc38A6 | solute carrier family 38 member 6 |
| Shb | SH2 domain containing adaptor protein B |
| Tmem86B | transmembrane protein 86B |
| Trim39 | tripartite motif containing 39 |
| Npas4 | neuronal PAS domain protein 4 |
| Rbp1 | retinol binding protein 1 |
| Arid4A | AT-rich interaction domain 4A |
| Ankrd16 | ankyrin repeat domain 16 |
| Tmem81 | transmembrane protein 81 |
| Sacs | sacsin molecular chaperone |
| Phldb1 | pleckstrin homology like domain family B member 1 |
| Slc25A47 | solute carrier family 25 member 47 |
| Morn4 | MORN repeat containing 4 |
| Ptges | prostaglandin E synthase |
| Nlgn2 | neuroligin 2 |
| Tchh | trichohyalin |
| Nav2 | neuron navigator 2 |
| Tnrc6A | trinucleotide repeat containing adaptor 6A |
| Usp21 | ubiquitin specific peptidase 21 |

| | |
|---|---|
| Usp25 | ubiquitin specific peptidase 25 |
| Chd8 | chromodomain helicase DNA binding protein 8 |
| Lgals4 | galectin 4 |
| Ercc6 | ERCC excision repair 6, chromatin remodeling factor |
| Gon4L | gon-4 like |
| Znf182 | zinc finger protein 182 |
| Ddx17 | DEAD-box helicase 17 |
| Srsf11 | serine and arginine rich splicing factor 11 |
| Per2 | period circadian regulator 2 |
| Rnf169 | ring finger protein 169 |
| Kmt5C | lysine methyltransferase 5C |
| Zbtb40 | zinc finger and BTB domain containing 40 |
| Clasrp | CLK4 associating serine/arginine rich protein |
| Dot1L | DOT1 like histone lysine methyltransferase |
| Rbak | RB associated KRAB zinc finger |
| Snrnp70 | small nuclear ribonucleoprotein U1 subunit 70 |
| Brpf3 | bromodomain and PHD finger containing 3 |
| Trerf1 | transcriptional regulating factor 1 |
| Fbxl18 | F-box and leucine rich repeat protein 18 |
| Cep152 | centrosomal protein 152 |
| Clk2 | CDC like kinase 2 |
| Clk2 | CDC like kinase 2 |
| Fgfr1Op | FGFR1 oncogene partner |
| Bicra | BRD4 interacting chromatin remodeling complex associated protein |
| Ahnak2 | AHNAK nucleoprotein 2 |
| C4B | complement C4B (Chido blood group) |
| C4A | complement C4A (Rodgers blood group) |
| D2Hgdh | D-2-hydroxyglutarate dehydrogenase |
| Sptbn5 | spectrin beta, non-erythrocytic 5 |
| Zc3H3 | zinc finger CCCH-type containing 3 |
| Plekhn1 | pleckstrin homology domain containing N1 |
| Spaca6 | sperm acrosome associated 6 |
| Snhg15 | small nucleolar RNA host gene 15 |
| Snhg20 | small nucleolar RNA host gene 20 |
| Mir17Hg | miR-17-92a-1 cluster host gene |
| Neat1 | nuclear paraspeckle assembly transcript 1 |
| Malat1 | metastasis associated lung adenocarcinoma transcript 1 |
| Meg8 | maternally expressed 8, small nucleolar RNA host gene |
| Ms4A14 | membrane spanning 4-domains A14 |
| Kcnq1Ot1 | KCNQ1 opposite strand/antisense transcript 1 |

**Table S8. Overlapping genes between Ldah and previously identified LXR targets in macrophages.**

| | | Goo et al | Feldman et al. | Feldman et al. | Feldman et al. | Feldman et al. | Ramon-Vazquez et al. |
|---|---|---|---|---|---|---|---|
| | | | Fold vs. THP-1 + DMSO | | | Fold vs. THP-1 foam cells | BMM |
| | | Foam cells | THP-1 + T0901317 | THP-1 foam cells + T0901317 | THP-1 foam cells | THP-1 foam cell + T0901317 | GW 3965 |
| gene_id | symbol | Log2 FC (Tg/WT) | Fold Change | Fold Change | Fold Change | Fold Change | LXR target |
| ENSMUSG00000031098.18 | Syt8 | 1.23291 | - | - | - | - | Yes |
| ENSMUSG00000066975.12 | Cryba4 | 0.848567 | - | - | - | - | Yes |
| ENSMUSG00000002100.15 | Mybpc3 | 0.801451 | - | - | 3.55 | - | - |
| ENSMUSG00000045282.7 | Tmem86b | 0.680165 | - | 2.08 | - | - | - |
| ENSMUSG00000027239.14 | Mdk | 0.680099 | - | - | - | - | Yes |
| ENSMUSG00000040663.8 | Clcf1 | 0.579513 | - | - | 4.32 | - | - |
| ENSMUSG00000073609.9 | D2hgdh | 0.57739 | - | 2.15 | 2.28 | - | - |
| ENSMUSG00000008200.14 | Fnbp4 | 0.559785 | - | 2.47 | 1.67 | - | - |
| ENSMUSG00000032198.8 | Dock6 | 0.552541 | - | 6.55 | 3.47* | - | - |
| ENSMUSG00000050737.13 | Ptges | 0.488789 | - | 7.91 | 10.45 | - | - |
| ENSMUSG00000021910.15 | Nisch | 0.476426 | 0.76 | - | - | - | - |
| ENSMUSG00000031389.17 | Arhgap4 | 0.471822 | 0.77 | 0.57 | - | - | - |
| ENSMUSG00000044813.15 | Shb | 0.467815 | - | 3.04 | 1.87 | - | Yes |
| ENSMUSG00000055866.9 | Per2 | 0.464561 | - | - | - | - | Yes |
| ENSMUSG00000009741.14 | Ubp1 | 0.433344 | 1.28 | - | - | - | - |
| ENSMUSG00000006395.16 | Hyi | 0.429045 | - | 2.23 | 1.97 | - | - |
| ENSMUSG00000023952.13 | Gtpbp2 | 0.41292 | - | 3.84 | - | - | - |
| ENSMUSG00000041235.11 | Chd7 | 0.404886 | 1.32 | - | - | - | - |
| ENSMUSG00000031295.13 | Phka2 | 0.393297 | - | - | 3.36 | - | - |
| ENSMUSG00000001173.15 | Ocrl | 0.391173 | - | - | 2.14 | - | - |
| ENSMUSG00000033819.15 | Ppp1r16a | 0.390599 | - | - | 4.45* | - | - |
| ENSMUSG00000042349.13 | Ikbke | 0.378942 | - | - | 0.51 | - | Yes |
| ENSMUSG00000024193.7 | Phf1 | 0.359026 | - | 5.15 | 2.32 | 2.48 | - |
| ENSMUSG00000038351.14 | Sgsm2 | 0.329184 | - | - | 1.95 | - | - |
| ENSMUSG00000025158.7 | Rfng | 0.324318 | - | - | 1.9 | - | - |
| ENSMUSG00000037221.13 | Mospd3 | 0.31899 | - | - | 0.42 | - | - |
| ENSMUSG00000026544.6 | Dusp23 | 0.29605 | - | 0.59 | - | 0.56 | - |
| ENSMUSG00000001918.17 | Slc1a5 | 0.292262 | - | 5.03 | 5.39* | - | - |
| ENSMUSG00000029817.11 | Tra2a | 0.291 | - | - | 0.36 | - | - |
| ENSMUSG00000025144.17 | Stra13 | 0.288484 | - | 0.51* | 0.57* | - | - |
| ENSMUSG00000024856.10 | Cdk2ap2 | 0.287504 | - | 0.46* | 0.42 | - | - |
| ENSMUSG00000041781.8 | Cpsf2 | -0.260727 | - | - | 1.68 | - | - |
| ENSMUSG00000041958.10 | Pigs | -0.262644 | - | - | 0.5 | - | - |
| ENSMUSG00000027775.9 | Mfsd1 | -0.26537 | - | 0.29 | 0.33 | - | - |
| ENSMUSG00000050912.15 | Tmem123 | -0.269675 | 1.32* | - | - | - | - |
| ENSMUSG00000022912.8 | Pros1 | -0.270499 | - | - | 0.57* | - | - |
| ENSMUSG00000046841.4 | Ckap4 | -0.273775 | 1.26 | 2.54* | 1.97 | - | - |
| ENSMUSG00000031165.6 | Was | -0.274809 | - | 0.55* | 0.46* | - | - |
| ENSMUSG00000021108.17 | Prkch | -0.275429 | - | 4.98* | 3.63* | - | - |
| ENSMUSG00000031337.16 | Mtm1 | -0.277104 | - | - | - | - | Yes |
| ENSMUSG00000020075.7 | Ddx21 | -0.279205 | 1.47* | - | - | - | - |

| | | | | | | | |
|---|---|---|---|---|---|---|---|
| ENSMUSG00000011179.7 | Odc1 | -0.289525 | - | 0.46 | 0.55 | - | - |
| ENSMUSG00000032763.10 | IlYesbl | -0.290879 | - | - | - | - | Yes |
| ENSMUSG00000030220.13 | Arhgdib | -0.290993 | - | 0.24 | 0.3 | - | - |
| ENSMUSG00000032349.13 | EloYesl5 | -0.29941 | 1.97* | - | - | - | Yes |
| ENSMUSG00000026223.15 | Itm2c | -0.301775 | 0.8 | - | 0.53 | - | - |
| ENSMUSG00000068798.10 | Rap1a | -0.30521 | - | - | - | - | Yes |
| ENSMUSG00000049516.9 | Spty2d1 | -0.310043 | - | 0.38 | 0.39 | - | - |
| ENSMUSG00000029998.14 | Pcyox1 | -0.311046 | - | - | - | - | Yes |
| ENSMUSG00000032050.16 | Rdx | -0.311564 | - | 2.34 | - | - | - |
| ENSMUSG00000020841.5 | Cpd | -0.313264 | - | 2.18* | 2.21 | - | - |
| ENSMUSG00000022664.11 | Slc35a5 | -0.324119 | - | 0.53 | - | - | - |
| ENSMUSG00000029338.13 | Antxr2 | -0.324286 | - | 0.2 | 0.29 | - | - |
| ENSMUSG00000020130.8 | Tbc1d15 | -0.324594 | - | 2.32* | - | - | - |
| ENSMUSG00000079037.9 | Prnp | -0.329665 | 1.33 | 3.29* | 2.00* | - | - |
| ENSMUSG00000025203.5 | Scd2 | -0.332794 | - | - | - | - | Yes |
| ENSMUSG00000027195.10 | Hsd17b12 | -0.337639 | - | - | 1.84 | - | - |
| ENSMUSG00000035847.15 | Ids | -0.341298 | - | 0.51* | 0.47* | - | - |
| ENSMUSG00000029672.16 | Fam3c | -0.352147 | - | - | - | - | Yes |
| ENSMUSG00000003721.14 | Insig2 | -0.35411 | - | - | 0.52* | - | - |
| ENSMUSG00000031007.2 | Atp6ap2 | -0.3555 | - | 0.52 | 0.46 | - | - |
| ENSMUSG00000032475.15 | Nck1 | -0.355867 | - | 0.42* | 0.46* | - | - |
| ENSMUSG00000029426.8 | Scarb2 | -0.364799 | - | - | - | - | Yes |
| ENSMUSG00000033863.1 | Klf9 | -0.368354 | - | 2.11* | 2.21* | - | - |
| ENSMUSG00000020048.13 | Hsp90b1 | -0.371044 | - | 0.50* | 0.4 | - | - |
| ENSMUSG00000062070.12 | Pgk1 | -0.373352 | - | 0.41 | 0.53 | - | - |
| ENSMUSG00000037071.2 | Scd1 | -0.37379 | - | - | - | - | Yes |
| ENSMUSG00000022257.3 | Laptm4b | -0.374818 | - | - | 0.47* | - | - |
| ENSMUSG00000015854.7 | Cd5l | -0.375955 | - | - | - | - | Yes |
| ENSMUSG00000029767.16 | Calu | -0.380567 | - | 2.00* | - | - | - |
| ENSMUSG00000072235.5 | Tuba1a | -0.38428 | - | 0.3 | 0.38 | - | - |
| ENSMUSG00000030786.18 | Itgam | -0.384339 | - | 0.38 | 0.38 | - | - |
| ENSMUSG00000030208.15 | Emp1 | -0.386647 | - | - | 1.93 | - | - |
| ENSMUSG00000017057.9 | Il13ra1 | -0.39076 | - | - | 0.45 | - | - |
| ENSMUSG00000030096.8 | Slc6a6 | -0.394469 | - | 4.25* | 3.93 | - | - |
| ENSMUSG00000028111.4 | Ctsk | -0.397706 | - | 3.73 | - | - | - |
| ENSMUSG00000071855.4 | Ccdc112 | -0.400304 | - | 0.35 | - | - | - |
| ENSMUSG00000078945.10 | Naip2 | -0.401369 | - | - | - | - | Yes |
| ENSMUSG00000021190.14 | Lgmn | -0.404262 | 0.72* | 0.19* | - | - | - |
| ENSMUSG00000045092.8 | S1pr1 | -0.436563 | - | - | - | - | Yes |
| ENSMUSG00000020571.12 | Pdia6 | -0.441169 | - | 0.37 | 0.4 | - | - |
| ENSMUSG00000026986.14 | Hnmt | -0.45104 | - | 0.74 | - | - | - |
| ENSMUSG00000026864.13 | Hspa5 | -0.45858 | - | - | 0.56* | - | - |
| ENSMUSG00000028270.12 | Gbp2 | -0.460988 | - | - | 0.17 | 5.85* | - |
| ENSMUSG00000042684.8 | Npl | -0.469555 | 0.72 | 0.54 | - | - | - |
| ENSMUSG00000022351.14 | Sqle | -0.471421 | 1.59 | 0.38 | 0.25 | - | - |
| ENSMUSG00000053253.16 | Ndfip2 | -0.471635 | - | 0.58* | 0.52 | - | - |
| ENSMUSG00000020368.15 | Canx | -0.481378 | - | - | 0.47 | - | - |
| ENSMUSG00000025016.10 | Tm9sf3 | -0.489441 | - | - | 0.51 | - | - |

| | | | | | | | |
|---|---|---|---|---|---|---|---|
| ENSMUSG00000028563.16 | Tm2d1 | -0.537236 | - | - | 1.85* | - | - |
| ENSMUSG00000019872.13 | Smpdl3a | -0.569659 | 4.03 | - | - | - | - |
| ENSMUSG00000023845.6 | Lnpep | -0.593034 | - | - | - | - | Yes |
| ENSMUSG00000002944.15 | Cd36 | -0.629375 | 1.27 | - | 1.84 | - | - |
| ENSMUSG00000060703.12 | Cd302 | -0.701273 | - | - | - | - | Yes |
| ENSMUSG00000014905.3 | Dnajb9 | -0.744725 | - | 2.63 | - | 2.02 | - |
| ENSMUSG00000067714.13 | Lpar5 | -0.84304 | - | - | 0.14 | - | - |
| ENSMUSG00000079625.2 | Tm4sf19 | -0.862598 | 1.37 | - | - | - | - |
| ENSMUSG00000079685.9 | Ulbp1 | -0.895772 | - | 3.35* | 3.53 | - | - |

* : LXR knockdown sensitive

**Table S9.** Two-tailed Pearson's correlation of LDAH with other genes in arterial leukocytes.

| Feature ID | P-value (Ldah) | FDR step up (Ldah) | Partial correlation (Ldah) |
|---|---|---|---|
| Syngr1 | 1.34E-17 | 8.83E-14 | 1.26E-01 |
| Tmem256 | 1.65E-17 | 8.83E-14 | 1.25E-01 |
| Mfge8 | 1.12E-16 | 4.47E-13 | 1.22E-01 |
| Mlf2 | 1.45E-16 | 4.64E-13 | 1.22E-01 |
| 2010111I01Rik | 2.22E-16 | 5.93E-13 | 1.21E-01 |
| Atp6v0d2 | 3.06E-16 | 7.01E-13 | 1.20E-01 |
| Clu | 4.02E-16 | 7.62E-13 | 1.20E-01 |
| Degs1 | 4.28E-16 | 7.62E-13 | 1.20E-01 |
| Lum | 7.61E-16 | 1.22E-12 | 1.19E-01 |
| Acta2 | 1.17E-15 | 1.70E-12 | 1.18E-01 |
| Nr1h3 | 1.50E-15 | 1.92E-12 | 1.17E-01 |
| Gpnmb | 1.64E-15 | 1.92E-12 | 1.17E-01 |
| Uap1l1 | 1.67E-15 | 1.92E-12 | 1.17E-01 |
| Rcan2 | 2.53E-15 | 2.70E-12 | 1.16E-01 |
| Lrrc58 | 2.71E-15 | 2.71E-12 | 1.16E-01 |
| Amfr | 5.47E-15 | 5.16E-12 | 1.15E-01 |
| Cmas | 6.42E-15 | 5.71E-12 | 1.15E-01 |
| Polr2l | 6.83E-15 | 5.76E-12 | 1.15E-01 |
| Ptprb | 7.87E-15 | 6.30E-12 | 1.14E-01 |
| Morf4l2 | 1.01E-14 | 7.22E-12 | 1.14E-01 |
| Spcs1 | 1.04E-14 | 7.22E-12 | 1.14E-01 |
| Igf2r | 1.25E-14 | 8.35E-12 | 1.13E-01 |
| Vat1 | 1.41E-14 | 9.06E-12 | 1.13E-01 |
| Igfbp7 | 1.50E-14 | 9.21E-12 | 1.13E-01 |
| Gyg | 1.89E-14 | 1.12E-11 | 1.13E-01 |
| Ahnak2 | 2.59E-14 | 1.40E-11 | 1.12E-01 |
| Acads | 2.62E-14 | 1.40E-11 | 1.12E-01 |
| Mgp | 3.61E-14 | 1.85E-11 | 1.11E-01 |
| Oxct1 | 3.69E-14 | 1.85E-11 | 1.11E-01 |
| Mmp12 | 4.10E-14 | 1.99E-11 | 1.11E-01 |
| Nagk | 4.51E-14 | 2.13E-11 | 1.11E-01 |
| Fam198b | 4.71E-14 | 2.15E-11 | 1.11E-01 |
| Mrpl18 | 5.05E-14 | 2.24E-11 | 1.11E-01 |
| Il7r | 6.06E-14 | 2.62E-11 | 1.10E-01 |
| Cd5l | 6.43E-14 | 2.64E-11 | 1.10E-01 |
| Mrpl27 | 9.58E-14 | 3.83E-11 | 1.10E-01 |
| Hebp1 | 1.10E-13 | 4.19E-11 | 1.09E-01 |
| Fabp5 | 1.14E-13 | 4.21E-11 | 1.09E-01 |
| Nkiras2 | 1.16E-13 | 4.21E-11 | 1.09E-01 |
| Myl9 | 1.35E-13 | 4.79E-11 | 1.09E-01 |
| Tagln | 1.66E-13 | 5.78E-11 | 1.09E-01 |
| Tm4sf1 | 2.13E-13 | 7.16E-11 | 1.08E-01 |

| | | | |
|---|---|---|---|
| Myh11 | 2.15E-13 | 7.16E-11 | 1.08E-01 |
| Anxa4 | 2.31E-13 | 7.56E-11 | 1.08E-01 |
| Bgn | 2.76E-13 | 8.84E-11 | 1.08E-01 |
| Olfml3 | 2.86E-13 | 8.98E-11 | 1.07E-01 |
| Col1a2 | 3.61E-13 | 1.11E-10 | 1.07E-01 |
| Fam129b | 3.83E-13 | 1.16E-10 | 1.07E-01 |
| Rnf7 | 3.92E-13 | 1.16E-10 | 1.07E-01 |
| Higd1a | 4.30E-13 | 1.25E-10 | 1.07E-01 |
| Pam | 4.97E-13 | 1.41E-10 | 1.06E-01 |
| Cops7a | 5.01E-13 | 1.41E-10 | 1.06E-01 |
| N6amt2 | 5.29E-13 | 1.46E-10 | 1.06E-01 |
| Sparc | 6.23E-13 | 1.69E-10 | 1.06E-01 |
| Ctsd | 6.45E-13 | 1.72E-10 | 1.06E-01 |
| Fblim1 | 6.54E-13 | 1.72E-10 | 1.06E-01 |
| Lpl | 8.28E-13 | 2.13E-10 | 1.05E-01 |
| Mrps23 | 8.39E-13 | 2.13E-10 | 1.05E-01 |
| Gng12 | 8.82E-13 | 2.21E-10 | 1.05E-01 |
| Rab1a | 9.70E-13 | 2.39E-10 | 1.05E-01 |
| Dusp3 | 1.00E-12 | 2.43E-10 | 1.05E-01 |
| Csrp2 | 1.02E-12 | 2.43E-10 | 1.05E-01 |
| Trem2 | 1.06E-12 | 2.49E-10 | 1.05E-01 |
| Col3a1 | 1.14E-12 | 2.65E-10 | 1.05E-01 |
| Tmed3 | 1.24E-12 | 2.84E-10 | 1.05E-01 |
| Cyb5r3 | 1.37E-12 | 3.09E-10 | 1.04E-01 |
| Chmp3 | 1.45E-12 | 3.23E-10 | 1.04E-01 |
| Tpm2 | 1.56E-12 | 3.42E-10 | 1.04E-01 |
| Psmd6 | 1.65E-12 | 3.58E-10 | 1.04E-01 |
| Tpm1 | 1.71E-12 | 3.64E-10 | 1.04E-01 |
| Tmem147 | 1.85E-12 | 3.89E-10 | 1.04E-01 |
| Wbp5 | 1.93E-12 | 4.01E-10 | 1.04E-01 |
| Dnajc19 | 2.17E-12 | 4.45E-10 | 1.03E-01 |
| Man1a2 | 2.25E-12 | 4.57E-10 | 1.03E-01 |
| Pld3 | 2.29E-12 | 4.58E-10 | 1.03E-01 |
| Cltb | 2.34E-12 | 4.58E-10 | 1.03E-01 |
| Speg | 2.37E-12 | 4.58E-10 | 1.03E-01 |
| Arl2 | 2.39E-12 | 4.58E-10 | 1.03E-01 |
| Atp5f1 | 2.40E-12 | 4.58E-10 | 1.03E-01 |
| Slc38a7 | 2.43E-12 | 4.58E-10 | 1.03E-01 |
| Napa | 2.87E-12 | 5.29E-10 | 1.03E-01 |
| Eln | 2.88E-12 | 5.29E-10 | 1.03E-01 |
| Elovl1 | 2.97E-12 | 5.41E-10 | 1.03E-01 |
| Cnn1 | 3.14E-12 | 5.64E-10 | 1.03E-01 |
| Sucla2 | 3.23E-12 | 5.75E-10 | 1.03E-01 |
| Gtf3a | 3.42E-12 | 6.02E-10 | 1.02E-01 |

| | | | |
|---|---|---|---|
| Nrp2 | 3.66E-12 | 6.28E-10 | 1.02E-01 |
| Pcp4l1 | 3.67E-12 | 6.28E-10 | 1.02E-01 |
| Capg | 3.72E-12 | 6.28E-10 | 1.02E-01 |
| Itgav | 3.88E-12 | 6.47E-10 | 1.02E-01 |
| Gsk3b | 4.05E-12 | 6.69E-10 | 1.02E-01 |
| Dcn | 4.15E-12 | 6.75E-10 | 1.02E-01 |
| Tnfsf12 | 4.18E-12 | 6.75E-10 | 1.02E-01 |
| Exosc4 | 4.22E-12 | 6.75E-10 | 1.02E-01 |
| Mfsd11 | 4.49E-12 | 7.12E-10 | 1.02E-01 |
| Uggt1 | 4.57E-12 | 7.15E-10 | 1.02E-01 |
| Arl11 | 4.60E-12 | 7.15E-10 | 1.02E-01 |
| Bcar3 | 5.45E-12 | 8.37E-10 | 1.02E-01 |
| Arl8b | 5.49E-12 | 8.37E-10 | 1.01E-01 |
| Mydgf | 5.54E-12 | 8.37E-10 | 1.01E-01 |
| Serpina3n | 5.84E-12 | 8.73E-10 | 1.01E-01 |
| Mcfd2 | 6.52E-12 | 9.67E-10 | 1.01E-01 |
| Tspan4 | 6.92E-12 | 9.99E-10 | 1.01E-01 |
| Mrpl51 | 7.27E-12 | 1.04E-09 | 1.01E-01 |
| Cisd1 | 7.31E-12 | 1.04E-09 | 1.01E-01 |
| Cyc1 | 7.43E-12 | 1.04E-09 | 1.01E-01 |
| Mgat5 | 8.08E-12 | 1.12E-09 | 1.01E-01 |
| Gm10020 | 8.21E-12 | 1.13E-09 | 1.01E-01 |
| Ngfrap1 | 8.32E-12 | 1.14E-09 | 1.01E-01 |
| Gadd45a | 8.46E-12 | 1.15E-09 | 1.01E-01 |
| Spp1 | 8.75E-12 | 1.18E-09 | 1.01E-01 |
| Pgp | 9.69E-12 | 1.29E-09 | 1.00E-01 |
| Ufm1 | 1.03E-11 | 1.36E-09 | 1.00E-01 |
| Slc27a1 | 1.04E-11 | 1.36E-09 | 1.00E-01 |
| Trappc6b | 1.10E-11 | 1.44E-09 | 1.00E-01 |
| Rxra | 1.19E-11 | 1.53E-09 | 9.99E-02 |
| Apoe | 1.21E-11 | 1.55E-09 | -9.98E-02 |
| Plekhb2 | 1.22E-11 | 1.55E-09 | 9.98E-02 |
| Tmem65 | 1.36E-11 | 1.69E-09 | 9.96E-02 |
| Arpc5l | 1.38E-11 | 1.69E-09 | 9.96E-02 |
| Col1a1 | 1.38E-11 | 1.69E-09 | 9.96E-02 |
| Gm4799 | 1.47E-11 | 1.78E-09 | 9.94E-02 |
| Mff | 1.52E-11 | 1.80E-09 | 9.94E-02 |
| Glg1 | 1.52E-11 | 1.80E-09 | 9.93E-02 |
| Gclm | 1.53E-11 | 1.80E-09 | 9.93E-02 |
| Ldlrap1 | 1.53E-11 | 1.80E-09 | 9.93E-02 |
| Scd2 | 1.54E-11 | 1.80E-09 | 9.93E-02 |
| Hacd1 | 1.55E-11 | 1.80E-09 | 9.93E-02 |
| Dtnbp1 | 1.60E-11 | 1.84E-09 | 9.92E-02 |
| Gdpd1 | 1.83E-11 | 2.09E-09 | 9.90E-02 |

| | | | |
|---|---|---|---|
| Vti1b | 2.02E-11 | 2.28E-09 | 9.87E-02 |
| Dap | 2.03E-11 | 2.28E-09 | 9.87E-02 |
| Ctsb | 2.06E-11 | 2.28E-09 | 9.87E-02 |
| Timm13 | 2.08E-11 | 2.28E-09 | 9.87E-02 |
| Nucb1 | 2.09E-11 | 2.28E-09 | 9.87E-02 |
| Ankrd40 | 2.10E-11 | 2.28E-09 | 9.87E-02 |
| Itfg1 | 2.86E-11 | 3.07E-09 | 9.80E-02 |
| Atp6v1a | 3.08E-11 | 3.29E-09 | 9.78E-02 |
| Ak6 | 3.19E-11 | 3.38E-09 | 9.78E-02 |
| Gpr137b | 3.35E-11 | 3.53E-09 | 9.77E-02 |
| Ctsk | 3.89E-11 | 4.05E-09 | 9.73E-02 |
| Emc4 | 3.94E-11 | 4.07E-09 | 9.73E-02 |
| Tmem205 | 3.99E-11 | 4.10E-09 | 9.73E-02 |
| Gstm1 | 4.22E-11 | 4.30E-09 | 9.72E-02 |
| Creg1 | 4.39E-11 | 4.45E-09 | 9.71E-02 |
| Fzd2 | 4.46E-11 | 4.49E-09 | 9.70E-02 |
| Socs6 | 4.82E-11 | 4.82E-09 | 9.69E-02 |
| Mrpl36 | 4.95E-11 | 4.92E-09 | 9.68E-02 |
| Actn1 | 5.02E-11 | 4.96E-09 | 9.68E-02 |
| Sost | 5.07E-11 | 4.98E-09 | 9.68E-02 |
| Minos1 | 5.41E-11 | 5.25E-09 | 9.66E-02 |
| Naa20 | 5.48E-11 | 5.29E-09 | 9.66E-02 |
| Blnk | 5.74E-11 | 5.48E-09 | 9.65E-02 |
| Mrpl40 | 5.75E-11 | 5.48E-09 | 9.65E-02 |
| Gng11 | 5.78E-11 | 5.48E-09 | 9.65E-02 |
| Ykt6 | 6.28E-11 | 5.92E-09 | 9.63E-02 |
| Rnf128 | 6.36E-11 | 5.95E-09 | 9.63E-02 |
| Metap2 | 6.66E-11 | 6.20E-09 | 9.62E-02 |
| Emp1 | 7.25E-11 | 6.71E-09 | 9.60E-02 |
| Serpinb6a | 7.47E-11 | 6.87E-09 | 9.59E-02 |
| Commd9 | 7.58E-11 | 6.94E-09 | 9.59E-02 |
| Sod3 | 7.75E-11 | 7.05E-09 | 9.58E-02 |
| Fn1 | 8.32E-11 | 7.53E-09 | 9.57E-02 |
| Glrx5 | 8.58E-11 | 7.72E-09 | 9.56E-02 |
| Mrps17 | 8.70E-11 | 7.78E-09 | 9.56E-02 |
| Mrpl34 | 9.02E-11 | 7.99E-09 | 9.55E-02 |
| Col4a1 | 9.10E-11 | 8.01E-09 | 9.55E-02 |
| Arl6ip5 | 9.43E-11 | 8.21E-09 | 9.54E-02 |
| Il11ra1 | 9.76E-11 | 8.40E-09 | 9.53E-02 |
| Gusb | 9.94E-11 | 8.52E-09 | 9.53E-02 |
| Sf3b5 | 1.02E-10 | 8.68E-09 | 9.52E-02 |
| Fkrp | 1.03E-10 | 8.71E-09 | 9.52E-02 |
| Vapb | 1.06E-10 | 8.87E-09 | 9.51E-02 |
| Rnf4 | 1.14E-10 | 9.45E-09 | 9.50E-02 |

| | | | |
|---|---|---|---|
| Surf4 | 1.15E-10 | 9.47E-09 | 9.50E-02 |
| Fam162a | 1.15E-10 | 9.48E-09 | 9.49E-02 |
| Bola3 | 1.18E-10 | 9.59E-09 | 9.49E-02 |
| Glmp | 1.18E-10 | 9.59E-09 | 9.49E-02 |
| Fbxo32 | 1.28E-10 | 1.04E-08 | 9.47E-02 |
| Cpe | 1.37E-10 | 1.10E-08 | 9.46E-02 |
| Pdzd11 | 1.41E-10 | 1.13E-08 | 9.45E-02 |
| Tpp1 | 1.44E-10 | 1.14E-08 | 9.45E-02 |
| Tmx2 | 1.50E-10 | 1.18E-08 | 9.44E-02 |
| Pno1 | 1.55E-10 | 1.21E-08 | 9.43E-02 |
| Hist1h1c | 1.62E-10 | 1.26E-08 | 9.42E-02 |
| Psmb5 | 1.66E-10 | 1.28E-08 | 9.41E-02 |
| Psmd12 | 1.67E-10 | 1.28E-08 | 9.41E-02 |
| Ogdh | 1.70E-10 | 1.29E-08 | 9.41E-02 |
| Knop1 | 1.74E-10 | 1.32E-08 | 9.40E-02 |
| Dda1 | 1.78E-10 | 1.34E-08 | 9.40E-02 |
| Mrps34 | 1.83E-10 | 1.38E-08 | 9.39E-02 |
| Lrp1 | 1.89E-10 | 1.41E-08 | 9.38E-02 |
| Myo1c | 1.91E-10 | 1.42E-08 | 9.38E-02 |
| Atic | 1.93E-10 | 1.43E-08 | 9.38E-02 |
| Gpr180 | 2.01E-10 | 1.48E-08 | 9.37E-02 |
| Srp19 | 2.07E-10 | 1.52E-08 | 9.36E-02 |
| Pcnp | 2.11E-10 | 1.53E-08 | 9.36E-02 |
| Cab39 | 2.16E-10 | 1.57E-08 | 9.35E-02 |
| Mrpl17 | 2.21E-10 | 1.59E-08 | 9.35E-02 |
| Atraid | 2.22E-10 | 1.59E-08 | 9.35E-02 |
| Psmd1 | 2.33E-10 | 1.67E-08 | 9.34E-02 |
| Pdha1 | 2.42E-10 | 1.72E-08 | 9.33E-02 |
| Nubp2 | 2.49E-10 | 1.76E-08 | 9.32E-02 |
| Fam20c | 2.50E-10 | 1.76E-08 | 9.32E-02 |
| Pank3 | 2.56E-10 | 1.80E-08 | 9.32E-02 |
| Pgam1 | 2.68E-10 | 1.86E-08 | 9.31E-02 |
| Ssr2 | 2.71E-10 | 1.88E-08 | 9.30E-02 |
| Tmem208 | 2.88E-10 | 1.97E-08 | 9.29E-02 |
| Sep15 | 3.02E-10 | 2.06E-08 | 9.28E-02 |
| Yipf4 | 3.08E-10 | 2.09E-08 | 9.27E-02 |
| Nme6 | 3.09E-10 | 2.09E-08 | 9.27E-02 |
| Wdr83os | 3.24E-10 | 2.17E-08 | 9.26E-02 |
| Mid1 | 3.28E-10 | 2.19E-08 | 9.26E-02 |
| Ube2m | 3.36E-10 | 2.23E-08 | 9.25E-02 |
| Lpcat3 | 3.43E-10 | 2.27E-08 | 9.25E-02 |
| Mrpl44 | 3.46E-10 | 2.28E-08 | 9.25E-02 |
| Rwdd1 | 3.51E-10 | 2.30E-08 | 9.24E-02 |
| Ubxn6 | 3.54E-10 | 2.32E-08 | 9.24E-02 |

| | | | |
|---|---|---|---|
| Dnajc15 | 3.58E-10 | 2.33E-08 | 9.24E-02 |
| Immp1l | 3.60E-10 | 2.33E-08 | 9.24E-02 |
| Rnf5 | 3.65E-10 | 2.36E-08 | 9.23E-02 |
| BC031181 | 3.88E-10 | 2.47E-08 | 9.22E-02 |
| Rab10 | 4.07E-10 | 2.55E-08 | 9.21E-02 |
| Osbpl8 | 4.08E-10 | 2.55E-08 | 9.21E-02 |
| Mgst1 | 4.17E-10 | 2.60E-08 | 9.20E-02 |
| Serpinh1 | 4.22E-10 | 2.62E-08 | 9.20E-02 |
| Apbb2 | 4.32E-10 | 2.65E-08 | 9.20E-02 |
| Ndufa10 | 4.43E-10 | 2.70E-08 | 9.19E-02 |
| Mlec | 4.51E-10 | 2.71E-08 | 9.19E-02 |
| Purb | 4.51E-10 | 2.71E-08 | 9.19E-02 |
| Cyfip1 | 4.61E-10 | 2.75E-08 | 9.18E-02 |
| Wisp2 | 4.63E-10 | 2.76E-08 | 9.18E-02 |
| Timm8b | 4.81E-10 | 2.84E-08 | 9.17E-02 |
| Commd2 | 4.83E-10 | 2.84E-08 | 9.17E-02 |
| Ndufc1 | 4.83E-10 | 2.84E-08 | 9.17E-02 |
| U2af1l4 | 4.92E-10 | 2.89E-08 | 9.17E-02 |
| Mmp3 | 4.99E-10 | 2.91E-08 | 9.16E-02 |
| AI427809 | 5.09E-10 | 2.96E-08 | 9.16E-02 |
| Lipa | 5.16E-10 | 2.98E-08 | 9.16E-02 |
| Srp14 | 5.16E-10 | 2.98E-08 | 9.16E-02 |
| Ghitm | 5.17E-10 | 2.98E-08 | 9.16E-02 |
| Rtn3 | 5.19E-10 | 2.98E-08 | 9.15E-02 |
| Echs1 | 5.20E-10 | 2.98E-08 | 9.15E-02 |
| Atp5g1 | 5.29E-10 | 3.02E-08 | 9.15E-02 |
| Slc6a8 | 5.40E-10 | 3.07E-08 | 9.15E-02 |
| Htr2b | 5.43E-10 | 3.07E-08 | 9.14E-02 |
| Cetn3 | 5.72E-10 | 3.22E-08 | 9.13E-02 |
| Ndufs6 | 5.74E-10 | 3.22E-08 | 9.13E-02 |
| Hypk | 5.80E-10 | 3.25E-08 | 9.13E-02 |
| Dpep2 | 6.05E-10 | 3.38E-08 | 9.12E-02 |
| Pdlim4 | 6.41E-10 | 3.55E-08 | 9.11E-02 |
| Cytl1 | 6.57E-10 | 3.63E-08 | 9.10E-02 |
| Tmem160 | 6.78E-10 | 3.71E-08 | 9.09E-02 |
| Slc25a51 | 6.78E-10 | 3.71E-08 | 9.09E-02 |
| Tmem86a | 6.85E-10 | 3.73E-08 | 9.09E-02 |
| Ctsl | 7.18E-10 | 3.88E-08 | 9.08E-02 |
| Dtymk | 7.21E-10 | 3.88E-08 | 9.08E-02 |
| 1810037I17Rik | 7.98E-10 | 4.26E-08 | 9.05E-02 |
| Pnkd | 8.38E-10 | 4.44E-08 | 9.04E-02 |
| l7Rn6 | 8.61E-10 | 4.55E-08 | 9.04E-02 |
| Znrf1 | 8.72E-10 | 4.59E-08 | 9.03E-02 |
| Gpr137b-ps | 8.80E-10 | 4.62E-08 | 9.03E-02 |

| | | | |
|---|---|---|---|
| Snx15 | 8.88E-10 | 4.65E-08 | 9.03E-02 |
| Smpdl3a | 8.91E-10 | 4.65E-08 | 9.03E-02 |
| Scoc | 8.99E-10 | 4.67E-08 | 9.03E-02 |
| Glb1 | 9.01E-10 | 4.67E-08 | 9.03E-02 |
| Eif5b | 9.57E-10 | 4.94E-08 | 9.01E-02 |
| Cald1 | 9.65E-10 | 4.97E-08 | 9.01E-02 |
| Pebp1 | 1.01E-09 | 5.16E-08 | 9.00E-02 |
| Ppt2 | 1.01E-09 | 5.16E-08 | 9.00E-02 |
| Chmp2b | 1.02E-09 | 5.18E-08 | 9.00E-02 |
| Postn | 1.04E-09 | 5.26E-08 | 8.99E-02 |
| P4hb | 1.04E-09 | 5.28E-08 | 8.99E-02 |
| Chmp6 | 1.07E-09 | 5.34E-08 | 8.99E-02 |
| Ndufa7 | 1.07E-09 | 5.34E-08 | 8.99E-02 |
| Dctn6 | 1.09E-09 | 5.43E-08 | 8.98E-02 |
| Pdgfa | 1.09E-09 | 5.43E-08 | 8.98E-02 |
| Mocs2 | 1.10E-09 | 5.43E-08 | 8.98E-02 |
| Ecm1 | 1.10E-09 | 5.44E-08 | 8.98E-02 |
| Gde1 | 1.11E-09 | 5.45E-08 | 8.98E-02 |
| Sharpin | 1.11E-09 | 5.46E-08 | 8.98E-02 |
| Eif6 | 1.12E-09 | 5.50E-08 | 8.97E-02 |
| Bag1 | 1.13E-09 | 5.52E-08 | 8.97E-02 |
| Psmd5 | 1.16E-09 | 5.63E-08 | 8.97E-02 |
| Nutf2 | 1.17E-09 | 5.67E-08 | 8.97E-02 |
| Ccdc91 | 1.21E-09 | 5.83E-08 | 8.96E-02 |
| Mrps33 | 1.22E-09 | 5.85E-08 | 8.95E-02 |
| Lat2 | 1.26E-09 | 6.01E-08 | 8.95E-02 |
| Atg5 | 1.27E-09 | 6.04E-08 | 8.95E-02 |
| Timm17b | 1.28E-09 | 6.05E-08 | 8.94E-02 |
| Csrp1 | 1.28E-09 | 6.05E-08 | 8.94E-02 |
| Cyb5r4 | 1.29E-09 | 6.05E-08 | 8.94E-02 |
| Caprin1 | 1.30E-09 | 6.11E-08 | 8.94E-02 |
| Rassf3 | 1.34E-09 | 6.27E-08 | 8.93E-02 |
| Aebp1 | 1.34E-09 | 6.27E-08 | 8.93E-02 |
| Nip7 | 1.38E-09 | 6.44E-08 | 8.93E-02 |
| Wls | 1.45E-09 | 6.70E-08 | 8.91E-02 |
| Mrpl4 | 1.49E-09 | 6.89E-08 | 8.91E-02 |
| Csf2rb2 | 1.54E-09 | 7.05E-08 | 8.90E-02 |
| Mpc2 | 1.57E-09 | 7.18E-08 | 8.90E-02 |
| Cd22 | 1.58E-09 | 7.19E-08 | 8.89E-02 |
| Ahcyl1 | 1.58E-09 | 7.20E-08 | 8.89E-02 |
| Epas1 | 1.61E-09 | 7.30E-08 | 8.89E-02 |
| Rab18 | 1.61E-09 | 7.30E-08 | 8.89E-02 |
| AY036118 | 1.63E-09 | 7.34E-08 | 8.89E-02 |
| Polr2j | 1.64E-09 | 7.37E-08 | 8.88E-02 |

| | | | |
|---|---|---|---|
| Commd3 | 1.69E-09 | 7.49E-08 | 8.88E-02 |
| Comtd1 | 1.69E-09 | 7.49E-08 | 8.88E-02 |
| Ckap4 | 1.69E-09 | 7.49E-08 | 8.88E-02 |
| Creb3l1 | 1.75E-09 | 7.69E-08 | 8.87E-02 |
| Kdelr1 | 1.75E-09 | 7.69E-08 | 8.87E-02 |
| Klc4 | 1.78E-09 | 7.78E-08 | 8.87E-02 |
| Phlda3 | 1.82E-09 | 7.94E-08 | 8.86E-02 |
| Sypl | 1.84E-09 | 7.97E-08 | 8.86E-02 |
| Prdx4 | 1.84E-09 | 7.97E-08 | 8.86E-02 |
| Ssbp1 | 1.88E-09 | 8.13E-08 | 8.85E-02 |
| Sptssa | 1.90E-09 | 8.17E-08 | 8.85E-02 |
| Msrb1 | 1.92E-09 | 8.21E-08 | 8.85E-02 |
| Qdpr | 1.93E-09 | 8.23E-08 | 8.85E-02 |
| Cpq | 1.98E-09 | 8.42E-08 | 8.84E-02 |
| Mkl2 | 2.10E-09 | 8.88E-08 | 8.83E-02 |
| Mrps28 | 2.14E-09 | 8.98E-08 | 8.82E-02 |
| Rbp1 | 2.15E-09 | 8.99E-08 | 8.82E-02 |
| Sigmar1 | 2.16E-09 | 9.01E-08 | 8.82E-02 |
| Slc25a4 | 2.23E-09 | 9.29E-08 | 8.81E-02 |
| Copa | 2.25E-09 | 9.32E-08 | 8.81E-02 |
| Psmd7 | 2.33E-09 | 9.60E-08 | 8.80E-02 |
| Fth1 | 2.43E-09 | 9.95E-08 | 8.79E-02 |
| Ostc | 2.45E-09 | 1.00E-07 | 8.79E-02 |
| Cat | 2.52E-09 | 1.02E-07 | 8.78E-02 |
| Tsfm | 2.52E-09 | 1.02E-07 | 8.78E-02 |
| Ndufb6 | 2.55E-09 | 1.03E-07 | 8.78E-02 |
| Mpv17l2 | 2.68E-09 | 1.08E-07 | 8.77E-02 |
| Paox | 2.71E-09 | 1.09E-07 | 8.77E-02 |
| 1110008F13Rik | 2.72E-09 | 1.09E-07 | 8.76E-02 |
| Spryd3 | 2.84E-09 | 1.13E-07 | 8.75E-02 |
| Mpc1 | 2.90E-09 | 1.15E-07 | 8.75E-02 |
| Tmem30a | 2.91E-09 | 1.15E-07 | 8.75E-02 |
| Bola1 | 2.94E-09 | 1.16E-07 | 8.75E-02 |
| Timm17a | 2.98E-09 | 1.18E-07 | 8.74E-02 |
| Acp2 | 3.03E-09 | 1.19E-07 | 8.74E-02 |
| Plgrkt | 3.05E-09 | 1.20E-07 | 8.74E-02 |
| Iqsec1 | 3.10E-09 | 1.21E-07 | 8.73E-02 |
| Htra1 | 3.13E-09 | 1.22E-07 | 8.73E-02 |
| Alg3 | 3.17E-09 | 1.23E-07 | 8.73E-02 |
| Gm10269 | 3.21E-09 | 1.24E-07 | 8.72E-02 |
| Chchd10 | 3.23E-09 | 1.25E-07 | 8.72E-02 |
| Cdk4 | 3.35E-09 | 1.29E-07 | 8.71E-02 |
| Ap2s1 | 3.36E-09 | 1.29E-07 | 8.71E-02 |
| Timp3 | 3.42E-09 | 1.31E-07 | 8.71E-02 |

| | | | |
|---|---|---|---|
| Bcas3 | 3.43E-09 | 1.31E-07 | 8.71E-02 |
| Ddx1 | 3.46E-09 | 1.32E-07 | 8.71E-02 |
| Glipr1 | 3.64E-09 | 1.37E-07 | 8.69E-02 |
| Smarcd1 | 3.67E-09 | 1.38E-07 | 8.69E-02 |
| Srsf6 | 3.68E-09 | 1.38E-07 | 8.69E-02 |
| Vdac2 | 3.70E-09 | 1.38E-07 | 8.69E-02 |
| Gga2 | 3.78E-09 | 1.41E-07 | 8.68E-02 |
| Cers2 | 3.84E-09 | 1.43E-07 | 8.68E-02 |
| Cebpzos | 3.88E-09 | 1.44E-07 | 8.68E-02 |
| Bhlhe41 | 3.90E-09 | 1.44E-07 | 8.68E-02 |
| Slc48a1 | 3.93E-09 | 1.45E-07 | 8.68E-02 |
| Tex261 | 3.99E-09 | 1.47E-07 | 8.67E-02 |
| Mid1ip1 | 4.03E-09 | 1.48E-07 | 8.67E-02 |
| Tmem165 | 4.04E-09 | 1.48E-07 | 8.67E-02 |
| Ano6 | 4.06E-09 | 1.48E-07 | 8.67E-02 |
| Spsb4 | 4.06E-09 | 1.48E-07 | 8.67E-02 |
| Ttc1 | 4.07E-09 | 1.48E-07 | 8.67E-02 |
| Ttc7b | 4.09E-09 | 1.48E-07 | 8.67E-02 |
| Samd8 | 4.16E-09 | 1.50E-07 | 8.66E-02 |
| Lamtor2 | 4.17E-09 | 1.50E-07 | 8.66E-02 |
| Fabp4 | 4.22E-09 | 1.51E-07 | 8.66E-02 |
| Eif3j2 | 4.30E-09 | 1.54E-07 | 8.65E-02 |
| Acox1 | 4.33E-09 | 1.54E-07 | 8.65E-02 |
| Pdrg1 | 4.34E-09 | 1.54E-07 | 8.65E-02 |
| Lrpap1 | 4.36E-09 | 1.55E-07 | 8.65E-02 |
| Med21 | 4.45E-09 | 1.57E-07 | 8.65E-02 |
| Sfr1 | 4.48E-09 | 1.58E-07 | 8.64E-02 |
| Fdx1l | 4.51E-09 | 1.58E-07 | 8.64E-02 |
| Hsd17b4 | 4.59E-09 | 1.61E-07 | 8.64E-02 |
| Tmco1 | 4.70E-09 | 1.64E-07 | 8.63E-02 |
| Mrps24 | 5.01E-09 | 1.74E-07 | 8.62E-02 |
| Nras | 5.06E-09 | 1.75E-07 | 8.61E-02 |
| Mfhas1 | 5.09E-09 | 1.75E-07 | 8.61E-02 |
| Sfrp2 | 5.10E-09 | 1.75E-07 | 8.61E-02 |
| Gm8973 | 5.11E-09 | 1.75E-07 | 8.61E-02 |
| Lamtor5 | 5.27E-09 | 1.80E-07 | 8.60E-02 |
| Gm10094 | 5.29E-09 | 1.80E-07 | 8.60E-02 |
| Gns | 5.32E-09 | 1.81E-07 | 8.60E-02 |
| Atp2a2 | 5.36E-09 | 1.82E-07 | 8.60E-02 |
| 1110008P14Rik | 5.58E-09 | 1.88E-07 | 8.59E-02 |
| Mmp14 | 5.77E-09 | 1.93E-07 | 8.58E-02 |
| Tns3 | 5.86E-09 | 1.95E-07 | 8.58E-02 |
| Gba | 5.87E-09 | 1.95E-07 | 8.58E-02 |
| Calm1 | 5.96E-09 | 1.98E-07 | 8.57E-02 |

| | | | |
|---|---|---|---|
| Srprb | 6.03E-09 | 2.00E-07 | 8.57E-02 |
| Ctbp1 | 6.07E-09 | 2.01E-07 | 8.57E-02 |
| Rab11a | 6.10E-09 | 2.01E-07 | 8.57E-02 |
| Pqlc2 | 6.23E-09 | 2.05E-07 | 8.56E-02 |
| Prdx1 | 6.39E-09 | 2.08E-07 | 8.56E-02 |
| Galns | 6.46E-09 | 2.10E-07 | 8.55E-02 |
| Lpp | 6.46E-09 | 2.10E-07 | 8.55E-02 |
| Nfic | 6.55E-09 | 2.12E-07 | 8.55E-02 |
| Pkib | 6.61E-09 | 2.13E-07 | 8.55E-02 |
| Gorasp2 | 6.63E-09 | 2.14E-07 | 8.55E-02 |
| Pdap1 | 6.65E-09 | 2.14E-07 | 8.55E-02 |
| Ubr4 | 6.74E-09 | 2.16E-07 | 8.54E-02 |
| Usf2 | 6.76E-09 | 2.16E-07 | 8.54E-02 |
| Ndufb9 | 6.84E-09 | 2.18E-07 | 8.54E-02 |
| Mgst3 | 6.85E-09 | 2.18E-07 | 8.54E-02 |
| Syne1 | 6.88E-09 | 2.18E-07 | 8.54E-02 |
| Sf3b4 | 7.07E-09 | 2.24E-07 | 8.53E-02 |
| Smim7 | 7.14E-09 | 2.26E-07 | 8.53E-02 |
| Foxk2 | 7.22E-09 | 2.27E-07 | 8.53E-02 |
| Nop10 | 7.43E-09 | 2.33E-07 | 8.52E-02 |
| Nptn | 7.91E-09 | 2.47E-07 | 8.50E-02 |
| Hibadh | 8.03E-09 | 2.50E-07 | 8.50E-02 |
| Mrpl41 | 8.08E-09 | 2.51E-07 | 8.50E-02 |
| Phf5a | 8.19E-09 | 2.54E-07 | 8.50E-02 |
| Jtb | 8.24E-09 | 2.55E-07 | 8.49E-02 |
| Rai14 | 8.45E-09 | 2.60E-07 | 8.49E-02 |
| Mfsd12 | 8.54E-09 | 2.62E-07 | 8.49E-02 |
| Dstn | 8.77E-09 | 2.68E-07 | 8.48E-02 |
| Desi2 | 8.98E-09 | 2.73E-07 | 8.47E-02 |
| Skp1a | 9.11E-09 | 2.75E-07 | 8.47E-02 |
| Lhfpl2 | 9.11E-09 | 2.75E-07 | 8.47E-02 |
| Mif | 9.24E-09 | 2.79E-07 | 8.47E-02 |
| Eri3 | 9.28E-09 | 2.79E-07 | 8.46E-02 |
| Mrpl43 | 9.31E-09 | 2.80E-07 | 8.46E-02 |
| Wdr83 | 9.55E-09 | 2.86E-07 | 8.46E-02 |
| Mrps14 | 9.67E-09 | 2.89E-07 | 8.45E-02 |
| Ift74 | 9.80E-09 | 2.92E-07 | 8.45E-02 |
| Lsm7 | 9.85E-09 | 2.93E-07 | 8.45E-02 |
| Steap3 | 9.93E-09 | 2.95E-07 | 8.45E-02 |
| Tm2d1 | 1.01E-08 | 2.99E-07 | 8.44E-02 |
| Sae1 | 1.02E-08 | 3.02E-07 | 8.44E-02 |
| Oxsm | 1.04E-08 | 3.06E-07 | 8.44E-02 |
| Ier3ip1 | 1.04E-08 | 3.07E-07 | 8.44E-02 |
| Coq2 | 1.04E-08 | 3.07E-07 | 8.44E-02 |

| | | | |
|---|---|---|---|
| Akip1 | 1.09E-08 | 3.19E-07 | 8.42E-02 |
| Dpp7 | 1.09E-08 | 3.19E-07 | 8.42E-02 |
| Lmna | 1.09E-08 | 3.19E-07 | 8.42E-02 |
| Ndufb4 | 1.14E-08 | 3.30E-07 | 8.41E-02 |
| Nudcd2 | 1.15E-08 | 3.32E-07 | 8.41E-02 |
| Med29 | 1.16E-08 | 3.35E-07 | 8.41E-02 |
| Gm9774 | 1.17E-08 | 3.36E-07 | 8.41E-02 |
| Mrpl42 | 1.17E-08 | 3.37E-07 | 8.41E-02 |
| 1810058I24Rik | 1.18E-08 | 3.39E-07 | 8.40E-02 |
| Mrps25 | 1.20E-08 | 3.42E-07 | 8.40E-02 |
| Eci1 | 1.22E-08 | 3.47E-07 | 8.40E-02 |
| Snx12 | 1.26E-08 | 3.59E-07 | 8.39E-02 |
| Sash1 | 1.28E-08 | 3.62E-07 | 8.38E-02 |
| Trappc4 | 1.30E-08 | 3.65E-07 | 8.38E-02 |
| Deb1 | 1.30E-08 | 3.65E-07 | 8.38E-02 |
| Pcmt1 | 1.30E-08 | 3.66E-07 | 8.38E-02 |
| Mrps36 | 1.31E-08 | 3.67E-07 | 8.38E-02 |
| Ywhag | 1.32E-08 | 3.69E-07 | 8.38E-02 |
| Ubash3b | 1.33E-08 | 3.71E-07 | 8.37E-02 |
| Adam8 | 1.35E-08 | 3.75E-07 | 8.37E-02 |
| Tax1bp3 | 1.35E-08 | 3.75E-07 | 8.37E-02 |
| Ndufa1 | 1.39E-08 | 3.86E-07 | 8.36E-02 |
| Usp50 | 1.45E-08 | 3.99E-07 | 8.35E-02 |
| Dynlt3 | 1.45E-08 | 3.99E-07 | 8.35E-02 |
| Plin3 | 1.45E-08 | 3.99E-07 | 8.35E-02 |
| Btk | 1.45E-08 | 3.99E-07 | 8.35E-02 |
| Ssr1 | 1.45E-08 | 3.99E-07 | 8.35E-02 |
| Fam213a | 1.48E-08 | 4.08E-07 | 8.35E-02 |
| Gm10031 | 1.49E-08 | 4.08E-07 | 8.35E-02 |
| Bach2os | 1.49E-08 | 4.08E-07 | 8.35E-02 |
| Mrpl55 | 1.53E-08 | 4.18E-07 | 8.34E-02 |
| Dlat | 1.56E-08 | 4.25E-07 | 8.33E-02 |
| Otud6b | 1.61E-08 | 4.37E-07 | 8.33E-02 |
| Timm10 | 1.62E-08 | 4.38E-07 | 8.33E-02 |
| Plec | 1.67E-08 | 4.51E-07 | 8.32E-02 |
| Sap18 | 1.68E-08 | 4.53E-07 | 8.32E-02 |
| Col6a1 | 1.68E-08 | 4.53E-07 | 8.32E-02 |
| Rab2a | 1.73E-08 | 4.66E-07 | 8.31E-02 |
| Clip1 | 1.74E-08 | 4.66E-07 | 8.31E-02 |
| Mrps16 | 1.74E-08 | 4.67E-07 | 8.31E-02 |
| Glo1 | 1.76E-08 | 4.69E-07 | 8.30E-02 |
| Bcas2 | 1.76E-08 | 4.69E-07 | 8.30E-02 |
| Slc37a2 | 1.76E-08 | 4.69E-07 | 8.30E-02 |
| Gm5426 | 1.76E-08 | 4.69E-07 | 8.30E-02 |

| | | | |
|---|---|---|---|
| Ctnnb1 | 1.79E-08 | 4.75E-07 | 8.30E-02 |
| Itga9 | 1.84E-08 | 4.88E-07 | 8.29E-02 |
| Psmd4 | 1.85E-08 | 4.90E-07 | 8.29E-02 |
| Rab1b | 1.88E-08 | 4.96E-07 | 8.29E-02 |
| Ctsa | 1.89E-08 | 4.99E-07 | 8.29E-02 |
| Dhrs1 | 1.90E-08 | 5.00E-07 | 8.28E-02 |
| Lrp12 | 1.92E-08 | 5.02E-07 | 8.28E-02 |
| Slc35b1 | 1.92E-08 | 5.02E-07 | 8.28E-02 |
| Papola | 1.93E-08 | 5.05E-07 | 8.28E-02 |
| Mrpl20 | 1.94E-08 | 5.05E-07 | 8.28E-02 |
| Rnaseh2c | 1.96E-08 | 5.10E-07 | 8.28E-02 |
| Plpp3 | 1.98E-08 | 5.15E-07 | 8.27E-02 |
| Atp6v0b | 2.00E-08 | 5.17E-07 | 8.27E-02 |
| Nme2 | 2.00E-08 | 5.18E-07 | 8.27E-02 |
| Arl3 | 2.04E-08 | 5.27E-07 | 8.27E-02 |
| Zak | 2.09E-08 | 5.38E-07 | 8.26E-02 |
| Ndufs5 | 2.10E-08 | 5.40E-07 | 8.26E-02 |
| Ube2v1 | 2.12E-08 | 5.43E-07 | 8.26E-02 |
| Vkorc1 | 2.13E-08 | 5.45E-07 | 8.26E-02 |
| Tspan3 | 2.13E-08 | 5.45E-07 | 8.26E-02 |
| S100a1 | 2.14E-08 | 5.47E-07 | 8.25E-02 |
| Tpi1 | 2.15E-08 | 5.48E-07 | 8.25E-02 |
| 2610001J05Rik | 2.19E-08 | 5.56E-07 | 8.25E-02 |
| Anxa2 | 2.21E-08 | 5.60E-07 | 8.25E-02 |
| Wbp1l | 2.24E-08 | 5.67E-07 | 8.24E-02 |
| Mtss1 | 2.24E-08 | 5.67E-07 | 8.24E-02 |
| Lrrc27 | 2.26E-08 | 5.72E-07 | 8.24E-02 |
| Wsb2 | 2.29E-08 | 5.77E-07 | 8.24E-02 |
| Kcmf1 | 2.33E-08 | 5.84E-07 | 8.23E-02 |
| Pdia6 | 2.37E-08 | 5.93E-07 | 8.23E-02 |
| Npy | 2.40E-08 | 5.99E-07 | 8.23E-02 |
| B430306N03Rik | 2.43E-08 | 6.04E-07 | 8.22E-02 |
| Cd63 | 2.46E-08 | 6.08E-07 | 8.22E-02 |
| Hdhd2 | 2.54E-08 | 6.24E-07 | 8.21E-02 |
| Psmb4 | 2.60E-08 | 6.36E-07 | 8.21E-02 |
| Atp6v1e1 | 2.60E-08 | 6.36E-07 | 8.20E-02 |
| Acadsb | 2.65E-08 | 6.47E-07 | 8.20E-02 |
| Tom1 | 2.76E-08 | 6.71E-07 | 8.19E-02 |
| Cript | 2.77E-08 | 6.73E-07 | 8.19E-02 |
| Rdx | 2.78E-08 | 6.74E-07 | 8.19E-02 |
| Bckdk | 2.79E-08 | 6.75E-07 | 8.19E-02 |
| Tmem242 | 2.80E-08 | 6.77E-07 | 8.19E-02 |
| Vps4b | 2.82E-08 | 6.81E-07 | 8.18E-02 |
| Psmg1 | 2.83E-08 | 6.82E-07 | 8.18E-02 |

| | | | |
|---|---|---|---|
| Rfk | 2.84E-08 | 6.83E-07 | 8.18E-02 |
| Gm12166 | 2.85E-08 | 6.83E-07 | 8.18E-02 |
| Ap1s1 | 2.89E-08 | 6.90E-07 | 8.18E-02 |
| Thap7 | 2.90E-08 | 6.91E-07 | 8.18E-02 |
| Dnajb14 | 2.94E-08 | 7.01E-07 | 8.17E-02 |
| Tns1 | 2.97E-08 | 7.06E-07 | 8.17E-02 |
| Faim | 2.97E-08 | 7.06E-07 | 8.17E-02 |
| Tlk1 | 3.04E-08 | 7.21E-07 | 8.16E-02 |
| Rab6a | 3.09E-08 | 7.30E-07 | 8.16E-02 |
| Ank | 3.10E-08 | 7.31E-07 | 8.16E-02 |
| 0610010K14Rik | 3.24E-08 | 7.61E-07 | 8.15E-02 |
| Ndufs1 | 3.30E-08 | 7.73E-07 | 8.14E-02 |
| Tubb4b | 3.33E-08 | 7.80E-07 | 8.14E-02 |
| Sdhc | 3.43E-08 | 7.98E-07 | 8.13E-02 |
| Anpep | 3.46E-08 | 8.03E-07 | 8.13E-02 |
| Pdcd6 | 3.46E-08 | 8.03E-07 | 8.13E-02 |
| Ywhaq | 3.46E-08 | 8.03E-07 | 8.13E-02 |
| Glrx2 | 3.48E-08 | 8.04E-07 | 8.13E-02 |
| Carkd | 3.48E-08 | 8.04E-07 | 8.13E-02 |
| Gm7808 | 3.51E-08 | 8.07E-07 | 8.13E-02 |
| Tomm6 | 3.58E-08 | 8.22E-07 | 8.12E-02 |
| Bbip1 | 3.62E-08 | 8.27E-07 | 8.12E-02 |
| Akirin1 | 3.63E-08 | 8.27E-07 | 8.12E-02 |
| Fnip2 | 3.63E-08 | 8.27E-07 | 8.12E-02 |
| Apoo | 3.65E-08 | 8.30E-07 | 8.12E-02 |
| Tpgs1 | 3.70E-08 | 8.40E-07 | 8.11E-02 |
| Secisbp2l | 3.73E-08 | 8.44E-07 | 8.11E-02 |
| Dhx32 | 3.79E-08 | 8.56E-07 | 8.11E-02 |
| Wdr77 | 3.82E-08 | 8.60E-07 | 8.11E-02 |
| Gabarapl1 | 3.83E-08 | 8.60E-07 | 8.11E-02 |
| 2210016F16Rik | 3.84E-08 | 8.62E-07 | 8.10E-02 |
| Reep5 | 3.86E-08 | 8.66E-07 | 8.10E-02 |
| Zfp91 | 3.88E-08 | 8.69E-07 | 8.10E-02 |
| Mmadhc | 3.88E-08 | 8.69E-07 | 8.10E-02 |
| Serpine2 | 3.90E-08 | 8.72E-07 | 8.10E-02 |
| Lyz1 | 3.91E-08 | 8.72E-07 | 8.10E-02 |
| 1500015O10Rik | 3.94E-08 | 8.78E-07 | 8.10E-02 |
| Idh3a | 4.00E-08 | 8.91E-07 | 8.09E-02 |
| Tmem106b | 4.04E-08 | 8.97E-07 | 8.09E-02 |
| Txnl4a | 4.05E-08 | 8.97E-07 | 8.09E-02 |
| Sumf1 | 4.07E-08 | 8.98E-07 | 8.09E-02 |
| Atp1b3 | 4.07E-08 | 8.98E-07 | 8.09E-02 |
| Dtd2 | 4.08E-08 | 8.99E-07 | 8.09E-02 |
| Yeats4 | 4.09E-08 | 8.99E-07 | 8.09E-02 |

| | | | |
|---|---|---|---|
| Hdgf | 4.14E-08 | 9.09E-07 | 8.08E-02 |
| Timm22 | 4.18E-08 | 9.17E-07 | 8.08E-02 |
| Magt1 | 4.25E-08 | 9.31E-07 | 8.08E-02 |
| Gstm5 | 4.27E-08 | 9.35E-07 | 8.08E-02 |
| Nek6 | 4.34E-08 | 9.48E-07 | 8.07E-02 |
| Oaz2 | 4.35E-08 | 9.49E-07 | 8.07E-02 |
| Crtap | 4.37E-08 | 9.52E-07 | 8.07E-02 |
| Snrnp27 | 4.39E-08 | 9.55E-07 | 8.07E-02 |
| Cib1 | 4.43E-08 | 9.62E-07 | 8.07E-02 |
| Cdv3 | 4.46E-08 | 9.67E-07 | 8.07E-02 |
| 1110004F10Rik | 4.46E-08 | 9.67E-07 | 8.07E-02 |
| Sdccag3 | 4.52E-08 | 9.76E-07 | 8.06E-02 |
| Pafah1b1 | 4.61E-08 | 9.93E-07 | 8.06E-02 |
| Ppa1 | 4.64E-08 | 9.97E-07 | 8.06E-02 |
| Angpt2 | 4.66E-08 | 1.00E-06 | 8.05E-02 |
| Cyb5r1 | 4.67E-08 | 1.00E-06 | 8.05E-02 |
| Zranb2 | 4.67E-08 | 1.00E-06 | 8.05E-02 |
| Mmp27 | 4.77E-08 | 1.02E-06 | 8.05E-02 |
| Sar1a | 4.86E-08 | 1.03E-06 | 8.04E-02 |
| Bet1 | 4.87E-08 | 1.03E-06 | 8.04E-02 |
| Pkd1 | 4.97E-08 | 1.05E-06 | 8.04E-02 |
| Ap3m1 | 5.02E-08 | 1.06E-06 | 8.03E-02 |
| Pgrmc1 | 5.05E-08 | 1.06E-06 | 8.03E-02 |
| Eea1 | 5.05E-08 | 1.06E-06 | 8.03E-02 |
| Gm4950 | 5.11E-08 | 1.07E-06 | 8.03E-02 |
| Ormdl1 | 5.12E-08 | 1.07E-06 | 8.03E-02 |
| Myof | 5.13E-08 | 1.07E-06 | 8.03E-02 |
| Snx3 | 5.17E-08 | 1.08E-06 | 8.03E-02 |
| Col5a2 | 5.29E-08 | 1.10E-06 | 8.02E-02 |
| Tmx4 | 5.41E-08 | 1.13E-06 | 8.01E-02 |
| Tbc1d7 | 5.48E-08 | 1.14E-06 | 8.01E-02 |
| Snrpd3 | 5.50E-08 | 1.14E-06 | 8.01E-02 |
| B3gat3 | 5.66E-08 | 1.17E-06 | 8.00E-02 |
| Coq7 | 5.68E-08 | 1.18E-06 | 8.00E-02 |
| Tmem120a | 5.81E-08 | 1.20E-06 | 8.00E-02 |
| Cnih1 | 5.82E-08 | 1.20E-06 | 8.00E-02 |
| 0610009B22Rik | 5.83E-08 | 1.20E-06 | 8.00E-02 |
| Frrs1 | 5.91E-08 | 1.22E-06 | 7.99E-02 |
| Taldo1 | 5.95E-08 | 1.22E-06 | 7.99E-02 |
| Tceal8 | 5.96E-08 | 1.22E-06 | 7.99E-02 |
| Lrrfip2 | 5.97E-08 | 1.22E-06 | 7.99E-02 |
| Atp6ap1 | 5.98E-08 | 1.22E-06 | 7.99E-02 |
| Emc8 | 6.00E-08 | 1.23E-06 | 7.99E-02 |
| Gm5900 | 6.10E-08 | 1.25E-06 | 7.98E-02 |

| | | | |
|---|---|---|---|
| Cluh | 6.21E-08 | 1.27E-06 | 7.98E-02 |
| Tmf1 | 6.33E-08 | 1.29E-06 | 7.97E-02 |
| Bnip3l | 6.34E-08 | 1.29E-06 | 7.97E-02 |
| Atp5g2 | 6.41E-08 | 1.30E-06 | 7.97E-02 |
| Triap1 | 6.48E-08 | 1.31E-06 | 7.97E-02 |
| Glyr1 | 6.55E-08 | 1.32E-06 | 7.96E-02 |
| Zmynd8 | 6.56E-08 | 1.32E-06 | 7.96E-02 |
| Mrpl49 | 6.62E-08 | 1.34E-06 | 7.96E-02 |
| Zcrb1 | 6.63E-08 | 1.34E-06 | 7.96E-02 |
| Prelp | 6.67E-08 | 1.34E-06 | 7.96E-02 |
| Tomm20 | 6.79E-08 | 1.36E-06 | 7.95E-02 |
| Eif1a | 6.83E-08 | 1.37E-06 | 7.95E-02 |
| Snrpd1 | 6.84E-08 | 1.37E-06 | 7.95E-02 |
| Emc9 | 6.84E-08 | 1.37E-06 | 7.95E-02 |
| Ercc1 | 7.00E-08 | 1.39E-06 | 7.95E-02 |
| Emc3 | 7.06E-08 | 1.41E-06 | 7.94E-02 |
| March2 | 7.09E-08 | 1.41E-06 | 7.94E-02 |
| Ldha | 7.28E-08 | 1.44E-06 | 7.94E-02 |
| Gm10320 | 7.32E-08 | 1.44E-06 | 7.94E-02 |
| Pqlc3 | 7.46E-08 | 1.47E-06 | 7.93E-02 |
| Hcfc1r1 | 7.51E-08 | 1.47E-06 | 7.93E-02 |
| Abtb1 | 7.52E-08 | 1.47E-06 | 7.93E-02 |
| Hexb | 7.59E-08 | 1.49E-06 | 7.93E-02 |
| Nme1 | 7.65E-08 | 1.50E-06 | 7.92E-02 |
| Naglu | 7.73E-08 | 1.51E-06 | 7.92E-02 |
| Cops3 | 7.86E-08 | 1.53E-06 | 7.92E-02 |
| Alg1 | 7.90E-08 | 1.54E-06 | 7.92E-02 |
| Spsb2 | 8.00E-08 | 1.55E-06 | 7.91E-02 |
| Brd7 | 8.05E-08 | 1.56E-06 | 7.91E-02 |
| Gm16286 | 8.07E-08 | 1.56E-06 | 7.91E-02 |
| Commd5 | 8.10E-08 | 1.56E-06 | 7.91E-02 |
| Abi3 | 8.31E-08 | 1.59E-06 | 7.90E-02 |
| Rfesd | 8.39E-08 | 1.61E-06 | 7.90E-02 |
| Polr2m | 8.44E-08 | 1.61E-06 | 7.90E-02 |
| Slc25a3 | 8.49E-08 | 1.62E-06 | 7.90E-02 |
| Abcd3 | 8.50E-08 | 1.62E-06 | 7.90E-02 |
| Ptgr1 | 8.62E-08 | 1.64E-06 | 7.89E-02 |
| Borcs7 | 8.62E-08 | 1.64E-06 | 7.89E-02 |
| Hmgn1 | 8.67E-08 | 1.64E-06 | 7.89E-02 |
| Polr2i | 8.68E-08 | 1.65E-06 | 7.89E-02 |
| Cdc34 | 8.76E-08 | 1.65E-06 | 7.89E-02 |
| C1d | 8.77E-08 | 1.65E-06 | 7.89E-02 |
| Cdk18 | 9.00E-08 | 1.69E-06 | 7.88E-02 |
| Tmem237 | 9.25E-08 | 1.73E-06 | 7.87E-02 |

| | | | |
|---|---|---|---|
| Dapk3 | 9.29E-08 | 1.74E-06 | 7.87E-02 |
| Hpcal1 | 9.35E-08 | 1.74E-06 | 7.87E-02 |
| 5031439G07Rik | 9.36E-08 | 1.75E-06 | 7.87E-02 |
| Armcx3 | 9.39E-08 | 1.75E-06 | 7.87E-02 |
| Ormdl2 | 9.44E-08 | 1.75E-06 | 7.87E-02 |
| Sgcb | 9.50E-08 | 1.76E-06 | 7.87E-02 |
| Mtch1 | 9.53E-08 | 1.77E-06 | 7.87E-02 |
| Hnrnpa1 | 9.54E-08 | 1.77E-06 | 7.86E-02 |
| Mbd3 | 9.58E-08 | 1.77E-06 | 7.86E-02 |
| Aig1 | 9.68E-08 | 1.79E-06 | 7.86E-02 |
| Tecr | 9.81E-08 | 1.81E-06 | 7.86E-02 |
| Lsm8 | 9.91E-08 | 1.82E-06 | 7.85E-02 |
| Jagn1 | 9.97E-08 | 1.83E-06 | 7.85E-02 |
| Col6a3 | 1.01E-07 | 1.85E-06 | 7.85E-02 |
| 4930455G09Rik | 1.01E-07 | 1.85E-06 | 7.85E-02 |
| Calm3 | 1.01E-07 | 1.86E-06 | 7.85E-02 |
| 9530068E07Rik | 1.02E-07 | 1.86E-06 | 7.85E-02 |
| Clptm1l | 1.03E-07 | 1.88E-06 | 7.84E-02 |
| Suclg1 | 1.03E-07 | 1.88E-06 | 7.84E-02 |
| Derl2 | 1.03E-07 | 1.88E-06 | 7.84E-02 |
| Sgk1 | 1.04E-07 | 1.89E-06 | 7.84E-02 |
| Ctcf | 1.05E-07 | 1.90E-06 | 7.84E-02 |
| Txndc5 | 1.06E-07 | 1.92E-06 | 7.84E-02 |
| Aes | 1.07E-07 | 1.94E-06 | 7.83E-02 |
| Itpa | 1.08E-07 | 1.94E-06 | 7.83E-02 |
| Mien1 | 1.09E-07 | 1.96E-06 | 7.83E-02 |
| Acp1 | 1.09E-07 | 1.96E-06 | 7.83E-02 |
| Slc39a11 | 1.11E-07 | 1.98E-06 | 7.83E-02 |
| Tmem9b | 1.11E-07 | 1.98E-06 | 7.83E-02 |
| Ppp2ca | 1.12E-07 | 2.00E-06 | 7.82E-02 |
| Ten1 | 1.15E-07 | 2.05E-06 | 7.81E-02 |
| Serinc1 | 1.16E-07 | 2.06E-06 | 7.81E-02 |
| Erlec1 | 1.16E-07 | 2.06E-06 | 7.81E-02 |
| Nr2c2ap | 1.16E-07 | 2.06E-06 | 7.81E-02 |
| Ubl4a | 1.16E-07 | 2.06E-06 | 7.81E-02 |
| Agap1 | 1.17E-07 | 2.07E-06 | 7.81E-02 |
| Insig2 | 1.17E-07 | 2.08E-06 | 7.81E-02 |
| Gemin7 | 1.18E-07 | 2.09E-06 | 7.81E-02 |
| Trappc1 | 1.19E-07 | 2.09E-06 | 7.81E-02 |
| Taf12 | 1.22E-07 | 2.14E-06 | 7.80E-02 |
| Tardbp | 1.22E-07 | 2.15E-06 | 7.80E-02 |
| Uchl3 | 1.24E-07 | 2.17E-06 | 7.80E-02 |
| Kcnq1ot1 | 1.24E-07 | 2.17E-06 | 7.79E-02 |
| Pigyl | 1.24E-07 | 2.17E-06 | 7.79E-02 |

| | | | |
|---|---|---|---|
| Zfp260 | 1.25E-07 | 2.19E-06 | 7.79E-02 |
| B230118H07Rik | 1.26E-07 | 2.20E-06 | 7.79E-02 |
| Ndufb2 | 1.26E-07 | 2.20E-06 | 7.79E-02 |
| Zcchc9 | 1.27E-07 | 2.20E-06 | 7.79E-02 |
| Mtpn | 1.27E-07 | 2.21E-06 | 7.79E-02 |
| Map4k3 | 1.28E-07 | 2.21E-06 | 7.79E-02 |
| Scd1 | 1.28E-07 | 2.21E-06 | 7.79E-02 |
| Spr | 1.28E-07 | 2.21E-06 | 7.79E-02 |
| Ap2m1 | 1.28E-07 | 2.21E-06 | 7.79E-02 |
| Znhit1 | 1.30E-07 | 2.24E-06 | 7.78E-02 |
| Polr2k | 1.31E-07 | 2.24E-06 | 7.78E-02 |
| Wfs1 | 1.33E-07 | 2.27E-06 | 7.78E-02 |
| Coa4 | 1.33E-07 | 2.27E-06 | 7.78E-02 |
| Nudt9 | 1.36E-07 | 2.32E-06 | 7.77E-02 |
| Glud1 | 1.37E-07 | 2.33E-06 | 7.77E-02 |
| Trappc3 | 1.37E-07 | 2.33E-06 | 7.77E-02 |
| Pja2 | 1.39E-07 | 2.36E-06 | 7.76E-02 |
| Arhgap19 | 1.39E-07 | 2.37E-06 | 7.76E-02 |
| Gpx4 | 1.39E-07 | 2.37E-06 | 7.76E-02 |
| Rnf11 | 1.40E-07 | 2.37E-06 | 7.76E-02 |
| Rgs7 | 1.40E-07 | 2.37E-06 | 7.76E-02 |
| Ndufa8 | 1.41E-07 | 2.38E-06 | 7.76E-02 |
| Ctgf | 1.41E-07 | 2.38E-06 | 7.76E-02 |
| Mnat1 | 1.42E-07 | 2.39E-06 | 7.76E-02 |
| Arl1 | 1.43E-07 | 2.40E-06 | 7.76E-02 |
| Psmd10 | 1.45E-07 | 2.45E-06 | 7.75E-02 |
| Asna1 | 1.47E-07 | 2.47E-06 | 7.75E-02 |
| Timp1 | 1.48E-07 | 2.49E-06 | 7.75E-02 |
| Bcap31 | 1.50E-07 | 2.51E-06 | 7.74E-02 |
| Mettl9 | 1.50E-07 | 2.51E-06 | 7.74E-02 |
| Paip2 | 1.50E-07 | 2.51E-06 | 7.74E-02 |
| Hadh | 1.52E-07 | 2.54E-06 | 7.74E-02 |
| Anapc16 | 1.53E-07 | 2.54E-06 | 7.74E-02 |
| Isca2 | 1.53E-07 | 2.54E-06 | 7.74E-02 |
| Grpel1 | 1.53E-07 | 2.54E-06 | 7.74E-02 |
| Timp2 | 1.53E-07 | 2.54E-06 | 7.74E-02 |
| Smpd1 | 1.54E-07 | 2.55E-06 | 7.74E-02 |
| Klc1 | 1.54E-07 | 2.55E-06 | 7.74E-02 |
| Actn4 | 1.56E-07 | 2.58E-06 | 7.73E-02 |
| Pafah1b2 | 1.57E-07 | 2.58E-06 | 7.73E-02 |
| Pcbp1 | 1.61E-07 | 2.64E-06 | 7.72E-02 |
| Dmxl2 | 1.61E-07 | 2.65E-06 | 7.72E-02 |
| Phb | 1.62E-07 | 2.66E-06 | 7.72E-02 |
| Gabarapl2 | 1.62E-07 | 2.66E-06 | 7.72E-02 |

| | | | |
|---|---|---|---|
| Lrrfip1 | 1.63E-07 | 2.67E-06 | 7.72E-02 |
| Itgax | 1.63E-07 | 2.68E-06 | 7.72E-02 |
| Daglb | 1.65E-07 | 2.69E-06 | 7.72E-02 |
| Pcbd2 | 1.70E-07 | 2.77E-06 | 7.71E-02 |
| Lpxn | 1.72E-07 | 2.79E-06 | 7.71E-02 |
| Man1c1 | 1.75E-07 | 2.82E-06 | 7.70E-02 |
| Fis1 | 1.75E-07 | 2.83E-06 | 7.70E-02 |
| Pithd1 | 1.76E-07 | 2.84E-06 | 7.70E-02 |
| Abi1 | 1.76E-07 | 2.84E-06 | 7.70E-02 |
| Wbp11 | 1.78E-07 | 2.87E-06 | 7.70E-02 |
| Ado | 1.80E-07 | 2.90E-06 | 7.69E-02 |
| Gm11273 | 1.81E-07 | 2.90E-06 | 7.69E-02 |
| Rhoc | 1.81E-07 | 2.91E-06 | 7.69E-02 |
| Hint2 | 1.85E-07 | 2.96E-06 | 7.69E-02 |
| 2310033P09Rik | 1.86E-07 | 2.97E-06 | 7.69E-02 |
| Aqp1 | 1.87E-07 | 2.98E-06 | 7.68E-02 |
| Mrpl12 | 1.87E-07 | 2.99E-06 | 7.68E-02 |
| Eif3i | 1.90E-07 | 3.02E-06 | 7.68E-02 |
| Scamp3 | 1.92E-07 | 3.04E-06 | 7.68E-02 |
| Inf2 | 1.92E-07 | 3.05E-06 | 7.68E-02 |
| Hmg20b | 1.93E-07 | 3.05E-06 | 7.68E-02 |
| Dohh | 1.94E-07 | 3.07E-06 | 7.67E-02 |
| Sec11c | 1.94E-07 | 3.07E-06 | 7.67E-02 |
| Mgat1 | 1.95E-07 | 3.09E-06 | 7.67E-02 |
| Smg1 | 1.96E-07 | 3.09E-06 | 7.67E-02 |
| Rnf187 | 1.96E-07 | 3.09E-06 | 7.67E-02 |
| Atp6v1g1 | 1.99E-07 | 3.13E-06 | 7.67E-02 |
| Tprgl | 2.00E-07 | 3.15E-06 | 7.66E-02 |
| Tarbp2 | 2.06E-07 | 3.22E-06 | 7.66E-02 |
| Rwdd4a | 2.06E-07 | 3.22E-06 | 7.66E-02 |
| Hmbs | 2.06E-07 | 3.23E-06 | 7.66E-02 |
| Grcc10 | 2.07E-07 | 3.23E-06 | 7.66E-02 |
| 2310011J03Rik | 2.07E-07 | 3.24E-06 | 7.66E-02 |
| Cct5 | 2.09E-07 | 3.26E-06 | 7.65E-02 |
| Fnta | 2.10E-07 | 3.27E-06 | 7.65E-02 |
| Atp5c1 | 2.11E-07 | 3.28E-06 | 7.65E-02 |
| Kpna2 | 2.12E-07 | 3.29E-06 | 7.65E-02 |
| Cd300lb | 2.12E-07 | 3.29E-06 | 7.65E-02 |
| Rexo2 | 2.13E-07 | 3.30E-06 | 7.65E-02 |
| Tmem38b | 2.17E-07 | 3.36E-06 | 7.64E-02 |
| Mesdc1 | 2.22E-07 | 3.42E-06 | 7.64E-02 |
| Acp5 | 2.22E-07 | 3.42E-06 | 7.64E-02 |
| Psme4 | 2.23E-07 | 3.43E-06 | 7.64E-02 |
| Irak1 | 2.23E-07 | 3.43E-06 | 7.63E-02 |

| | | | |
|---|---|---|---|
| Sdhd | 2.25E-07 | 3.45E-06 | 7.63E-02 |
| Tmem43 | 2.25E-07 | 3.46E-06 | 7.63E-02 |
| Ndufa5 | 2.26E-07 | 3.46E-06 | 7.63E-02 |
| Ufc1 | 2.31E-07 | 3.53E-06 | 7.63E-02 |
| Npc1 | 2.31E-07 | 3.53E-06 | 7.63E-02 |
| Gadd45gip1 | 2.32E-07 | 3.54E-06 | 7.62E-02 |
| Galnt6 | 2.33E-07 | 3.55E-06 | 7.62E-02 |
| Sumo3 | 2.34E-07 | 3.55E-06 | 7.62E-02 |
| Dctn4 | 2.36E-07 | 3.59E-06 | 7.62E-02 |
| Mrpl13 | 2.37E-07 | 3.60E-06 | 7.62E-02 |
| Dph3 | 2.41E-07 | 3.65E-06 | 7.61E-02 |
| Psen2 | 2.42E-07 | 3.66E-06 | 7.61E-02 |
| Agpat5 | 2.44E-07 | 3.69E-06 | 7.61E-02 |
| Armc1 | 2.44E-07 | 3.69E-06 | 7.61E-02 |
| Aspscr1 | 2.45E-07 | 3.69E-06 | 7.61E-02 |
| Csnk2b | 2.45E-07 | 3.70E-06 | 7.61E-02 |
| Serpine1 | 2.47E-07 | 3.71E-06 | 7.61E-02 |
| Ndufab1 | 2.47E-07 | 3.71E-06 | 7.61E-02 |
| Psmd9 | 2.50E-07 | 3.75E-06 | 7.60E-02 |
| Cyb561d2 | 2.56E-07 | 3.83E-06 | 7.60E-02 |
| Ergic1 | 2.57E-07 | 3.83E-06 | 7.60E-02 |
| Pmepa1 | 2.59E-07 | 3.86E-06 | 7.59E-02 |
| Ece2 | 2.62E-07 | 3.90E-06 | 7.59E-02 |
| Txnrd1 | 2.63E-07 | 3.90E-06 | 7.59E-02 |
| Flna | 2.64E-07 | 3.91E-06 | 7.59E-02 |
| Cav2 | 2.64E-07 | 3.91E-06 | 7.59E-02 |
| Hist1h2bc | 2.64E-07 | 3.92E-06 | 7.59E-02 |
| Myh9 | 2.66E-07 | 3.94E-06 | 7.59E-02 |
| Pofut2 | 2.67E-07 | 3.95E-06 | 7.59E-02 |
| Cd93 | 2.68E-07 | 3.96E-06 | 7.58E-02 |
| Ost4 | 2.69E-07 | 3.97E-06 | 7.58E-02 |
| Htatsf1 | 2.69E-07 | 3.97E-06 | 7.58E-02 |
| Atp6v0a1 | 2.71E-07 | 4.00E-06 | 7.58E-02 |
| Lsm4 | 2.76E-07 | 4.04E-06 | 7.58E-02 |
| Fam213b | 2.76E-07 | 4.04E-06 | 7.58E-02 |
| Zfp706 | 2.76E-07 | 4.04E-06 | 7.58E-02 |
| Sec13 | 2.77E-07 | 4.04E-06 | 7.58E-02 |
| Pum2 | 2.77E-07 | 4.05E-06 | 7.58E-02 |
| Mtg1 | 2.78E-07 | 4.05E-06 | 7.58E-02 |
| Itpripl2 | 2.78E-07 | 4.05E-06 | 7.57E-02 |
| Mustn1 | 2.79E-07 | 4.06E-06 | 7.57E-02 |
| Rell1 | 2.79E-07 | 4.06E-06 | 7.57E-02 |
| Tceanc2 | 2.80E-07 | 4.07E-06 | 7.57E-02 |
| Sec63 | 2.80E-07 | 4.07E-06 | 7.57E-02 |

| | | | |
|---|---|---|---|
| Mocos | 2.81E-07 | 4.07E-06 | 7.57E-02 |
| Slc25a39 | 2.87E-07 | 4.15E-06 | 7.57E-02 |
| Gm10073 | 2.90E-07 | 4.19E-06 | 7.56E-02 |
| Lgals1 | 2.91E-07 | 4.20E-06 | 7.56E-02 |
| Ddx18 | 2.91E-07 | 4.20E-06 | 7.56E-02 |
| Ostm1 | 2.91E-07 | 4.20E-06 | 7.56E-02 |
| Trp53inp2 | 2.93E-07 | 4.22E-06 | 7.56E-02 |
| Cd276 | 2.94E-07 | 4.23E-06 | 7.56E-02 |
| Hspb1 | 2.95E-07 | 4.23E-06 | 7.56E-02 |
| Rpp25l | 2.95E-07 | 4.23E-06 | 7.56E-02 |
| Tsn | 2.99E-07 | 4.28E-06 | 7.55E-02 |
| Mtfr1 | 2.99E-07 | 4.29E-06 | 7.55E-02 |
| Asph | 3.06E-07 | 4.37E-06 | 7.55E-02 |
| Ssr3 | 3.06E-07 | 4.37E-06 | 7.55E-02 |
| Tnfrsf1b | 3.12E-07 | 4.44E-06 | 7.54E-02 |
| Bloc1s1 | 3.13E-07 | 4.46E-06 | 7.54E-02 |
| Pigp | 3.14E-07 | 4.46E-06 | 7.54E-02 |
| Fam214b | 3.17E-07 | 4.50E-06 | 7.54E-02 |
| Ppp1r14b | 3.24E-07 | 4.58E-06 | 7.53E-02 |
| Myo9b | 3.33E-07 | 4.70E-06 | 7.52E-02 |
| Orc2 | 3.33E-07 | 4.70E-06 | 7.52E-02 |
| Tmed4 | 3.39E-07 | 4.76E-06 | 7.52E-02 |
| Chchd7 | 3.40E-07 | 4.77E-06 | 7.52E-02 |
| Nucks1 | 3.44E-07 | 4.82E-06 | 7.52E-02 |
| Upf1 | 3.44E-07 | 4.82E-06 | 7.52E-02 |
| Nabp2 | 3.45E-07 | 4.83E-06 | 7.51E-02 |
| Rab11b | 3.46E-07 | 4.84E-06 | 7.51E-02 |
| Fkbp4 | 3.49E-07 | 4.88E-06 | 7.51E-02 |
| St13 | 3.51E-07 | 4.90E-06 | 7.51E-02 |
| Cnpy2 | 3.52E-07 | 4.91E-06 | 7.51E-02 |
| Snx24 | 3.54E-07 | 4.93E-06 | 7.51E-02 |
| Sars | 3.55E-07 | 4.94E-06 | 7.51E-02 |
| Gid8 | 3.67E-07 | 5.09E-06 | 7.50E-02 |
| Crls1 | 3.68E-07 | 5.09E-06 | 7.50E-02 |
| Pls3 | 3.69E-07 | 5.10E-06 | 7.50E-02 |
| Pqlc1 | 3.74E-07 | 5.15E-06 | 7.49E-02 |
| Slmap | 3.74E-07 | 5.15E-06 | 7.49E-02 |
| Cxcl12 | 3.75E-07 | 5.17E-06 | 7.49E-02 |
| Emd | 3.79E-07 | 5.21E-06 | 7.49E-02 |
| Wdr74 | 3.79E-07 | 5.21E-06 | 7.49E-02 |
| Il18bp | 3.85E-07 | 5.29E-06 | 7.48E-02 |
| Tsen34 | 3.86E-07 | 5.29E-06 | 7.48E-02 |
| Iscu | 3.88E-07 | 5.32E-06 | 7.48E-02 |
| Ddx56 | 3.89E-07 | 5.32E-06 | 7.48E-02 |

| | | | |
|---|---|---|---|
| Snapin | 3.91E-07 | 5.34E-06 | 7.48E-02 |
| Mea1 | 3.92E-07 | 5.36E-06 | 7.48E-02 |
| Nudt4 | 4.00E-07 | 5.46E-06 | 7.47E-02 |
| Dnajc10 | 4.02E-07 | 5.48E-06 | 7.47E-02 |
| Cnbp | 4.04E-07 | 5.50E-06 | 7.47E-02 |
| Rnf149 | 4.06E-07 | 5.51E-06 | 7.47E-02 |
| Stra6l | 4.13E-07 | 5.60E-06 | 7.46E-02 |
| Ccdc124 | 4.15E-07 | 5.63E-06 | 7.46E-02 |
| Thyn1 | 4.16E-07 | 5.64E-06 | 7.46E-02 |
| Clec4d | 4.19E-07 | 5.67E-06 | 7.46E-02 |
| Rap2a | 4.23E-07 | 5.72E-06 | 7.46E-02 |
| Wbp4 | 4.24E-07 | 5.72E-06 | 7.46E-02 |
| Scarb2 | 4.25E-07 | 5.72E-06 | 7.46E-02 |
| Snip1 | 4.29E-07 | 5.77E-06 | 7.45E-02 |
| Gm13305 | 4.30E-07 | 5.78E-06 | 7.45E-02 |
| Fastkd3 | 4.35E-07 | 5.83E-06 | 7.45E-02 |
| Tceb1 | 4.36E-07 | 5.84E-06 | 7.45E-02 |
| Josd2 | 4.37E-07 | 5.84E-06 | 7.45E-02 |
| Col18a1 | 4.37E-07 | 5.84E-06 | 7.45E-02 |
| Yipf5 | 4.38E-07 | 5.84E-06 | 7.45E-02 |
| Oxa1l | 4.40E-07 | 5.87E-06 | 7.45E-02 |
| Ube3a | 4.41E-07 | 5.87E-06 | 7.45E-02 |
| Pex2 | 4.42E-07 | 5.88E-06 | 7.45E-02 |
| Fkbp1a | 4.43E-07 | 5.89E-06 | 7.44E-02 |
| Slirp | 4.45E-07 | 5.90E-06 | 7.44E-02 |
| Calr | 4.47E-07 | 5.93E-06 | 7.44E-02 |
| Gm561 | 4.56E-07 | 6.04E-06 | 7.44E-02 |
| Anxa3 | 4.57E-07 | 6.04E-06 | 7.44E-02 |
| Rps29 | 4.59E-07 | 6.06E-06 | -7.43E-02 |
| Mphosph6 | 4.59E-07 | 6.06E-06 | 7.43E-02 |
| Col6a2 | 4.65E-07 | 6.13E-06 | 7.43E-02 |
| Pkp4 | 4.66E-07 | 6.13E-06 | 7.43E-02 |
| Slc25a1 | 4.69E-07 | 6.17E-06 | 7.43E-02 |
| Tmem50b | 4.70E-07 | 6.18E-06 | 7.43E-02 |
| Vcam1 | 4.72E-07 | 6.21E-06 | 7.43E-02 |
| Sncg | 4.74E-07 | 6.22E-06 | 7.43E-02 |
| Adprhl2 | 4.76E-07 | 6.23E-06 | 7.42E-02 |
| Top1 | 4.78E-07 | 6.26E-06 | 7.42E-02 |
| Denr | 4.81E-07 | 6.29E-06 | 7.42E-02 |
| Slc25a5 | 4.81E-07 | 6.29E-06 | 7.42E-02 |
| Actr10 | 4.83E-07 | 6.29E-06 | 7.42E-02 |
| Wrb | 4.83E-07 | 6.29E-06 | 7.42E-02 |
| Cpeb4 | 4.84E-07 | 6.30E-06 | 7.42E-02 |
| Vegfb | 4.86E-07 | 6.32E-06 | 7.42E-02 |

| | | | |
|---|---|---|---|
| Hrsp12 | 4.91E-07 | 6.38E-06 | 7.42E-02 |
| Rragc | 5.01E-07 | 6.50E-06 | 7.41E-02 |
| Ptms | 5.03E-07 | 6.50E-06 | 7.41E-02 |
| Gsk3a | 5.03E-07 | 6.50E-06 | 7.41E-02 |
| Gm10260 | 5.08E-07 | 6.56E-06 | 7.41E-02 |
| Eepd1 | 5.11E-07 | 6.59E-06 | 7.40E-02 |
| Pop5 | 5.14E-07 | 6.61E-06 | 7.40E-02 |
| Caml | 5.14E-07 | 6.62E-06 | 7.40E-02 |
| Med8 | 5.16E-07 | 6.63E-06 | 7.40E-02 |
| Ddb1 | 5.19E-07 | 6.66E-06 | 7.40E-02 |
| Zbtb7a | 5.20E-07 | 6.67E-06 | 7.40E-02 |
| Trim35 | 5.22E-07 | 6.69E-06 | 7.40E-02 |
| Max | 5.23E-07 | 6.70E-06 | 7.40E-02 |
| Vimp | 5.26E-07 | 6.73E-06 | 7.40E-02 |
| Trim47 | 5.31E-07 | 6.78E-06 | 7.39E-02 |
| Hey1 | 5.32E-07 | 6.79E-06 | 7.39E-02 |
| Sdc1 | 5.36E-07 | 6.84E-06 | 7.39E-02 |
| Tcf12 | 5.42E-07 | 6.90E-06 | 7.39E-02 |
| Snx1 | 5.44E-07 | 6.91E-06 | 7.39E-02 |
| Shisa9 | 5.50E-07 | 6.98E-06 | 7.38E-02 |
| Syt11 | 5.54E-07 | 7.02E-06 | 7.38E-02 |
| Ptges3 | 5.59E-07 | 7.07E-06 | 7.38E-02 |
| Pgk1 | 5.64E-07 | 7.12E-06 | 7.38E-02 |
| Ppp2r1a | 5.64E-07 | 7.12E-06 | 7.38E-02 |
| Lima1 | 5.66E-07 | 7.14E-06 | 7.38E-02 |
| Kif5b | 5.67E-07 | 7.14E-06 | 7.38E-02 |
| Mob2 | 5.68E-07 | 7.15E-06 | 7.37E-02 |
| Acaa1a | 5.68E-07 | 7.15E-06 | 7.37E-02 |
| Pcnx | 5.72E-07 | 7.19E-06 | 7.37E-02 |
| Akr7a5 | 5.74E-07 | 7.21E-06 | 7.37E-02 |
| Fam103a1 | 5.77E-07 | 7.24E-06 | 7.37E-02 |
| Edem2 | 5.79E-07 | 7.26E-06 | 7.37E-02 |
| Stam2 | 5.88E-07 | 7.35E-06 | 7.37E-02 |
| Kcnn4 | 5.88E-07 | 7.35E-06 | 7.37E-02 |
| Dars | 6.06E-07 | 7.56E-06 | 7.36E-02 |
| Dirc2 | 6.06E-07 | 7.56E-06 | 7.36E-02 |
| Fam3c | 6.07E-07 | 7.56E-06 | 7.36E-02 |
| Mrpl14 | 6.08E-07 | 7.57E-06 | 7.36E-02 |
| Ndufaf2 | 6.09E-07 | 7.58E-06 | 7.35E-02 |
| AI413582 | 6.11E-07 | 7.59E-06 | 7.35E-02 |
| Nono | 6.12E-07 | 7.60E-06 | 7.35E-02 |
| Trabd | 6.13E-07 | 7.61E-06 | 7.35E-02 |
| Rrs1 | 6.16E-07 | 7.63E-06 | 7.35E-02 |
| Creg2 | 6.16E-07 | 7.63E-06 | 7.35E-02 |

| | | | |
|---|---|---|---|
| Gnaq | 6.19E-07 | 7.66E-06 | 7.35E-02 |
| Emilin1 | 6.20E-07 | 7.66E-06 | 7.35E-02 |
| Man2a1 | 6.20E-07 | 7.66E-06 | 7.35E-02 |
| Mrpl50 | 6.21E-07 | 7.67E-06 | 7.35E-02 |
| Snx14 | 6.23E-07 | 7.68E-06 | 7.35E-02 |
| Mrps26 | 6.27E-07 | 7.73E-06 | 7.35E-02 |
| Hsbp1 | 6.33E-07 | 7.79E-06 | 7.34E-02 |
| Bsg | 6.36E-07 | 7.82E-06 | 7.34E-02 |
| Neurl2 | 6.45E-07 | 7.91E-06 | 7.34E-02 |
| Rnaseh2b | 6.53E-07 | 8.00E-06 | 7.34E-02 |
| Ptp4a2 | 6.57E-07 | 8.05E-06 | 7.33E-02 |
| Elof1 | 6.60E-07 | 8.07E-06 | 7.33E-02 |
| Vcl | 6.67E-07 | 8.13E-06 | 7.33E-02 |
| Exoc3l4 | 6.69E-07 | 8.15E-06 | 7.33E-02 |
| Med28 | 6.69E-07 | 8.15E-06 | 7.33E-02 |
| Rps27l | 6.72E-07 | 8.17E-06 | 7.33E-02 |
| Fkbp3 | 6.74E-07 | 8.19E-06 | 7.33E-02 |
| Hspbp1 | 6.77E-07 | 8.21E-06 | 7.32E-02 |
| Myl6 | 6.80E-07 | 8.25E-06 | 7.32E-02 |
| Sav1 | 6.83E-07 | 8.28E-06 | 7.32E-02 |
| Stxbp2 | 6.88E-07 | 8.33E-06 | 7.32E-02 |
| Hmox2 | 6.91E-07 | 8.35E-06 | 7.32E-02 |
| Gsr | 6.92E-07 | 8.35E-06 | 7.32E-02 |
| 1500011K16Rik | 6.93E-07 | 8.35E-06 | 7.32E-02 |
| Usp16 | 6.95E-07 | 8.38E-06 | 7.32E-02 |
| Rp9 | 6.97E-07 | 8.39E-06 | 7.32E-02 |
| Tmem167 | 6.99E-07 | 8.41E-06 | 7.32E-02 |
| Eva1b | 7.00E-07 | 8.41E-06 | 7.32E-02 |
| Pwwp2b | 7.04E-07 | 8.45E-06 | 7.31E-02 |
| Tuba1c | 7.09E-07 | 8.50E-06 | 7.31E-02 |
| Ubxn8 | 7.10E-07 | 8.51E-06 | 7.31E-02 |
| Med25 | 7.12E-07 | 8.53E-06 | 7.31E-02 |
| Aggf1 | 7.15E-07 | 8.55E-06 | 7.31E-02 |
| Dpy30 | 7.20E-07 | 8.59E-06 | 7.31E-02 |
| Zc3h15 | 7.27E-07 | 8.67E-06 | 7.30E-02 |
| Lztr1 | 7.30E-07 | 8.70E-06 | 7.30E-02 |
| Ndrg1 | 7.35E-07 | 8.75E-06 | 7.30E-02 |
| Gas2l1 | 7.40E-07 | 8.80E-06 | 7.30E-02 |
| Abcf2 | 7.56E-07 | 8.97E-06 | 7.29E-02 |
| Svbp | 7.65E-07 | 9.06E-06 | 7.29E-02 |
| Ubfd1 | 7.69E-07 | 9.09E-06 | 7.29E-02 |
| Map2k3 | 7.76E-07 | 9.16E-06 | 7.29E-02 |
| Csnk2a1 | 7.82E-07 | 9.22E-06 | 7.28E-02 |
| Prkaca | 7.86E-07 | 9.26E-06 | 7.28E-02 |

| | | | |
|---|---|---|---|
| Fbxl15 | 7.86E-07 | 9.26E-06 | 7.28E-02 |
| Mrpl28 | 7.92E-07 | 9.32E-06 | 7.28E-02 |
| Qars | 7.97E-07 | 9.37E-06 | 7.28E-02 |
| Yif1a | 7.98E-07 | 9.37E-06 | 7.28E-02 |
| Mitf | 7.98E-07 | 9.37E-06 | 7.28E-02 |
| Fam32a | 7.99E-07 | 9.37E-06 | 7.28E-02 |
| Usp9x | 8.04E-07 | 9.42E-06 | 7.28E-02 |
| Rab35 | 8.09E-07 | 9.47E-06 | 7.27E-02 |
| Dgcr6 | 8.19E-07 | 9.57E-06 | 7.27E-02 |
| Ubr7 | 8.20E-07 | 9.57E-06 | 7.27E-02 |
| Gcsh | 8.21E-07 | 9.57E-06 | 7.27E-02 |
| Mrps18a | 8.25E-07 | 9.61E-06 | 7.27E-02 |
| Colgalt1 | 8.31E-07 | 9.68E-06 | 7.27E-02 |
| Mcoln3 | 8.32E-07 | 9.68E-06 | 7.27E-02 |
| Snx27 | 8.35E-07 | 9.71E-06 | 7.26E-02 |
| Abca1 | 8.39E-07 | 9.75E-06 | 7.26E-02 |
| Tmem230 | 8.44E-07 | 9.80E-06 | 7.26E-02 |
| Lars2 | 8.59E-07 | 9.95E-06 | 7.26E-02 |
| Dnajc7 | 8.63E-07 | 9.99E-06 | 7.26E-02 |
| Nagpa | 8.64E-07 | 9.99E-06 | 7.26E-02 |
| Slc38a1 | 8.64E-07 | 9.99E-06 | 7.25E-02 |

**Table S10.** List of main cholesterol esters (CE) and oxidized CE detected in lesions of 20-week-old $LDAH^{+/+}/apoE^{-/-}$ (n=6) and $LDAH^{-/-}/apoE^{-/-}$ (n=5) male littermate mice. Formulas and candidate lipids were obtained from m/z values inputs into LIPID MAPS "Bulk" Structure Searches tool. Two-tailed Mann-Whitney U test was used for CE 18:3, CE 20:5, and CE 22:6;O. All other comparisons were performed using two-tailed unpaired *t*-test.

| m/z | Abduct | Formula | Lipid | Ratio Tg/WT | p-value |
|---|---|---|---|---|---|
| 663.5477 | $K^+$ | C43H76O2K | CE 16:0 | 0.464 | 0.0099 |
| 661.532 | $K^+$ | C43H74O2K | CE 16:1 | 0.460 | 0.1465 |
| 691.5789 | $K^+$ | C45H80O2K | CE 18:0 | 0.407 | 0.0199 |
| 689.5633 | $K^+$ | C45H78O2K | CE 18:1 | 0.625 | 0.0838 |
| 687.5476 | $K^+$ | C45H76O2K | CE 18:2 | 0.739 | 0.1372 |
| 685.5319 | $K^+$ | C45H74O2K | CE 18:3 | 0.612 | 0.0519 |
| 715.579 | $K^+$ | C47H80O2K | CE 20:2 | 0.414 | 0.093 |
| 713.5634 | $K^+$ | C47H78O2K | CE 20:3 | 0.519 | 0.0511 |
| 711.5481 | $K^+$ | C47H76O2K | CE 20:4 | 0.649 | 0.0446 |
| 709.532 | $K^+$ | C47H74O2K | CE 20:5 | 0.642 | 0.1255 |
| 739.5792 | $K^+$ | C49H80O2K | CE 22:4 | 0.396 | 0.0239 |
| 737.5634 | $K^+$ | C49H78O2K | CE 22:5 | 0.522 | 0.062 |
| 735.5478 | $K^+$ | C49H76O2K | CE 22:6 | 0.590 | 0.0759 |
| 705.5583 | $K^+$ | C45H78O3K | CE 18:1;O | 0.476 | 0.1071 |
| 703.5425 | $K^+$ | C45H76O3K | CE 18:2;O | 0.593 | 0.1622 |
| 701.5269 | $K^+$ | C45H74O3K | CE 18:3;O | 0.651 | 0.371 |
| 727.5428 | $K^+$ | C47H76O3K | CE 20:4;O | 0.628 | 0.2468 |
| 751.5424 | $K^+$ | C49H76O3K | CE 22:6;O | 0.454 | 0.1204 |
