# Supplementary material for: Lipid droplet-associated hydrolase mobilizes stores of liver X receptor sterol ligands and protects against atherosclerosis
Source: Nat Commun. 2024 Aug 2;15:6540. doi: 10.1038/s41467-024-50949-y (PMC11297204; doi:10.1038/s41467-024-50949-y)
Supplement: Supplementary file 3 — Description of Additional Supplementary Information [file 41467_2024_50949_MOESM3_ESM.docx]

**Description of Additional Supplementary Files**

File Name: Supplementary Data 1

Description: Sequences of primers and siRNAs used in this study
