# Supplementary material for: Lipid droplet-associated hydrolase mobilizes stores of liver X receptor sterol ligands and protects against atherosclerosis
Source: Nat Commun. 2024 Aug 2;15:6540. doi: 10.1038/s41467-024-50949-y (PMC11297204; doi:10.1038/s41467-024-50949-y)
Supplement: Supplementary file 5 — Reporting Summary [file 41467_2024_50949_MOESM5_ESM.pdf]

Corresponding author(s): Antoni Paul and Young-Hwa Goo

Last updated by author(s): 07/13/2024

Reporting Summary

Nature Portfolio wishes to improve the reproducibility of the work that we publish. This form provides structure for consistency and transparency in reporting. For further information on Nature Portfolio policies, see our Editorial Policies and the Editorial Policy Checklist.

Statistics

For all statistical analyses, confirm that the following items are present in the figure legend, table legend, main text, or Methods section.

n/a	Confirmed	
☐	☒	The exact sample size (n) for each experimental group/condition, given as a discrete number and unit of measurement
☐	☒	A statement on whether measurements were taken from distinct samples or whether the same sample was measured repeatedly
☐	☒	The statistical test(s) used AND whether they are one- or two-sided<br>*Only common tests should be described solely by name; describe more complex techniques in the Methods section.*
☐	☒	A description of all covariates tested
☐	☒	A description of any assumptions or corrections, such as tests of normality and adjustment for multiple comparisons
☐	☒	A full description of the statistical parameters including central tendency (e.g. means) or other basic estimates (e.g. regression coefficient) AND variation (e.g. standard deviation) or associated estimates of uncertainty (e.g. confidence intervals)
☐	☒	For null hypothesis testing, the test statistic (e.g. F, t, r) with confidence intervals, effect sizes, degrees of freedom and P value noted<br>*Give P values as exact values whenever suitable.*
☒	☐	For Bayesian analysis, information on the choice of priors and Markov chain Monte Carlo settings
☒	☐	For hierarchical and complex designs, identification of the appropriate level for tests and full reporting of outcomes
☐	☒	Estimates of effect sizes (e.g. Cohen's d, Pearson's r), indicating how they were calculated

Our web collection on statistics for biologists contains articles on many of the points above.

Software and code

Policy information about availability of computer code

Data collection	AxioVision v. 4 (Carl Zeiss Microscopy)<br>Trimmomatic (version 0.38)<br>STAR (version 2.4.2a).<br>MAVEN software 3.6.1.<br>Lipid Mass Spectrum Analysis (LIMSA) v.1.0 software
Data analysis	Database for Annotation, Visualization and Integrated Discovery (DAVID knowledgebase v2023q4)<br>AxioVision V.4 (Carl Zeiss Microscopy)<br>Image J (v. 1.53)<br>Partek FlowBuild version11.0.24.0624<br>Cell ACT annotation tool (http://xteam.xbio.top/ACT/)<br>Metaspace (https://metaspace2020.eu/)<br>GraphPad Prism v.9.

For manuscripts utilizing custom algorithms or software that are central to the research but not yet described in published literature, software must be made available to editors and reviewers. We strongly encourage code deposition in a community repository (e.g. GitHub). See the Nature Portfolio guidelines for submitting code & software for further information.

Data

Policy information about availability of data

All manuscripts must include a data availability statement. This statement should provide the following information, where applicable:

- Accession codes, unique identifiers, or web links for publicly available datasets
- A description of any restrictions on data availability
- For clinical datasets or third party data, please ensure that the statement adheres to our policy

The RNA sequencing data generated in this study have been deposited in Gene Expression Omnibus with accession number GSE157947 at https://www.ncbi.nlm.nih.gov/geo/query/acc.cgi?acc=GSE157947. The shotgun lipidomic raw data have been deposited in Metabolomics Workbench under study IDs ST003317 and ST003316 at https://dx.doi.org/10.21228/M8TF97 and https://dx.doi.org/10.21228/M8Z823. The mouse scRNA-seq data reanalyzed in this study were downloaded from Gene Expression Omnibus under accession code GSE116271 at https://www.ncbi.nlm.nih.gov/geo/query/acc.cgi?acc=GSE116271. The sequences of primers and siRNAs used in this study are available in Supplementary Data 1. All other data supporting the findings in this study are available in Supplementary Information/Source Data files. Additional information, resources, and reagents are available from the corresponding authors upon request. Source data are provided with this paper.

Research involving human participants, their data, or biological material

Policy information about studies with human participants or human data. See also policy information about sex, gender (identity/presentation), and sexual orientation and race, ethnicity and racism.

Reporting on sex and gender	N/A
Reporting on race, ethnicity, or other socially relevant groupings	N/A
Population characteristics	N/A
Recruitment	N/A
Ethics oversight	N/A

Note that full information on the approval of the study protocol must also be provided in the manuscript.

Field-specific reporting

Please select the one below that is the best fit for your research. If you are not sure, read the appropriate sections before making your selection.

☒ Life sciences ☐ Behavioural & social sciences ☐ Ecological, evolutionary & environmental sciences

For a reference copy of the document with all sections, see nature.com/documents/nr-reporting-summary-flat.pdf

Life sciences study design

All studies must disclose on these points even when the disclosure is negative.

Sample size	The group sizes for in vivo studies includes at least 10 animals per genotype/experimental condition and sex. Both sexes were analyzed and reported independently. The group sizes are based on extensive experience by the investigators and power analyses to achieve 5% α-error with 80% power with standard deviations for the various assays varying ~15-30% of the mean.<br>Other experiments were performed at least in triplicate.
Data exclusions	No data/samples were excluded from any experiments presented in the manuscript.
Replication	All data are provided as independent biological replicates. Details with exact numbers are provided in the figures.
Randomization	Randomization was not possible because experimental groups were genetically based, i.e. determined by the genotype of littermate mice produced by heterozygous or hemyzygous breeding. To control for co-variates, all animal experiments were performed using littermates, and mice of the different genotypes remained co-housed for the duration of the studies. Littermate mice of different genotypes were also used as source or primacy cells for cell culture experiments.
Blinding	In all experiments involving morphometric analysis of of atheroma researchers were blinded to group allocation during data collection. For analysis of primary cells blinding was not always possible, as for some experiments such as analysis of protein expression by western blotting or gene expression by qPCR, it is necessary to know the identity of samples in each well or tube. However, all cells received the same treatments, and analyses were performed in batches with multiple samples of different genotypes analyzed simultaneously. Therefore, blinding would not influence the outcome of these experiments.

Reporting for specific materials, systems and methods

We require information from authors about some types of materials, experimental systems and methods used in many studies. Here, indicate whether each material, system or method listed is relevant to your study. If you are not sure if a list item applies to your research, read the appropriate section before selecting a response.

Materials & experimental systems

n/a	Involved in the study
☐	☒ Antibodies
☐	☒ Eukaryotic cell lines
☒	☐ Palaeontology and archaeology
☐	☒ Animals and other organisms
☒	☐ Clinical data
☒	☐ Dual use research of concern
☒	☐ Plants

Methods

n/a	Involved in the study
☒	☐ ChIP-seq
☒	☐ Flow cytometry
☒	☐ MRI-based neuroimaging

Antibodies

Antibodies used	-Custom polyclonal rabbit anti-mouse LDAH antibodies were generated at Bethyl Laboratories (used at dilution 1:3000 for western blotting)<br>- Anti-Lamp2/Mac3 (Santa Cruz Biotechnology, SC-19991 diluted 1:200 for immunoperoxidase, or SC-19991AF647 diluted 1:100 for immunofluorescence)<br>- Anti-COL1A1 (Sigma-Aldrich, 234167, dilution 1:50 for immunofluorescence).<br>- Anti-ABCA1 (Nobus Biologicals, NB400-105, dilution 1:1000 for western blotting)<br>- Anti-beta-Actin (Sigma-Aldrich, A5441, dilution 1:5000 for western blotting).<br>- Anti-GAPDH (Sigma-Aldrich, G9545, dilution 1:10000 for western blotting)
Validation	Custom polyclonal rabbit anti-mouse LDAH antibodies generated at Bethyl Laboratories were validated using knockout and transgenic tissues and cells.<br>All other antibodies were validated by the respective manufacturers. Quality control data can be assessed from the supplier.

Eukaryotic cell lines

Policy information about cell lines and Sex and Gender in Research

Cell line source(s)	All experiments were performed using primary macrophages derived from littermate mice of the genotypes indicated in the manuscript.
Authentication	The genotype of every mouse used to isolate primary macrophages or for any other study was assessed by PCR.
Mycoplasma contamination	Cells were not assessed for mycoplasma.
Commonly misidentified lines<br>(See ICLAC register)	N/A

Animals and other research organisms

Policy information about studies involving animals; ARRIVE guidelines recommended for reporting animal research, and Sex and Gender in Research

Laboratory animals	All animal studies were performed using littermates generated from heterozygous or hemyzygous breeding pairs. All mice had free access to food and water with frequent cage changes. Mice were housed under regular 12/12 light-dark cycles and under regulated temperature of 20-26 °C and a humidity level of 30-70%. Animal care and oversight was performed by the Albany Medical College Animal Resources Facility.<br><br>LDAH transgenic mice in the B6D2F1 background were generated at the Rodent Genetic Engineering Laboratory Technologies at the NYU Langone Medical Center. LDAH expression is driven by a myeloid-specific promoter. The construct contains the proximal promoter and first intron of the mouse Csf1r gene, the gene that encodes the receptor for macrophage colony-stimulating factor (CSF1) and includes a critical intronic enhancer element (FIRE). Positive founders were identified by PCR and crossed 7 times with C57BL/6 mice, followed by two additional crossings with Apoe−/− mice (also in C57BL/6 background) to generate LdahTg/0Apoe−/− mice. These mice were used as breeders to produce LdahTg/0Apoe−/− and Ldah0/0Apoe−/− littermates for atherosclerosis studies. Ldah−/− mice in the C57BL/6 background were generated by homologous recombination at the KOMP repository (www.komp.org), using an expression-selection cassette to replace the first two coding exons (exons 2 and 3) and part of exon 4. Heterozygous F1 mice were identified by PCR, and complete knockout in homozygous was confirmed by PCR and western blotting. Ldah−/− mice were crossed twice with Apoe−/− mice to generate Ldah+/−Apoe−/− mice, which were then used as breeders to produce littermate Ldah+/+Apoe−/− and Ldah−/−Apoe−/− mice.

Wild animals	N/A
Reporting on sex	Sex was addressed in the experiments, and males and females were analyzed and reported independently.
Field-collected samples	N/A
Ethics oversight	All animal studies were approved by the Albany Medical College Institutional Animal Care and Use Committee, protocol number 20-11001.

Note that full information on the approval of the study protocol must also be provided in the manuscript.

Plants

Seed stocks	N/A
Novel plant genotypes	N/A
Authentication	N/A
